# BuShen HuoXue decoction improves fertility through intestinal *hsp-16.2*-mediated heat-shock signaling pathway in *Caenorhabditis elegans*

**DOI:** 10.3389/fphar.2023.1210701

**Published:** 2023-06-02

**Authors:** Kanglu Wu, Xudong Zhao, Xian Xiao, Miao Chen, Liang Wu, Chao Jiang, Jing Jin, Lei Li, Qinli Ruan, Jun Guo

**Affiliations:** ^1^ School of Medicine, Holistic Integrative Medicine, Nanjing University of Chinese Medicine, Nanjing, China; ^2^ Department of General Practice, Affiliated Hospital of Xuzhou Medical University, Xuzhou, China; ^3^ School of Pharmacy, Nanjing University of Chinese Medicine, Nanjing, China; ^4^ Jiangsu Provincial Hospital of Traditional Chinese Medicine, Nanjing, China

**Keywords:** Bushen Huoxue decoction, fertility, HSP-16.2, intestinal barrier, *C. elegans*

## Abstract

**Introduction:** BuShen HuoXue (BSHX) decoction is commonly used in the clinical treatment of premature ovarian failure because it can increase estradiol level and decrease follicle-stimulating hormone level. In this study, we determined the potential therapeutic effects of BSHX decoction *via* anti-stress pathway and the underlying mechanism by using the nematode *Caenorhabditis elegans* as an assay system.

**Methods:** Bisphenol A (BPA, 175 μg/mL) was used to establish a fertility-defective *C. elegans* model. Nematodes were cultivated according to standard methods. Brood size, DTC, the number of apoptotic cells and oocytes were used to evaluate the fertility of nematodes. Nematodes were cultivated at 35°C as heat stress. RNA isolation and RT-qPCR were used to detect the mRNA expression level of genes. Intestinal ROS and intestinal permeability were used to evaluate the function of intestinal barrier. BSHX decoction was extracted with water and analyzed by LC/Q-TOF.

**Results and Discussion:** In BPA-treated N2 nematodes, 62.5 mg/mL BSHX decoction significantly improved the brood size and the oocytes quality at different developmental stages. BSHX decoction improved resistance to heat stress through the *hsf-1*-mediated heat-shock signaling pathway. Further analysis showed that the decoction significantly improved the transcriptional levels of *hsf-1* downstream target genes, such as *hsp-16.1*, *hsp-16.2*, *hsp-16.41*, and *hsp-16.48*. Other than *hsp-16.2* expression in the gonad, the decoction also affected intestinal *hsp-16.2* expression and significantly reversed the adverse effects induced by BPA. Moreover, the decoction ameliorated intestinal ROS and permeability. Thus, BSHX decoction can improve fertility by increasing intestinal barrier function via *hsp-16.2*-mediated heat-shock signaling pathway in *C. elegans*. These findings reveal the underlying regulatory mechanisms of *hsp-16.2*-mediated heat resistance against fertility defect.

## 1 Introduction

In China, the female fertility rate has been steadily declining. The 2020 censuse yielded the total fertility rate of 1.3 (Yang et al., 2022). Moreover, the prevalence of female infertility in China is increasing, from 11.9% in 2007 to 15.5% in 2010 (Zhou et al., 2018). The rising prevalence rate is adversely affecting female health and is a critical reason for the declining fertility rate. Therefore, drug research for enhancing female fertility is especially important to improve female health.

BuShen HuoXue (BSHX) decoction, which contains a mixture of Chinese medicines for nourishing the kidney and activating the blood, has been reported to have an obvious curative effect in the treatment of premature ovarian failure (Xu et al., 2007). The decoction significantly increased estradiol (E2) level and sinus follicle count, while significantly decreasing the levels of follicle-stimulating hormone (FSH) and luteinizing hormone (LH) in the treatment group (Xu et al., 2007). Rodent experiments have shown that the decoction can also significantly increase E2 level and significantly reduce FSH and LH levels in rats with diminished ovarian reserve induced by *Tripterygium wilfordii* or repeated immobilization stress (Xu et al., 2009; Wang et al., 2010). These results indicate that the decoction can regulate the endocrine system of the body and promote the proliferation of ovarian granulosa cells through the hypothalamic-pituitary-adrenal axis, eventually increase the number of follicles and improve ovarian function.

Stress is a predominant factor of female infertility. In mice, traditional Chinese medicines for nourishing the kidney and activating the blood delayed aging by improving free radical metabolism (Xue et al., 1999). Both types of traditional Chinese medicine could increase superoxide dismutase and decrease malondialdehyde in blood serum and increase Nrf2 and Keap1 levels in ovarian tissue, thus ameliorating premature ovarian failure in mice with diminished ovarian reserve induced by stress (Chen et al., 2021). These results suggest that BSHX improved fertility *via* anti-oxidative effects in the mice. Therefore, BSHX may ameliorate the effects of stress, but the underlying mechanism needs to be studied to improve the therapeutic effects of the decoction.

*Caenorhabditis elegans (C. elegans)* is a multicellular eukaryote with a well-differentiated and simple reproductive system. The physiological processes of oogenesis are highly conserved in *C. elegans* and mammals. In *C. elegans*, the conserved heat-shock response (HSR), which is responsible for the anti-stress reaction, mainly regulates protein homeostasis in the cytoplasmic matrix. Heat shock factor 1 (HSF1) is the most important regulatory factor of HSR (Shi et al., 1998). Normally, HSF1 binds to the cytoplasmic chaperone heat-shock proteins HSP90 and HSP70 in its deactivated state. When the HSR signaling pathway is activated, HSF1 is disintegrated from HSP70 and HSP90 and enters the nuclear and transcriptionally regulates the downstream genes (*hsp-16.1*, *hsp-16.2*, *hsp-16.41*, *hsp-16.48*) (Shi et al., 1998; Hsu et al., 2003; Guisbert et al., 2013). In the body, HSR protects the proteome of the cytosol and can be activated non-autonomously in the cell. This enables stress sensed in one tissue to activate responses in other tissues (Taylor et al., 2014). Although BSHX has been verified to promote ovarian function (Xu et al., 2007; Xu et al., 2009; Wang et al., 2010), it is unclear whether the decoction can improve fertility through other tissues. In the study, we used a fertility-defective *C. elegans* model to assess the *in vivo* pharmacological effects of BSHX decoction and investigate the possible mechanisms involved in the effects of BSHX.

## 2 Materials and methods

### 2.1 *C. elegans* strains

Wild-type N2, transgenic strains DCL569 (mkcSi13 [*sun-1p*::*rde-1*::*sun-1*+*unc-119*]), NR350 (kzIs20 [*hlh-1p*::*rde-1*+*sur-5p*::NLS::GFP), VP303 (kbIs7 [*nhx-2p*::*rde-1*+*rol-6*]), TJ375 (*hsp-16.2p*::GFP), JK2868 [*lag-2p*::GFP + *unc-119*], OD95 (ltIs38 [*pie-1p*::GFP::PH + *unc-119*]), and mutant strain *hsf-1* (sy441), and *glp-1* (e2141) were obtained from *Caenorhabditis* Genetics Center (University of Minnesota, Minneapolis, MN, United States).

### 2.2 Preparation of BSHX decoction

The BSHX decoction is composed of *Semen cuscutae* 10 g, *Radix rehmanniae praeparata* 10 g, *Radix paeoniae alba* 10 g, *R. paeoniae rubra* 10 g, *Fructus corni* 10 g, *Salvia miltiorrhizae* 10 g, *Radix dipsaci* 10 g, and *Tortoise plastron* 10 g, which were purchased from Tong Ren Tang pharmacy, Nanjing, China. All the Chinese herbs (80 g) were soaked and decocted with 800 mL distilled water for 0.5 h. The first decoction was filtered, and the residues were sequentially extracted with water twice for another 0.5 h. The filtrates were merged and vacuum-evaporated at 62°C with a rotavapor (EYELA; Shanghai, China) to obtain a final concentrate (0.5 g/mL), which was used for the experiments and stored at 4°C.

### 2.3 Reagents

Bisphenol A (BPA) was obtained from Sigma-Aldrich (St. Louis, MO, United States); erioglaucine disodium salt, YuanYe Biotech Co., Ltd. (Shanghai, China); Reactive Oxygen Species Assay Kit (ROS Assay Kit) and TRIzol, Beyotime Biotech Co., Ltd. (Shanghai, China); Hieff^Ⓡ^ qPCR SYBR Green Master Mix, Yeasen Biotech Co., Ltd. (Shanghai, China); Phanta^Ⓡ^ Max Super-Fidelity DNA Polymerase, Vazyme Biotech Co., Ltd. (Nanjing, China); PrimeScript™ RT Master Mix and Conventional Restriction Enzymes, Takara (Beijing, China), and GelRed, SinoMol Biotech Co., Ltd. (Nanjing, China).

### 2.4 Maintenance and treatment of *C. elegans* strains

The nematodes were cultivated on nematode growth medium (NGM) plates and fed with *Escherichia coli* strain OP50, according to standard methods (Brenner, 1974). Gravid nematodes were washed off the plates into centrifuge tubes and then lysed with a bleaching mixture (0.5 M NaOH and 2.5% HClO). Age synchronous populations of L1 larvae were obtained by washing with double-distilled water and K medium (0.032 M KCI and 0.051 M NaCI) successively. The nematodes were exposed to BPA (125, 150, and 175 μg/mL) or BSHX decoction (31.25, 62.5, and 125 mg/mL) from L1 larva to young adult stage or adult stage in 12-well sterile tissue culture plates at 20°C in the presence of food. The young adults or adults were transferred to fresh NGM plates and used in the following assays.

### 2.5 Brood size assays

The nematodes were treated with BSHX decoction. A single control or treated nematode was placed onto an NGM plate with OP50. Each day, all P0 nematodes were transferred to a new NGM plate. To assay brood size, the number of offspring at all stages beyond the egg was counted. More than ten nematodes were examined per treatment.

### 2.6 Fluorescence microscopy assays

After treatment with BSHX decoction, the day 2 adult JK2868, OD95, and TJ375 nematodes were mounted on agarose pads and paralyzed with 50 mM levomisole solution, and photographs were taken with Zeiss fluorescence microscope Scope A1 (Carl Zeiss AG, Jena, Germany). The fluorescence intensity of *hsp-16.2*, the morphology of DTC, or the number of oocytes at the diakinesis stage were analyzed using imaging software ZEN (Zeiss, Oberkochen, Germany).

After treatment with BSHX decoction, the day 2 N2 adults were stained with acridine orange and paralyzed with levamisole, and photographs were obtained using Zeiss fluorescence microscope Scope A1. Apoptotic cells in the per germline were counted.

### 2.7 Heat stress treatment

The nematodes were placed on 60 mm dishes containing NGM agar, with approximately 50 nematodes per dish; the dishes were placed in a 35°C incubator for heat stress and observed every 1–2 h. The number of nematode deaths in the culture dish was recorded until the last nematode died.

### 2.8 Intestinal ROS assays

After treatment with BSHX decoction, the nematodes were collected in a microfuge tube, washed with M9 buffer, and soaked in 50 μM DCFH-DA for 60 min without light. Then, the nematodes were washed three times with M9 buffer and examined for fluorescent signals at excitation and emission wavelengths of 488 and 525 nm, respectively, under the Zeiss fluorescence microscope.

### 2.9 Intestinal permeability assays

To investigate the effect of BSHX on intestinal permeability *in vivo*, we measured the erioglaucine disodium salt permeability of N2 nematodes. After treatment with BSHX decoction, day 2 adult nematodes were collected in a microfuge tube, washed with M9 buffer, and soaked in erioglaucine disodium salt at 20°C for 3 h. Then, the nematodes were washed with M9 buffer and examined with a stereo microscope SMZ745 (Jiangnan NOVEL, Nanjing, China).

### 2.10 RNA interference

RNA interference (RNAi) was used to generate loss-of-function RNAi phenotypes by feeding DCL569, NR350, and VP303 nematodes E. *coli* strain HT115 for RNA knockdown of *hsp-16.2*. The DCL569, NR350, and VP303 strains are sensitive to RNAi limited to the germline, muscle, and intestine, respectively. Following RNAi of *hsp-16.2*, nematodes were fed E. *coli* strain HT115. L1 larvae were incubated on plates containing RNAi or a control vector at 20°C until the nematodes became gravid. Gravid adult were then splited with lysis solution to synchronize a second-generation RNAi population. *Escherichia coli* strain HT115 carrying the RNAi empty vector L4440 was used as a control. RNAi efficiency was determined by quantitative real-time polymerase chain reaction (qRT-PCR) (Supplementary Figure S1). The RNAi primers are listed in Supplementary Table S1.

### 2.11 RNA isolation and RT-qPCR

After treatment with BSHX decoction, day 2 adult nematodes were collected in a microfuge tube, and total RNA was isolated with Beyozol reagent (Beyotime Institute of Biotechnology) and reverse-transcribed into cDNA by using PrimeScript™ RT Master Mix (Takara Bio, Inc., Shiga, Japan). Transcript levels of the genes of interest were normalized to that of *act-3* as the control. The cDNA products were amplified using qRT-PCR with LightCycler^®^ RNA Master SYBR Green I (Roche Life Science, Penzberg, Germany), and the primers are listed in Supplementary Table S1.

### 2.12 *Caenorhabditis elegans* sample collection for LC/Q-TOF

The samples were generated using a modification of a method described previously (Mark et al., 2016). The nematode pellets were sonicated, and the lysates were transferred to glass centrifuge tubes. Diethyl ether (2.5:1) was added to the lysate sample, which was vortexed. The mixture was centrifuged for 10 min at 300 rpm, and the upper layer was aspirated into a fresh glass tube. Then, the ether extraction procedure was repeated. The resulting extracts were dried under nitrogen and resuspended in DMSO for LC/Q-TOF.

### 2.13 Analysis of the main phytochemical components in BSHX decoction and BSHX-treated nematodes by using LC/Q-TOF

The authentic standards of catalpol (batch number: B21678), verbascoside (batch number: B20715), tanshinone IIA (batch number: B20257), salvianolic acid B (batch number: B20261), loganin (batch number: B20822), asperosaponinⅥ (batch number: B20204), paeoniflorin (batch number: B21148), and quercetin (batch number: B20527) were purchased from Yuanye Bio-Technology Limited Corporation, Shanghai. The purities of all these compounds were >98%.

The contents of catalpol, verbascoside, tanshinone IIA, salvianolic acid B, loganin, asperosaponinⅥ, paeoniflorin and quercetin in BSHX decoction and BSHX-treated nematodes were determined with Agilent 6546 LC/Q-TOF (Agilent Technologies, United States of America).

Chromatographic separation was performed with an Agilent Zorbax SB-C18 column (2.1 × 150 mm, 3.5 μm) at 35°C by using a gradient mobile phase containing 0.1% formic acid in water (solvent A) and acetonitrile (solvent B). The linear gradients were as follows: 5% B for 0–3 min, 5%–20% B for 3–16 min, 20%–50% B for 16–24 min, 50%–95% B for 24–32 min, 95% B for 32–34 min, 95%–5% B for 34–35 min, and 5% B for 35–40 min. The mass operation parameters were set as follows: a Dual AJS ESI source was used, and Gas Temp was set at 350°C, Drying Gas at 10 L/min, Nebulizer at 35 psi, Sheath Gas Temp at 350°C, Sheath Gas Flow at 11 L/min, VCap at 3500 V, Nozzle Voltage at 1000 V, and Fragmentor at 150 V. Full-scan mass spectra were acquired in negative ion mode in the mass range of *m/z* 100–1,500. The mobile phases were eluted at 0.25 mL/min, and the total injection volume was 2 μL.

### 2.14 Statistical analysis

All data in this study are expressed as mean ± standard error of the mean (SEM). Statistical analyses were performed using SPSS 12.0 (SPSS Inc., Chicago, IL, United States). The survival data were analyzed using the log-rank test. Differences between groups were identified using analysis of variance and *post hoc* multiple comparisons were performed with Dunnett’s *t-*test. The significance of differences between two groups was determined using the independent samples *t*-test. A *p*-value of ≤0.05 was considered statistically significant.

## 3 Results

### 3.1 Chemical composition of BSHX decoction and BSHX-treated nematodes

BSHX is composed of eight types of Chinese herbs. According to the Chinese Pharmacopeia edited in 2020, we selected the recommended index components from 6 types of herbs for LC/Q-TOF detection: catalpol and verbascoside from *R. rehmanniae praeparata*, tanshinone IIA and salvianolic acid B from *Salviae miltiorrhizae*, loganin from *F. corni*, asperosaponin Ⅵ from *R. dipsaci*, and paeoniflorin from *R. paeoniae alba* and *R. paeoniae rubra*. Hyperoside and quercetin are the priciple components of *S. cuscutae*. Hyperoside has a certain effect on the reproductive ability of rat model (Wei et al., 2020a; Wei et al., 2020b). Quercetin has protective effects on the reproductive system, and the component analysis of Bushen Zhuyun decoction (a kind of decoction used to the treatment for luteal phase defects and infertility, and *S. cuscutae* is one ingredients of the recipe) revealed that just quercetin not hyperoside was found in rat exposed to Bushen Zhuyun decoction (Jiang et al., 2018; Cao et al., 2020; Xu et al., 2022). Thus, We used quercetin as the index component from *S. cuscutae*.

**FIGURE 1:**
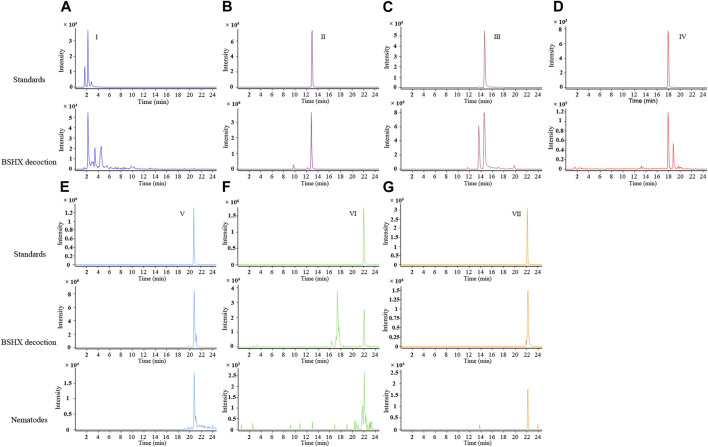
Extracted ion chromatograms (EICs) of the representative active ingredients identified in BSHX decoction and sample of BSHX-treated nematodes by LC/Q-TOF operated in the negative ionization mode. **(A–D)** The different components are as follows: I, catalpol; II, loganin; III, paeoniflorin; and IV, verbascoside; Upper panel: Standards, lower panel: BSHX decoction. **(E–G)** The different components are as follows: Ⅴ, salvianolic acid B; VI, quercetin; VII, asperosaponin VI; Upper panel: Standards, middle panel: BSHX decoction, and lower panel: BSHX-treated nematodes. BSHX, Bushen Huoxue.

Using LC/Q-TOF, the phytochemicals in the BSHX decoction were detected: paeoniflorin, loganin, salvianolic acid B, asperosaponinⅥ, verbascoside, catalpol, and quercetin (Figure 1). We found salvianolic acid B, quercetin, and asperosaponin Ⅵ in the BSHX-treated nematodes (Figures 1E–G). The qualitative information of the components identified is listed in Supplementary Table S2.

### 3.2 BSHX decoction improves the fertility of nematodes treated with BPA

**FIGURE 2:**
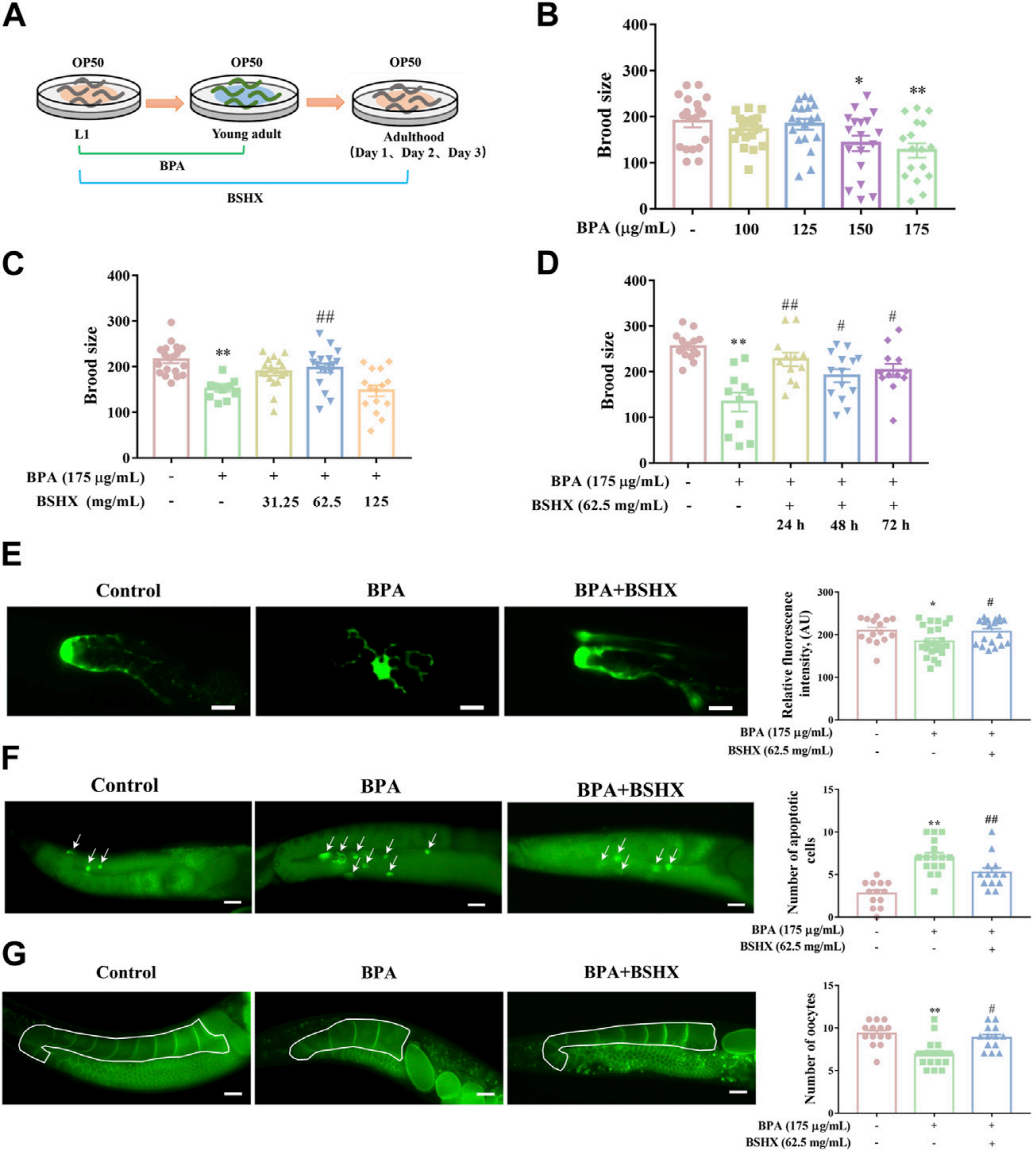
Effects of BSHX decoction administration on brood size of the fertility-defective nematodes. **(A)** Diagram of BPA and BSHX administration to the nematodes. **(B)** Effects of BPA exposure on brood size. **(C)** Effects of BSHX on fertility-defective nematodes. **(D)** Effects of BSHX on fertility-defective nematodes on each spawning day. **(E)** Left panel: Representative images of DTC in JK2868 and JK2868 nematodes treated with BPA and BPA as well as BSHX (scale bar, 10 μm); right panel: Histogram of the fluorescence intensity of DTC. **(F)** Left panel: Representative images of apoptotic cells in N2 nematodes and N2 nematodes treated with BPA and BPA as well as BSHX (scale bar, 20 μm), with arrows pointing to apoptotic cells; right panel: Histogram of the number of apoptotic cells. **(G)** Left panel: Representative images of oocytes in the diakinesis stage in OD95 and OD95 nematodes treated with BPA and BPA as well as BSHX (scale bar, 20 μm); the drawn frame is the observed gonad arm; right panel: Histogram of the number of oocytes in the diakinesis stage. BPA, Bisphenol A. BSHX, BuShen HuoXue. Bars represent means ± SEM. *p < 0.05 vs. the control group. #*p* < 0.05 vs. the BPA-treated group. BSHX, Bushen Huoxue. BPA, Bisphenol A.

BPA is an environmental endocrine disruptor and belongs to persistent organic pollutants (POPs). POPs ususally exist at low doses in external environment for a long time. BPA exposure significantly impairs oogenesis in rodents and nematodes, including decreased quality and production of oocytes (Susiarjo et al., 2007; Allard and Colaiácovo, 2010). In the study, considering the characteristics of POPs, the period from L1 larva to young adult was used for BPA exposure duation (Figure 2A). As Figure 2 showed, the nematodes exposed to 175 μg/mL BPA showed a significant decrease in brood size (Figure 2B), abnormal morphogenesis of distal tip cell (DTC), increased number of apoptotic cells, and decreased number of oocytes in the diakinesis stage of per gonad arm (Figures 2E–G). Accordingly, 175 μg/mL BPA was used to establish the fertility-defective model for nematodes in the following assays.

After administration of 62.5 mg/mL BSHX from L1 larva to adult stages, the decoction significantly increased the brood size of the fertility-defective nematodes (Figure 2C). When nematodes enter adult stage, this stage has 3 days of spawning perioid. To exactly evaluate time-dependent manner of BSHX decoction, the effect of BSHX on different spawing ending, including L1-adult day 1, L1-adult day 2, and L1-adult day 3 exposure duration, were performed. The decoction significantly increased the brood size on the first, second and third days of spawning in BPA-treated nematodes (Figure 2D). The decoction reversed the damage to DTC development induced by BPA, reduced the number of apoptotic cells, and increased the number of oocytes in the diakinesis stage per gonad arm in the fertility-defective nematodes (Figures 2E–G). Therefore, BSHX decoction improved the fertility of the BPA-affected nematodes by promoting the development of oocytes at different developmental stages.

### 3.3 BSHX decoction improves resistance to heat stress through *hsf-1*-mediated heat-shock signaling pathway in fertility-defective nematodes

**FIGURE 3:**
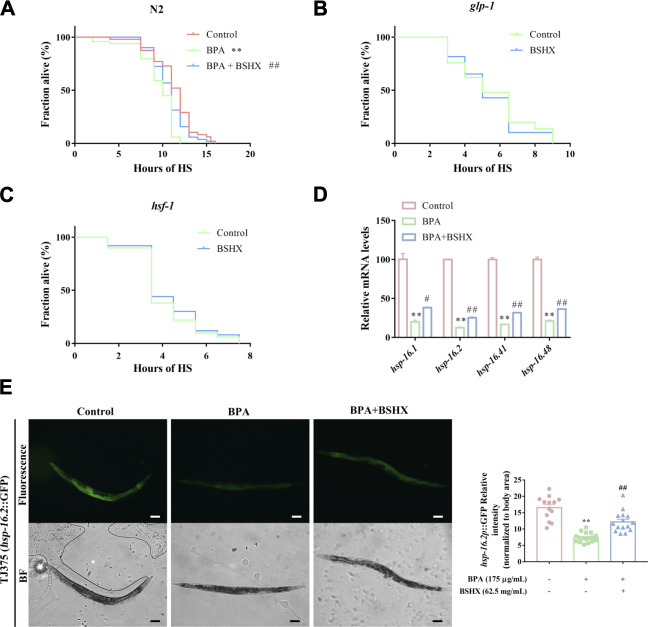
BSHX decoction improves resistance to heat stress through *hsf-1*-mediated heat-shock signaling pathway in fertility-defective nematodes. **(A)** Time course of survival in N2 nematodes treated with/without BSHX and a BPA after heat stress **(B)** Time course of survival in *glp-1* nematodes treated with/without BSHX after heat stress **(C)** Time course of survival in *hsf-1* nematodes treated with/without BSHX after heat stress **(D)** Relative mRNA levels of genes relevant to the heat-shock signaling pathway in nematodes treated with BPA and BSHX. **(E)** Left panel: Representative images of *hsp-16.2*_*p*_::GFP expression in TJ375 and TJ375 nematodes treated with BPA and BPA as well as BSHX (scale bar, 100 μm). Right panel: Histogram of *hsp-16.2*_*P*_::GFP relative fluorescence intensity/body area of the nematodes. Bars represent means SEM. *p < 0.05 vs. the control group. #*p* < 0.05 vs. the BPA-treated group. BSHX, Bushen Huoxue. BPA, Bisphenol A.

Stress has an adverse effect on fertility, and heat stress can impair somatic tissue function, resulting in premature death. Therefore, heat stress was used to study the effect of BSHX on resistance to heat stress in this study. The N2 nematodes treated with BPA showed shortened survival after heat stress when compared with the control (Figure 3A). In contrast, BSHX treatment induced significant heat-stress resistance in the BPA-treated nematodes (Figure 3A). To validate the requirement of the germline for somatic stress resistance upon BSHX treatment, we used *glp-1* (e2141) mutants that fail to develop a germline. The germline-less *glp-1* (e2141) mutants did not show elevated heat-stress resistance upon BSHX treatment (Figure 3B).

HSR is an essential stress protection mechanism, and it is evolutionarily conservative between nematodes and mammals. When the HSR signaling pathway is activated, *hsf-1* is also activated and transformed into the nuclear, active downstream *hsp-16.1*, *hsp-16.2*, *hsp-16.41*, and *hsp-16.48* (Shi et al., 1998; Hsu et al., 2003). In this study, *hsf-1* (sy441) loss-of-function mutants did not show elevated heat-stress resistance upon BSHX treatment (Figure 3C), which means *hsf-1* is involved in the heat-stress resistance upon BSHX treatment. Further investigation showed that the decoction significantly improved the transcriptional levels of the downstream target genes of *hsf-1*, such as *hsp-16.1*, *hsp-16.2*, *hsp-16.41*, and *hsp-16.48* (Figure 3D). BSHX decoction increased GFP expression in *hsp-16.2*_*P*_::*GFP* transgenic TJ375 nematodes affected by heat stress (Figure 3E). Thus, BSHX decoction improves resistance to heat stress through *hsf-1*-mediated heat-shock signaling pathway in fertility-defective nematodes.

### 3.4 BSHX decoction increases the fertility of nematodes *via* the synergistic effects of *hsp-16.2* in the gonad and intestine

HSR triggered by a tissue injury can also activate HSR in other tissues, such as the central nervous system, muscle tissue, and digestive tract. HSRs in different tissues have a synergistic effect on the stress resistance of the body.

**FIGURE 4:**
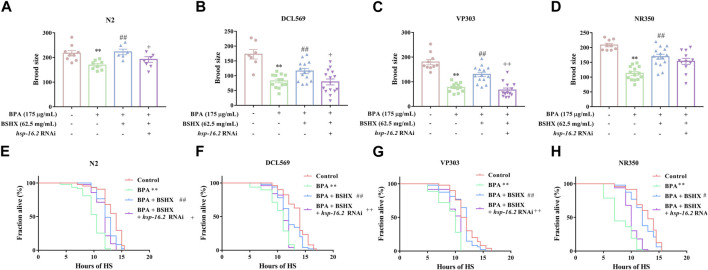
BSHX decoction improves the fertility of fertility-defective nematodes through the combined effects of *hsp-16.2* in the gonad and intestinal Tissue-specific activity of *hsp-16.2* in response to the effects of BSHX on BPA-treated nematodes in brood size, **(A)** N2 nematodes, **(B)** DCL569 nematodes (germline-specific RNAi), **(C)** VP303 nematodes (intestine-specific RNAi), and **(D)** NR350 nematodes (muscle-specific RNAi). Tissue-specific activity of *hsp-16.2* in response to the effects of BSHX on BPA-treated nematodes in heat-stress resistance, **(E)** N2 nematodes, **(F)** DCL569 nematodes (germline-specific RNAi), **(G)** VP303 nematodes (intestine-specific RNAi) and **(H)** NR350 nematodes (muscle-specific RNAi). Bars represent means ± SEM. *p < 0.05 vs. the control group. #*p* < 0.05 vs. the BPA-treated group. + *p* < 0.05 vs. the BPA and BSHX co-treated group. BSHX, Bushen Huoxue. BPA, Bisphenol A.

*Hsp-16.2*, the downstream gene of *hsf-1*, is commonly used as an important heat-stress resistance indicator. *Hsp-16.2* can be expressed in several tissues, including the intestine, muscle, and gonad. In this study, increased brood size as well as increased heat-stress resistance were observed in N2, DCL569, VP303, and NR350 nematodes cotreated with BPA and BSHX compared with BPA-treated nematodes (Figure 4). *Hsp-16.2* RNAi in N2 nematodes showed significant decrease in brood size and heat stress resistance compared with the nematodes cotreated with BPA and BSHX (Figures 4A, E). Using DCL569 for germline-specific RNAi knockdown, *hsp-16.2* RNAi knockdown suppressed the beneficial effects of BSHX on the BPA-treated nematodes (Figures 4B, F). Using VP303 for intestine-specific RNAi knockdown, *hsp-16.2* RNAi knockdown also suppressed the beneficial effects of BSHX on the BPA-treated nematodes (Figures 4C, G). In contrast, using NR350 for muscle-specific RNAi knockdown, *hsp-16.2* RNAi knockdown could not influence the effects of BSHX on the toxicity of BPA (Figures 4D, H). Thus, BSHX increased the brood size of fertility-defective nematodes through *hsp-16.2* in both the gonad and intestine.

### 3.5 BSHX decoction improves the intestinal barrier by decreasing intestinal ROS and permeability

We analyzed whether BSHX improved intestinal barrier function through *hsp-16.2*. In the study, the intestinal tracts of *C. elegans* were dissected, and the intestinal RNA was extracted. Genes related to intestinal function were detected using qRT-PCR: *clc-2* (the ortholog of mammalian claudin gene), genes related to the development of microvilli on intestinal cells (*ifb-2*, *act-5*, *mtm-6*, *par-3*, *gtl-1*, *par-6*, *pkc-3*, and *erm-1*), genes related to the development of the baso-lateral domain of the intestine (*abts-4*, *nfm-1*, and *let-413*), and genes associated with the development of the apical junction of the intestine (*dlg-1* and *egl-8*).

**FIGURE 5:**
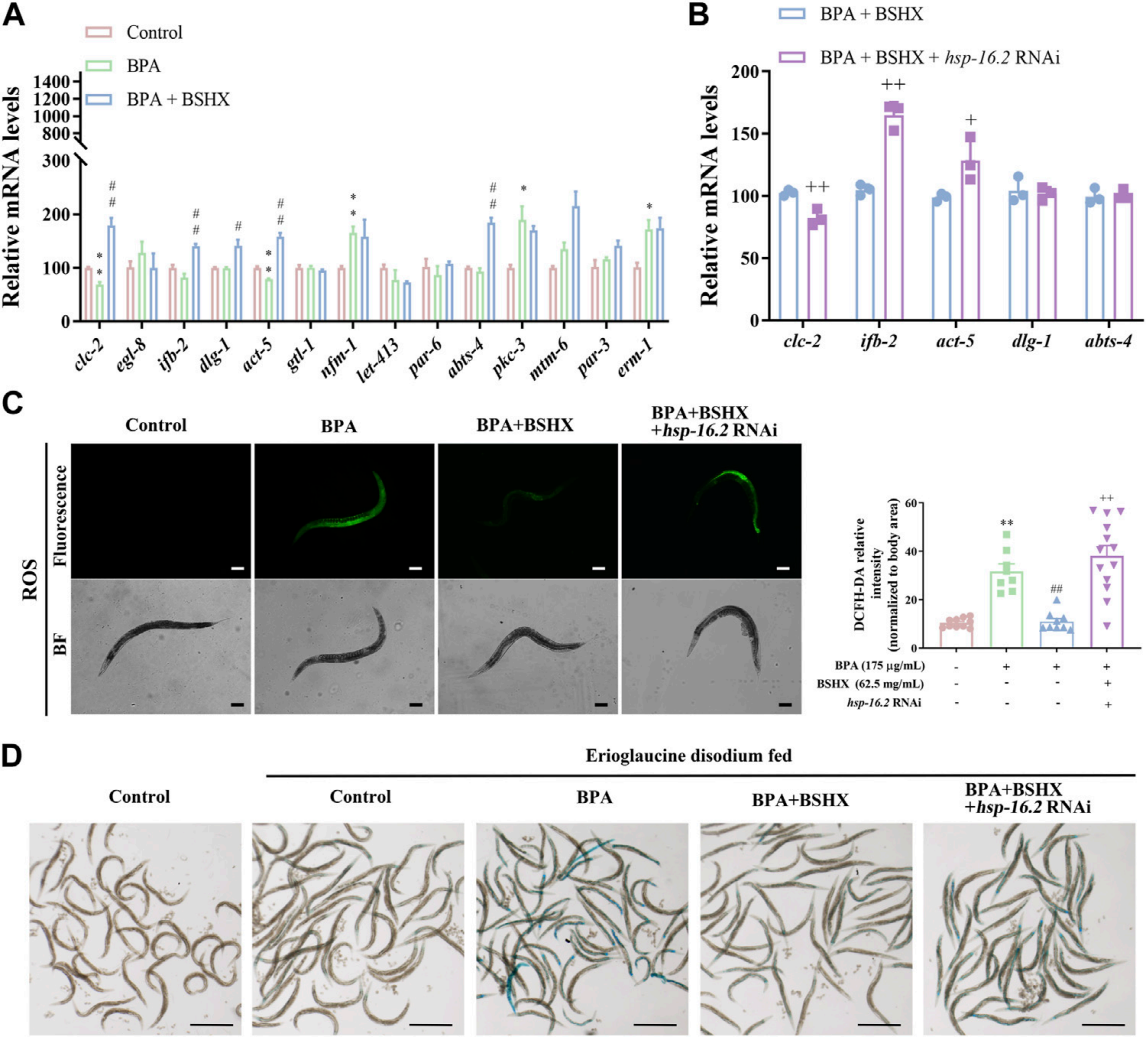
BSHX decoction improves the intestinal barrier by decreasing intestinal ROS and permeability. **(A)** Relative mRNA levels of genes relevant to intestinal function in fertility-defective nematodes treated with BSHX. **(B)** Relative mRNA levels of genes relevant to intestinal function in nematodes and *hsp-16.2* (RNAi) nematodes treated with BSHX and BPA. **(C)** Left panel: Representative images of intestinal ROS content in N2 nematodes and N2 nematodes treated with BPA, BSHX, and BPA as well as BSHX with/without *hsp-16.2* RNAi (scale bar, 100 μm); right panel: Histogram of the fluorescence intensity of intestinal ROS. **(D)** Representative images of intestinal permeability in N2 nematodes and N2 nematodes treated with BPA, BSHX, and BPA as well as BSHX with/without *hsp-16.2* RNAi (scale bar, 1 mm). Bars represent means ± SEM. *p < 0.05 vs. the control group. #*p* < 0.05 vs. the BPA-treated group. BSHX, Bushen Huoxue. BPA, Bisphenol A.

The results showed that BSHX significantly increased the transcriptional levels of *clc-2*, *ifb-2*, *dlg-1*, *act-5*, and *abts-4* in BPA-treated nematodes (Figure 5A). Next, we detected the transcriptional levels of these 5 genes in *hsp-16.2* RNAi nematodes exposed to BPA and BSHX, and the transcriptional levels of *clc-2*, *ifb-2*, and *act-5* were significantly changed (Figure 5B).

In terms of intestinal function, BPA significantly increased the intestinal ROS and permeability, but BSHX decoction significantly reversed the increase in intestinal ROS and enhancement of intestinal permeability induced by BPA (Figures 5C, D). When *hsp-16.2* in the intestine was specifically targeted with RNAi, the effects of the decoction were inhibited (Figures 5C, D). Therefore, BSHX decoction can improve fertility by strengthening the intestinal barrier *via hsp-16.2*-mediated heat-shock signaling pathway in *C. elegans*.

## 4 Discussion

Female fertility is a woman’s ability to produce oocytes, undergo normal fertilization, and carry a fetus. Generally, the indicators to evaluate a decline in female fertility mainly include hormone levels, ovarian granulosa cell proliferation, and oocyte developmental function. In the study, on the basis of the anatomical characteristics of per gonad arm in *C. elegans*, DTC, apoptotic cells, and oocytes in the diakinesis stage were selected to evaluate the effects of BSHX on oocyte development. Firstly, we selected BPA to establish the fertility-defective model. The median lethal dose of BPA in adult rats were 841 mg/kg body weight via intraperitoneal, or 35.26 mg/kg body weight via intravenous route (Pant and Deshpande, 2012). We found that 175 μg/mL BPA significantly decreased the fertility of N2 nematodes in the study, and BSHX decoction improved the fertility of fertility-defective nematodes by promoting the development of oocytes at different developmental stages.

Stress is an important cause of the decline in female fertility in China. Because *C. elegans* is an ectotherm, it can sense temperature changes and its state is affected by ambient temperature fluctuations. Therefore, the survival rate of *C. elegans* in high-temperature environments is often used as an indicator of its body’s resistance to heat stress. Currently, heat stress and toxic stress (such as paraquat) are commonly used as stressors for nematodes. In this study, we used heat stress, and the environmental endocrine disruptor BPA significantly decreased brood size and resistance to heat stress; however, BSHX reversed the toxic effects of BPA on reproduction and stress resistance. These results indicate that BSHX improves fertility by increasing resistance to heat stress. The heat stress assays using *glp-1* and *hsf-1* mutants further confirmed that germline and heat shock signaling pathway contributed to the beneficial effects of BSHX on the fertility of the nematodes treated with BPA. Next, we detected the transcriptional levels of downstream genes of *hsf-1*. The BSHX decoction increased the transcriptional levels of *hsp-16.1*, *hsp-16.2*, *hsp-16.41*, and *hsp-16.48* in BPA-treated N2 nematodes and increased the level of HSP-16.2 in BPA-treated TJ375. Therefore, BSHX can improve resistance to heat stress by activating the HSR signaling pathway.

HSR is closely associated with numerous tissue-specific and age-dependent human diseases. The induction of HSR has been shown to have protective effects in multiple models for protein conformation diseases. HSR, as a kind of system stress protection, has the characteristics of compartment-specific stress responses that allow for the targeted induction of chaperones within specific tissue cells. Neuronal overexpression of HSF1 results in the generation of a transcellular signal that activates both DAF-16 and HSF-1 in the intestine of *C. elegans* (Douglas et al., 2015; Tatum et al., 2015), which indicates HSR in one tissue can active HSR in other tissues. In this study, we used germline- or intestine-specific RNAi nematodes as a genetic tool and found that *hsp-16.2* knockdown in the gonad or intestine suppressed the effects of BSHX on brood size and resistance to heat stress in BPA-treated nematodes. Although germline is the target of BSHX, the decoction also increases fertility through HSR in the intestine. The beneficial effect of BSHX decoction is the combined effect on germline and intestine.

The intestinal tract of *C. elegans* is formed by a layer of epithelial cells and tightly sealed by tight junction proteins such as claudins and occludins. Intestinal barrier function is the first line of defense against harmful microbial pathogens and antigens. It has been shown that stress impairs the integrity of tight connections (Awad et al., 2017; Mortensen et al., 2020), thereby increasing the permeability of the intestinal mucosa and disrupting its function as a selective barrier to absorb nutrients and keep pathogens out. Activation of intestinal signaling pathway, such as TGF-β and Wnt, could mediate a protective strategy to maintain the functional state of intestinal barrier (Zhi et al., 2017; Liu et al., 2020). In this study, we detected the transcriptional levels of tight junction protein claudin gene, genes related to the development of microvilli on intestinal cells, genes related to the development of the baso-lateral domain of the intestine, and genes associated with the development of the apical junction of the intestine.

Of these genes, *clc-2* is the ortholog of mammalian claudin gene, and it is expressed in seam cells of the hypodermis. It is involved in the seaming mechanism between the seam cell syncytium and surrounding hypodermal cells. In *clc-2*-deficient nematodes, the hypodermal cell layers on the body surface became permeable to the TRITC-dextran dye (Asano et al., 2003). Microvilli are actin-based cellular structures that form plasma membrane projections into the extracellular space, and their specialized shape provides increased cellular surface area (MacQueen et al., 2005). The expression of *act-5* is limited to microvillus-containing cells within the intestine and excretory systems, and *act-5* function is essential for the stable morphogenesis of intestinal microvilli. *Ifb-2* is a structural constituent of the cytoskeleton, and it is specifically expressed in intestinal cells (Geisler et al., 2019). Increased levels of *ifb-2* promote the loss of intestinal integrity and bacterial colonization (Koyuncu et al., 2021). The results showed that BPA significantly decreased the mRNA levels of *clc-2* and *act-5* in the N2 intestine, whereas BSHX significantly increased the mRNA levels of *clc-2*, *ifb-2*, *dlg-1*, *act-5*, and *abts-4* in the BPA-treated N2 intestine. Specific RNAi knockdown of *hsp-16.2* in N2 nematodes cotreated with BPA and BSHX resulted in a significant decrease in the mRNA level of *clc-2* and a significant increase in the mRNA levels of *ifb-2* and *act-5*. Therefore, BSHX decoction improves intestinal function by activating HSR, regulating the transcriptional levels of *clc-2*, *ifb-2*, and *act-5*, and improving intestinal function and fertility.

BSHX is composed of eight types of Chinese herbs. In this study, we detected salvianolic acid B, quercetin, and asperosaponin Ⅵ in the BSHX-treated nematodes. Quercetin is the main component of *S. cuscutae*, and it promotes *in vitro* maturation of oocytes in humans and aged mice (Cao et al., 2020). Salvianolic acid B is the most bioactive component in *Salvia miltiorrhiza*, which has antioxidant, anti-inflammatory, anti-tumor, antibacterial, antiviral, and anti-fibrosis activities (Zhao et al., 2019). Asperosaponin Ⅵ is the main ingredient of *R. dipsaci*. It has a wide range of pharmacological effects, such as anti-oxidative and anti-inflammatory (Li et al., 2010; Yu et al., 2012). Thus, the three phytochemicals (salvianolic acid B, quercetin, and asperosaponin Ⅵ) are effective components of the BSHX decoction. Notably, the resistance of monochemical quercetin to heat-stress was less effective than that of BSHX decoction (Supplementary Figure S2). The impact of BSHX on fertility could be the combined effects of salvianolic acid B, quercetin, and asperosaponin Ⅵ. Therefore, we propose that ovarian development is positively associated with anti-stress factors, which promotes fertility.

## 5 Conclusion

BSHX decoction improved the fertility of *C. elegans* through the germline as well as intestine. In the intestine, BSHX decoction strengthened *hsf-1-*mediated HSR and induced heat shock pathway downstream genes such as *hsp-16.1*, *hsp-16.2*, *hsp-16.41*, and *hsp-16.48* and the resultant downstream *clc-2*, *ifb-2*, and *act-5*. The role of BSHX decoction in fertility depends on improving intestinal barrier function, decreasing intestinal ROS and permeability, and promoting intestine development.

## Data Availability

The original contributions presented in the study are included in the article/Supplementary Material, further inquiries can be directed to the corresponding author.

## Supplementary material

SUPPLEMENTARY FIGURE S1 The RNAi efficiency assessed by qRT-PCR. RNAi of *hsp-16.2* treated with N2 nematodes.

SUPPLEMENTARY FIGURE S2 Time course of survival in N2 nematodes treated with/without BPA and quercetin (que) after heat stress.

SUPPLEMENTARY TABLE S1 Primers for RNAi and qRT-PCR analyses.

SUPPLEMENTARY TABLE S2 Qualitative information of the components identified.
