# Supplementary material for: BuShen HuoXue decoction improves fertility through intestinal hsp-16.2-mediated heat-shock signaling pathway in Caenorhabditis elegans
Source: Front Pharmacol. 2023 Jun 2;14:1210701. doi: 10.3389/fphar.2023.1210701 (PMC10272376; doi:10.3389/fphar.2023.1210701)
Supplement: Supplementary file 1 [file Table15.DOCX]

Raw data for supplementary Figure S1


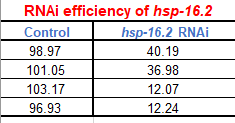


Raw data for supplementary Figure S2


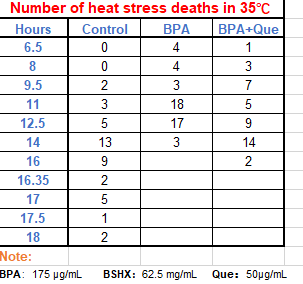


Statistic analysis

1. RNAi efficiency of *hsp-16.2*

| **Tests of Normality** | | | | | | | |
| --- | --- | --- | --- | --- | --- | --- | --- |
|  | Group | Kolmogorov-Smirnov^a^ | | | Shapiro-Wilk | | |
|  |  | Statistic | df | Sig. | Statistic | df | Sig. |
| RNAi efficiency | Control | .154 | 4 | . | .993 | 4 | .972 |
|  | *hsp-16.2* RNAi | .304 | 4 | . | .781 | 4 | .072 |
| a. Lilliefors Significance Correction | | | | | | | |

| **Group Statistics** | | | | | |
| --- | --- | --- | --- | --- | --- |
|  | Group | N | Mean | Std. Deviation | Std. Error Mean |
| RNAi efficiency | Control | 4 | 100.0270 | 2.68506 | 1.34253 |
|  | *hsp-16.2* RNAi | 4 | 25.3735 | 15.31570 | 7.65785 |

| **Independent Samples Test** | | | | | | | | | | |
| --- | --- | --- | --- | --- | --- | --- | --- | --- | --- | --- |
|  | | Levene's Test for Equality of Variances | | t-test for Equality of Means | | | | | | |
|  |  | F | Sig. | t | df | Sig. (2-tailed) | Mean Difference | Std. Error Difference | 95% Confidence Interval of the Difference | |
|  |  |  |  |  |  |  |  |  | Lower | Upper |
| RNAi efficienc | Equal variances assumed | 156.836 | .000 | 9.602 | 6 | .000 | 74.65348 | 7.77464 | 55.62962 | 93.67734 |
|  | Equal variances not assumed |  |  | 9.602 | 3.184 | .002 | 74.65348 | 7.77464 | 50.70158 | 98.60537 |

1. HS assay of Que（50 µg/mL)

| **Means and Medians for Survival Time** | | | | | | | | |
| --- | --- | --- | --- | --- | --- | --- | --- | --- |
| group | Mean^a^ | | | | Median | | | |
|  | Estimate | Std. Error | 95% Confidence Interval | | Estimate | Std. Error | 95% Confidence Interval | |
|  |  |  | Lower Bound | Upper Bound |  |  | Lower Bound | Upper Bound |
| Control | 14.564 | .342 | 13.894 | 15.235 | 14.000 | .513 | 12.994 | 15.006 |
| BPA | 11.000 | .283 | 10.444 | 11.556 | 11.000 | .287 | 10.438 | 11.562 |
| BPA+Que | 12.012 | .364 | 11.299 | 12.726 | 12.500 | .521 | 11.480 | 13.520 |
| Overall | 12.454 | .228 | 12.007 | 12.901 | 12.500 | .271 | 11.970 | 13.030 |
| a. Estimation is limited to the largest survival time if it is censored. | | | | | | | | |

| **Pairwise Comparisons** | | | | | | | |
| --- | --- | --- | --- | --- | --- | --- | --- |
|  | group | 1.00 | | 2.00 | | 3.00 | |
|  |  | Chi-Square | Sig. | Chi-Square | Sig. | Chi-Square | Sig. |
| Log Rank (Mantel-Cox) | Control |  |  | 49.061 | .000 | 23.452 | .000 |
|  | BPA | 49.061 | .000 |  |  | 8.828 | .003 |
|  | BPA+Que | 23.452 | .000 | 8.828 | .003 |  |  |
