# Supplementary figures and images for: BuShen HuoXue decoction improves fertility through intestinal hsp-16.2-mediated heat-shock signaling pathway in Caenorhabditis elegans
Source: Front Pharmacol. 2023 Jun 2;14:1210701. doi: 10.3389/fphar.2023.1210701 (PMC10272376; doi:10.3389/fphar.2023.1210701)

Control


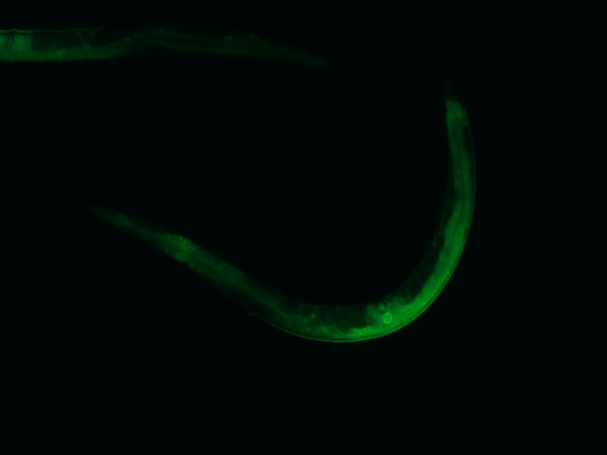

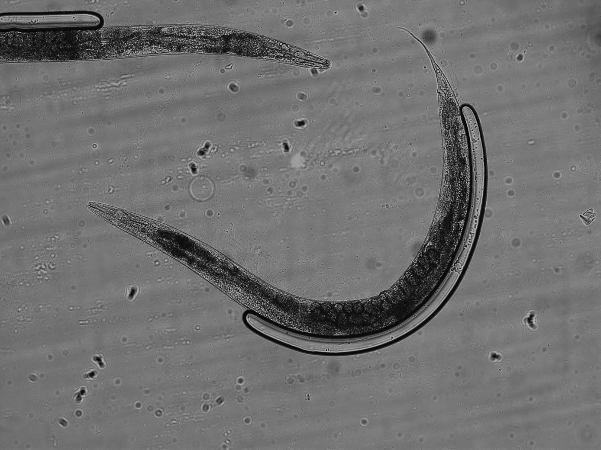

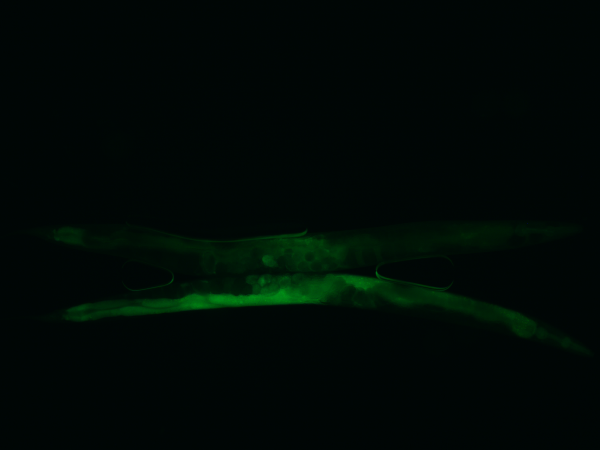

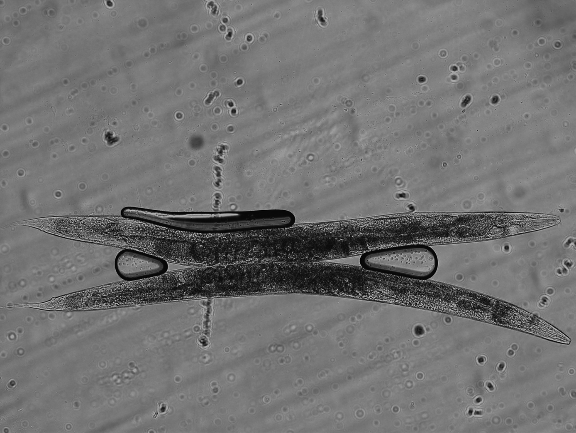

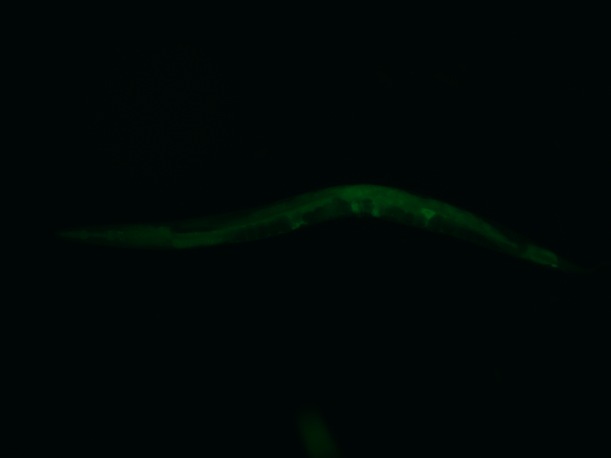

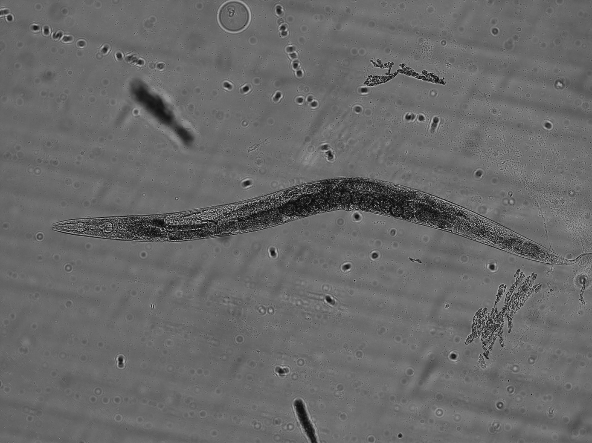


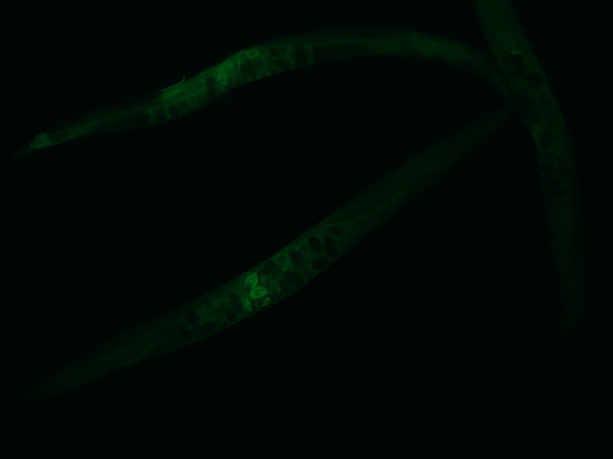


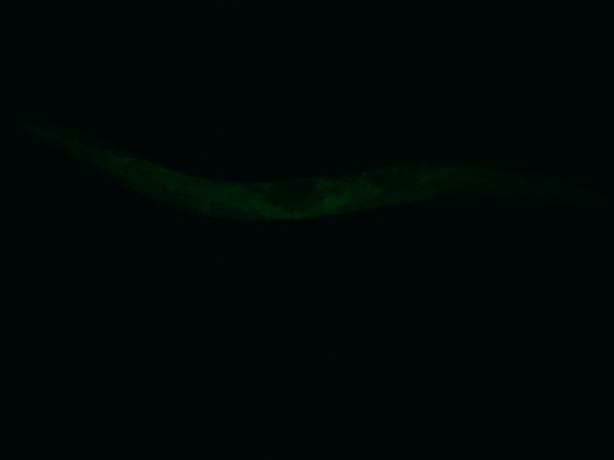

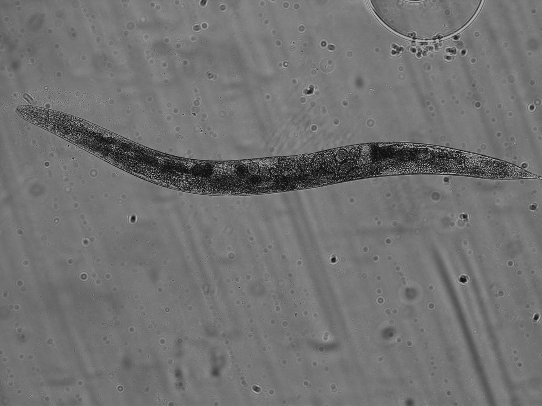

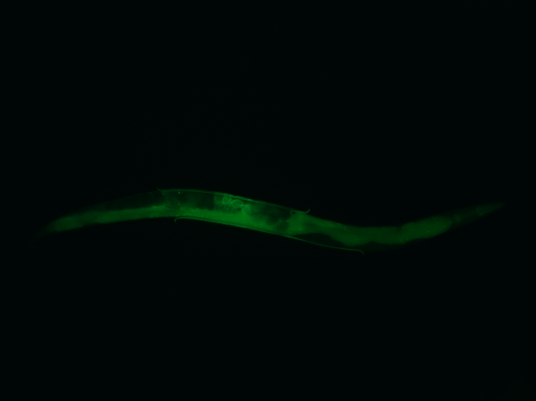

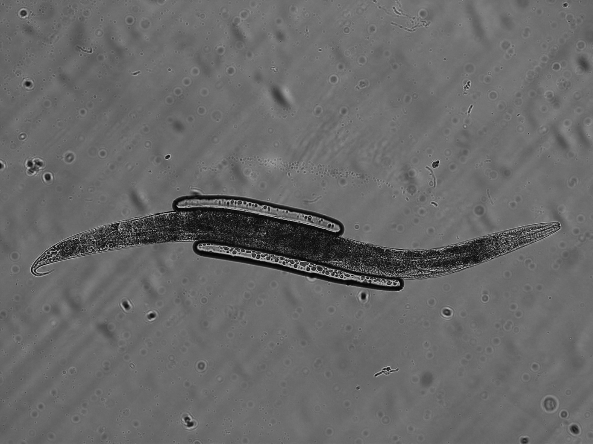

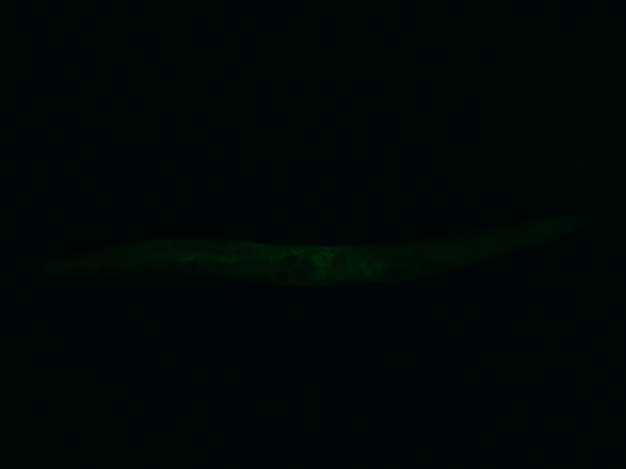

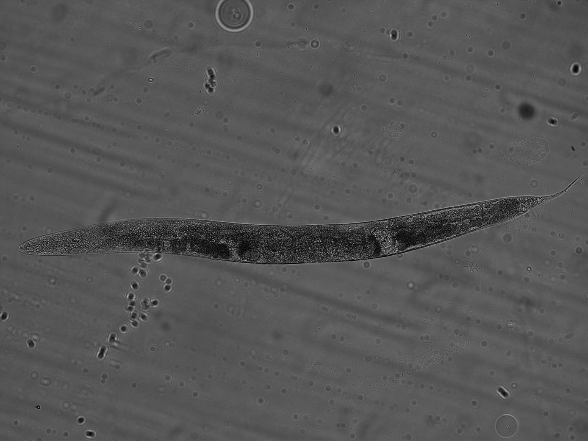

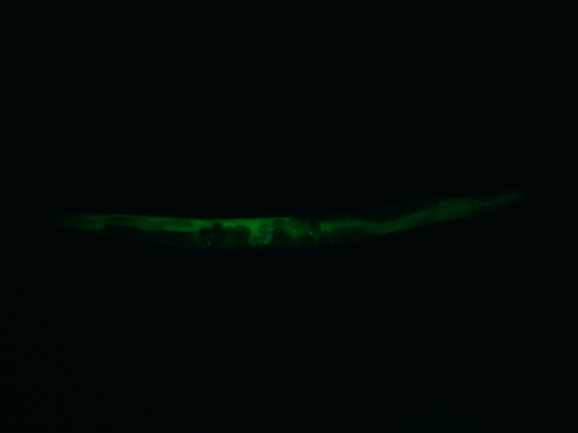

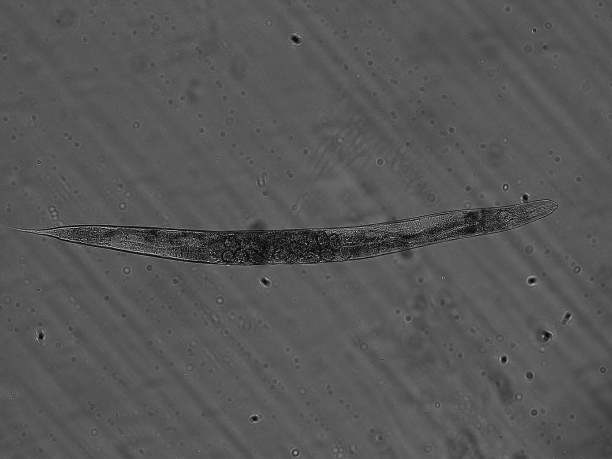

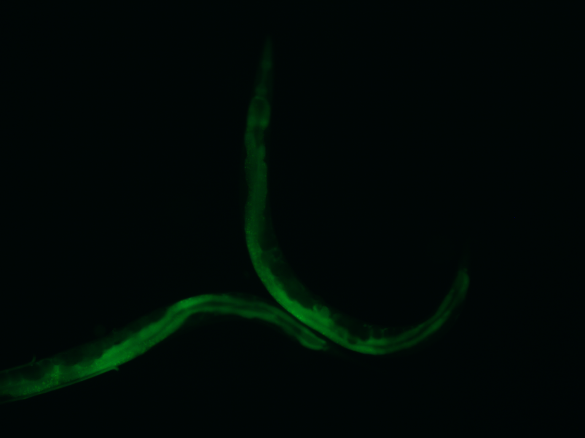

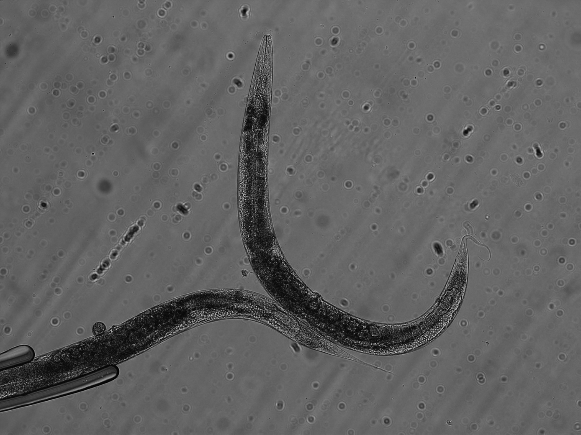

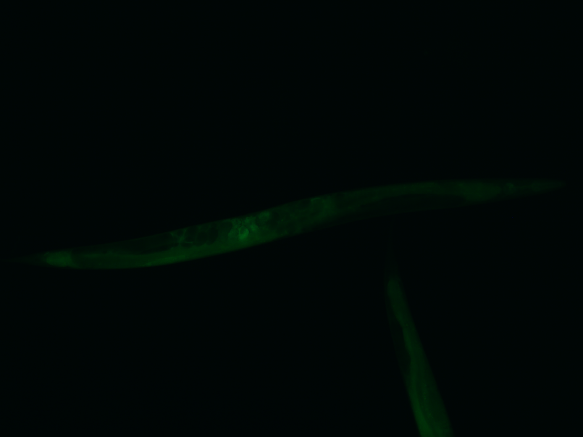

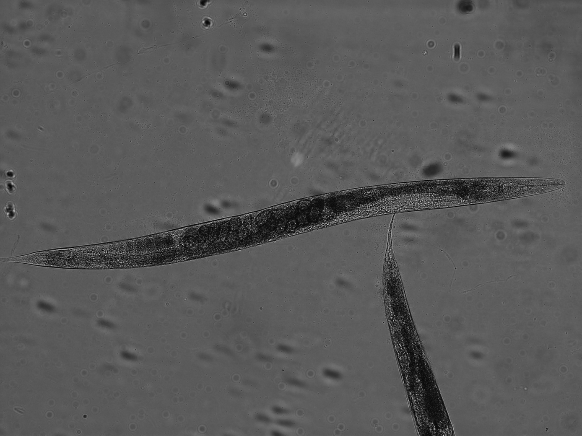

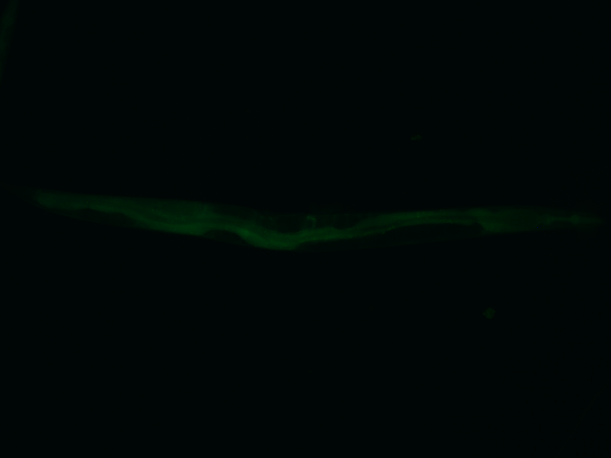

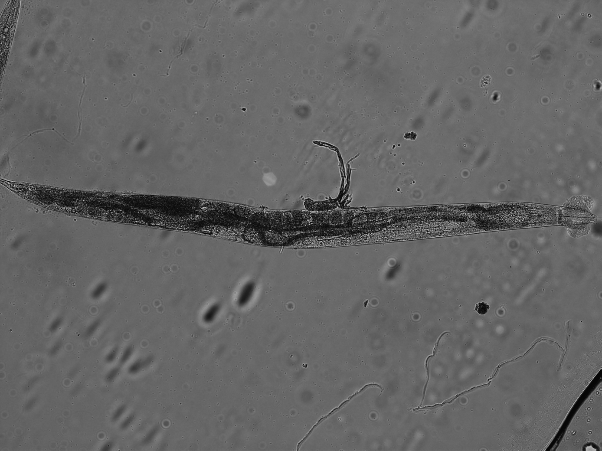

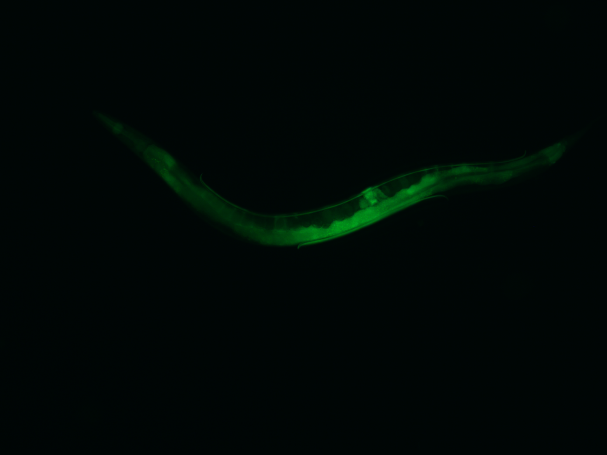

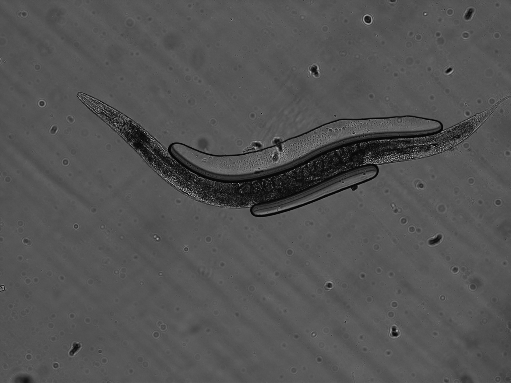

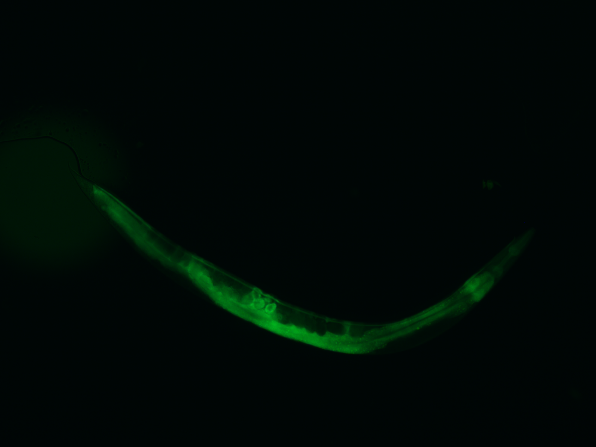

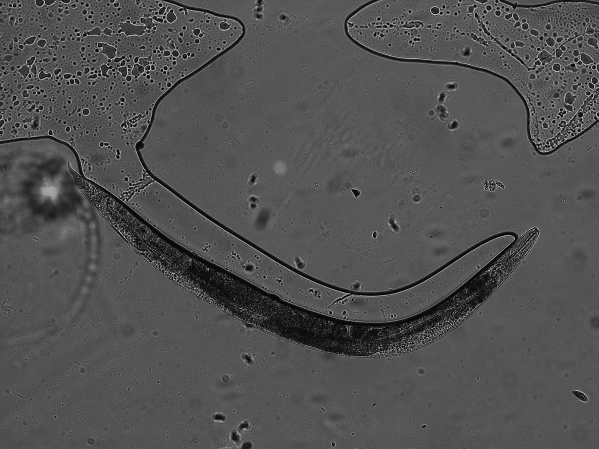


Fig. 3E BPA


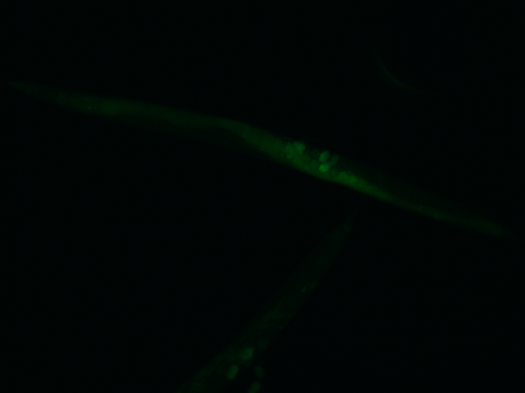

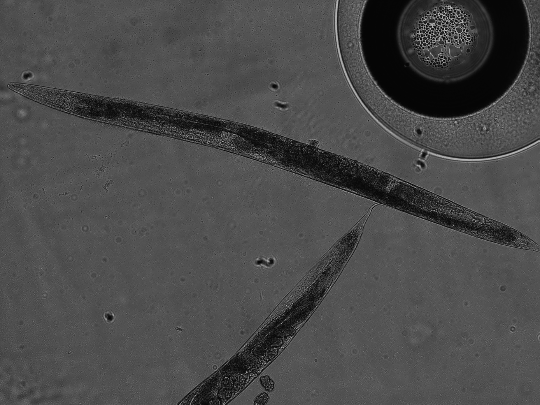

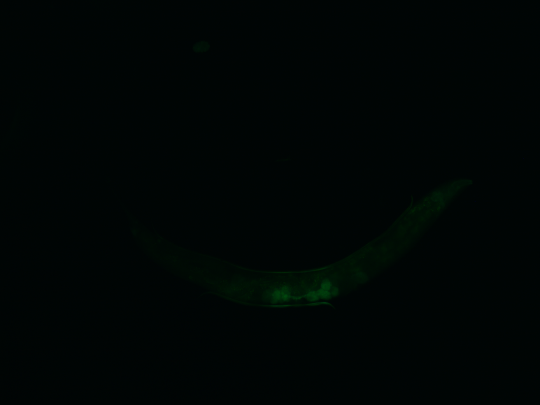


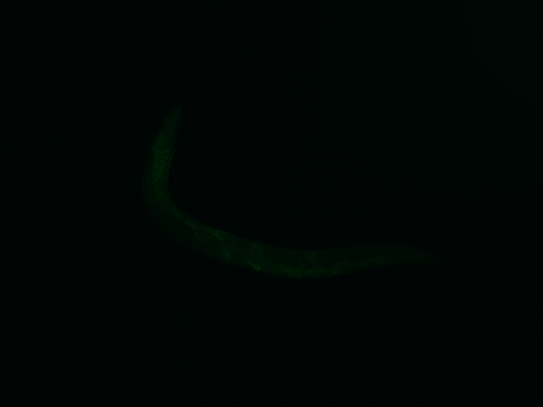

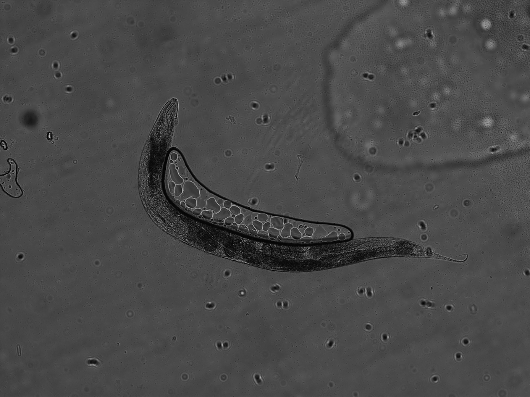

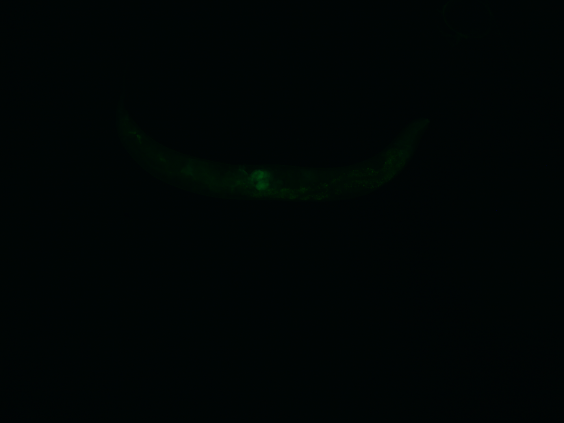

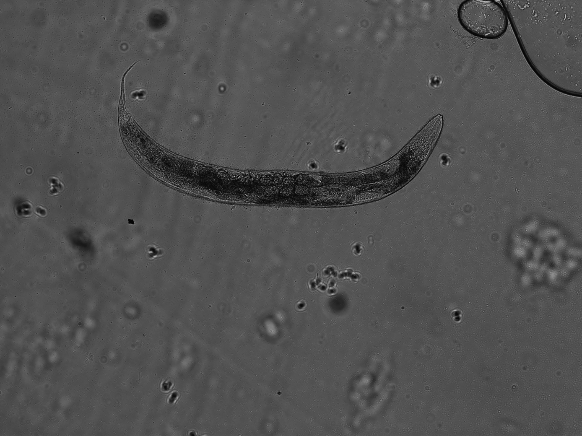

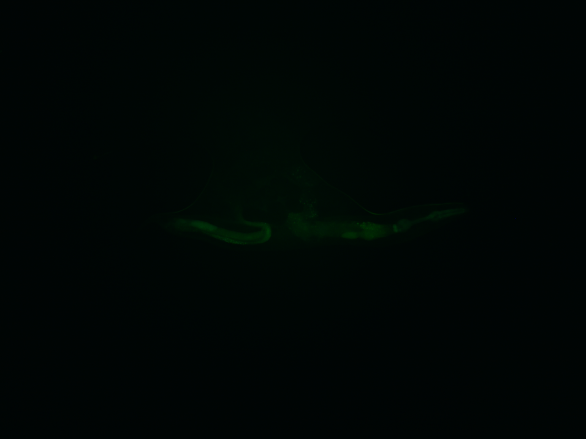

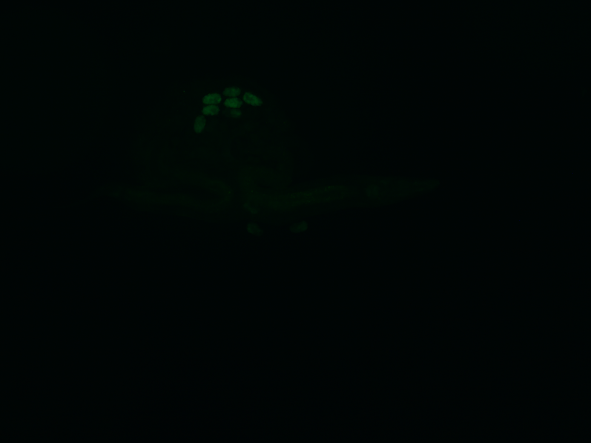

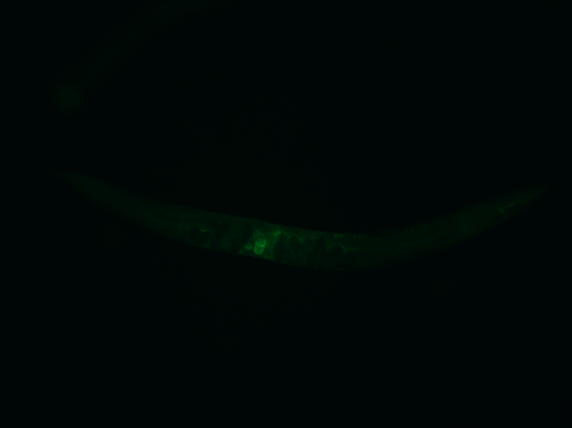

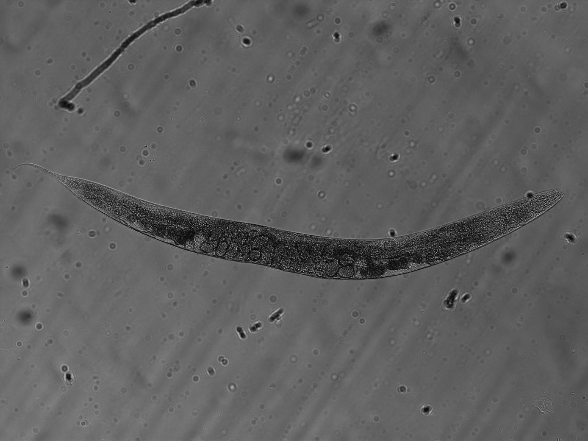

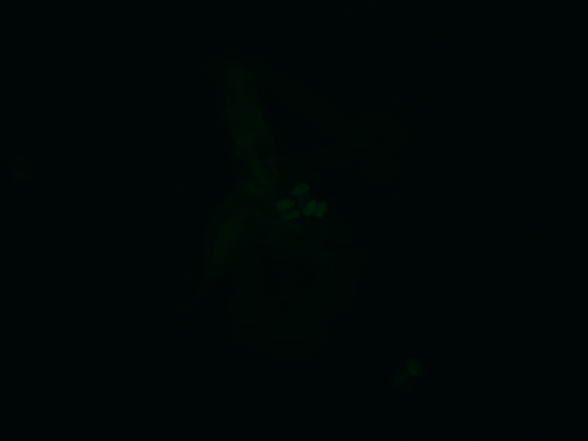

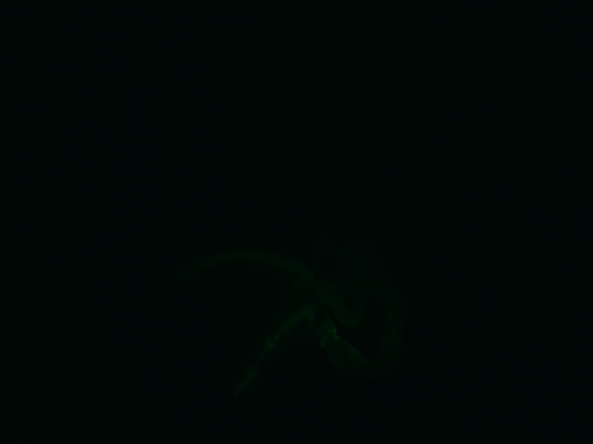

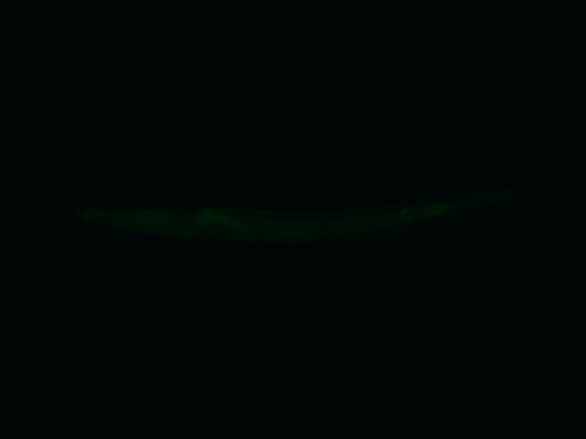

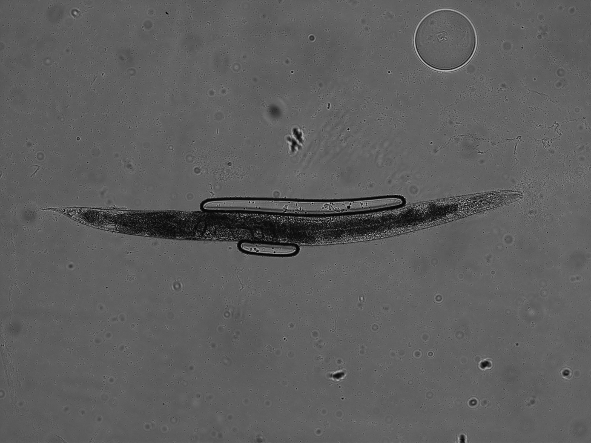

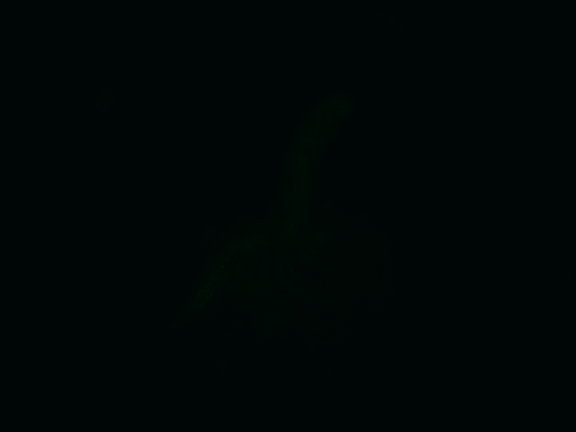

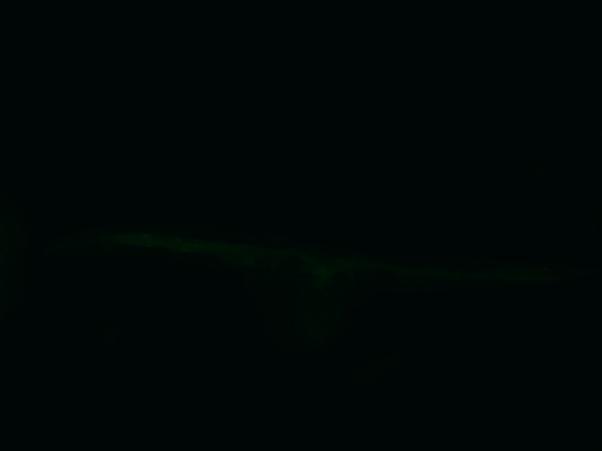

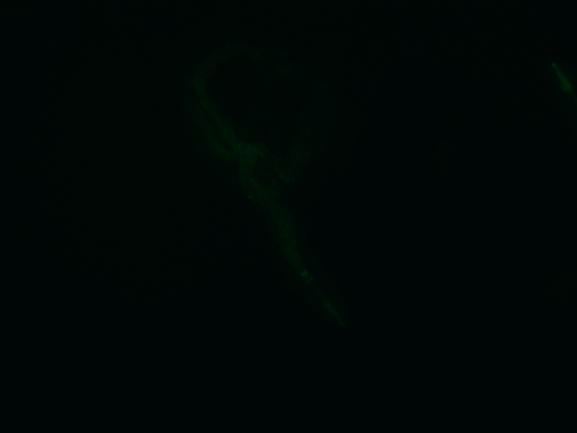

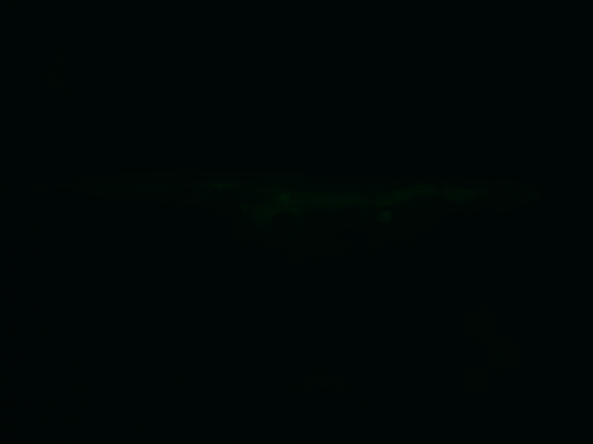

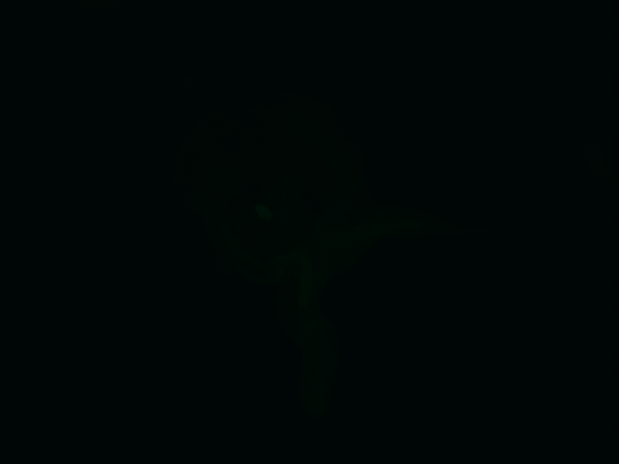

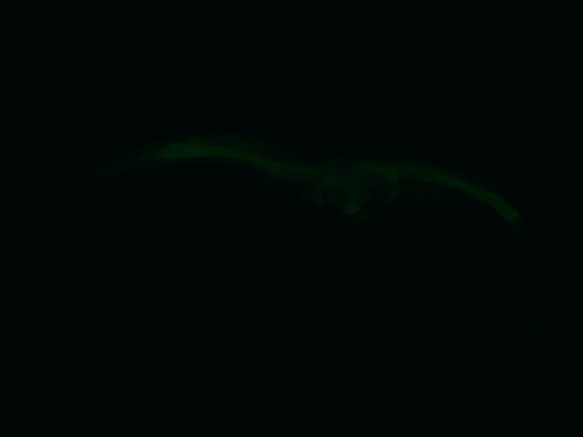

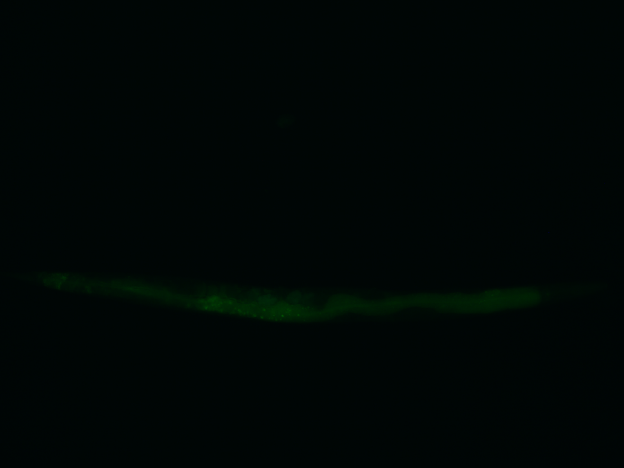

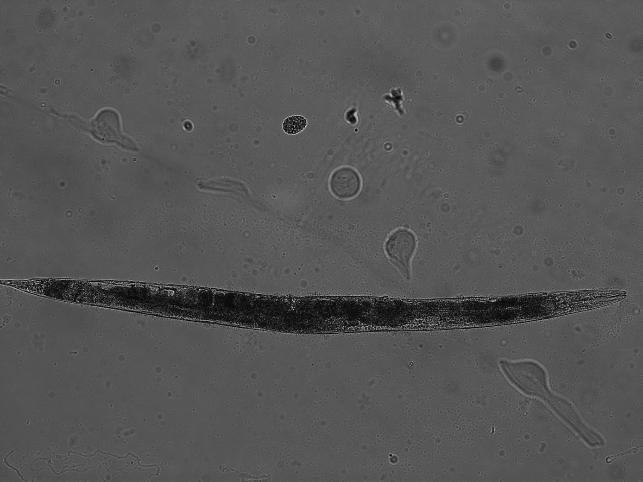


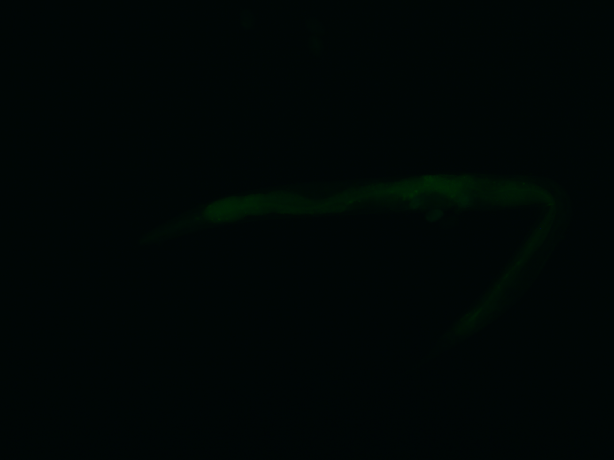


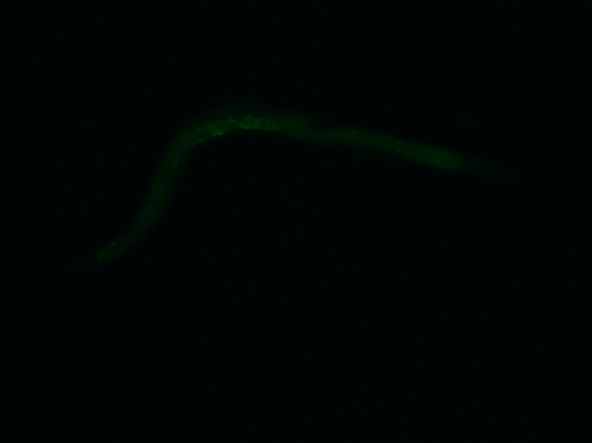

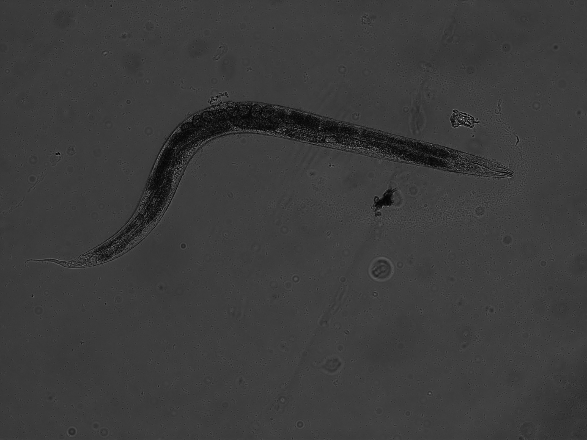

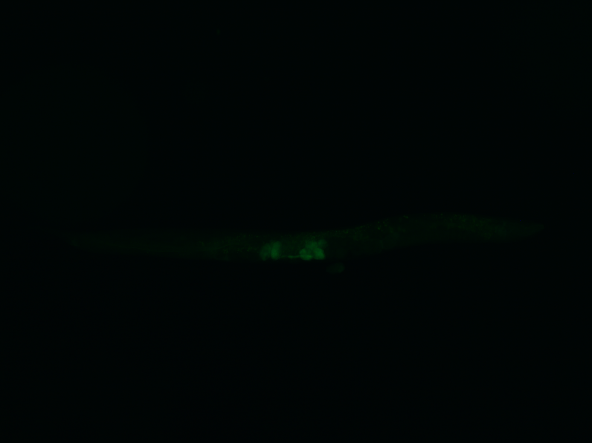

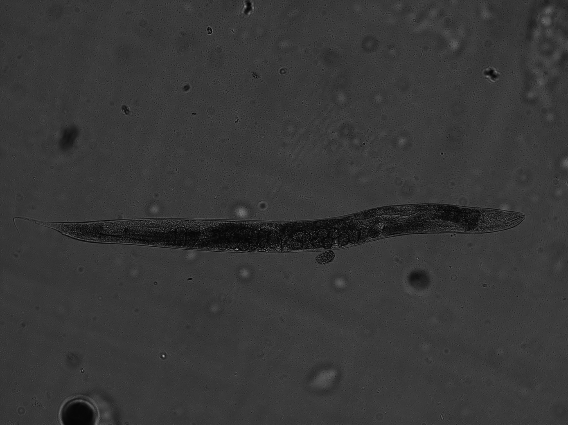

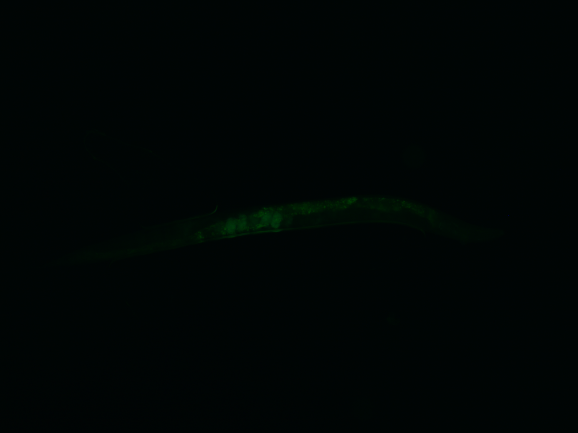

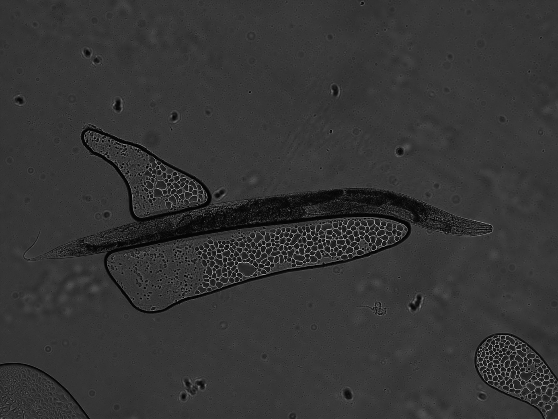


Fig. 3E BPA+BSHX


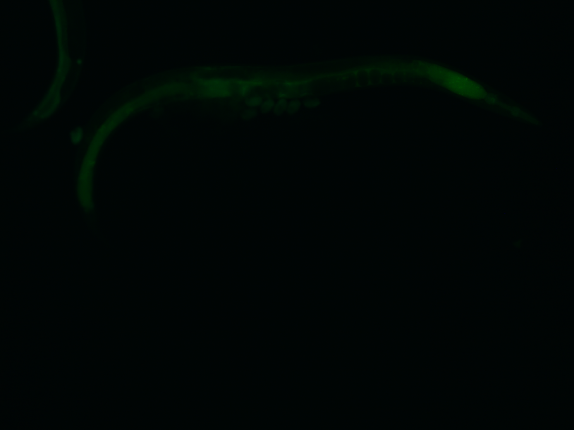


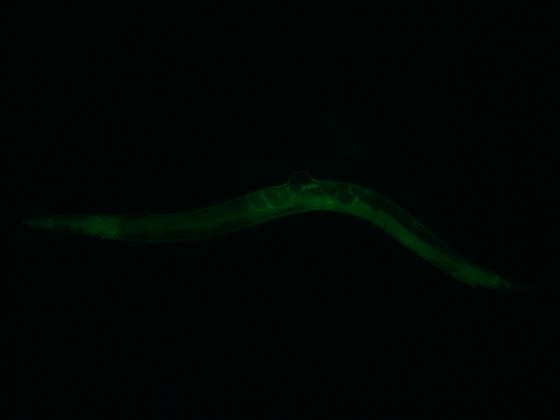

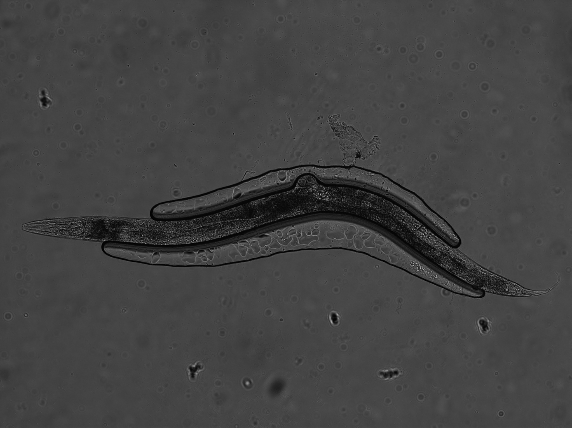

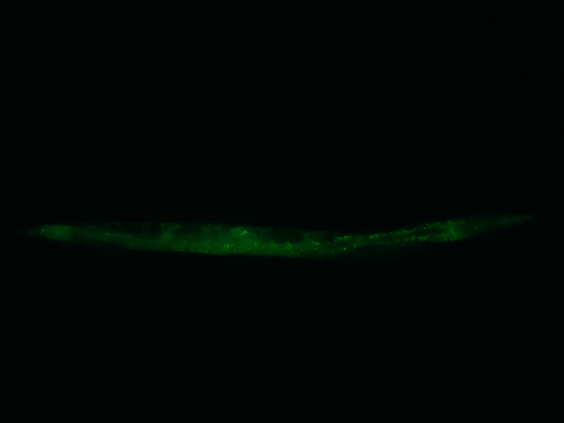

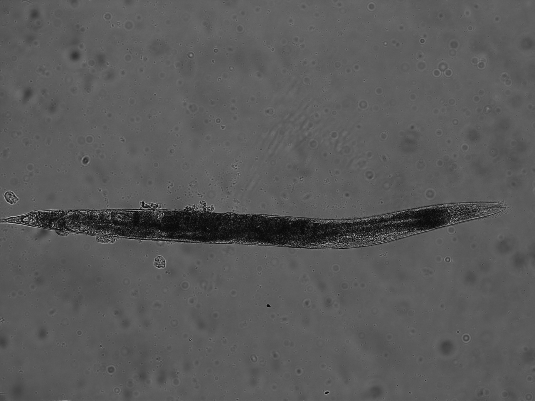

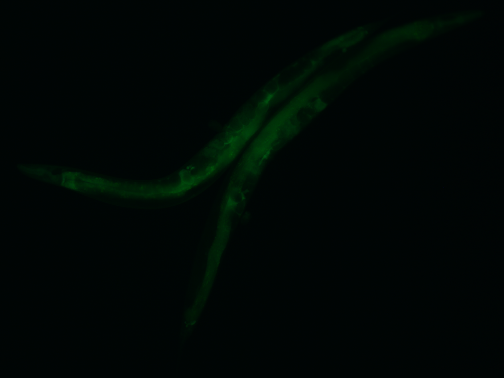

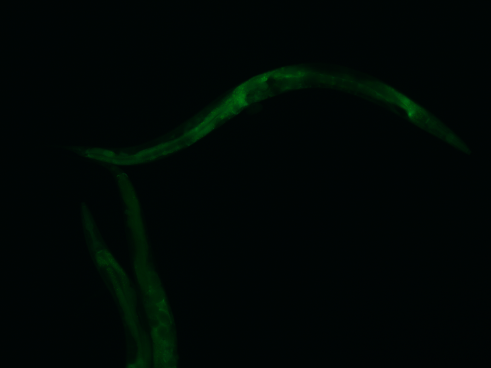

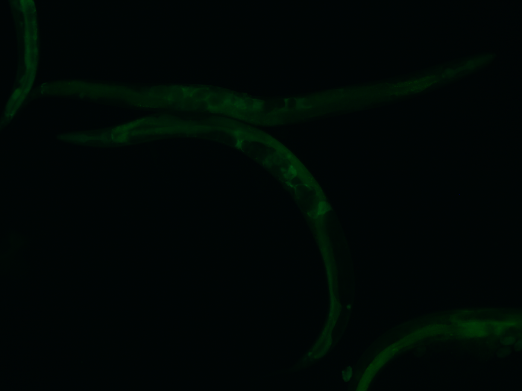


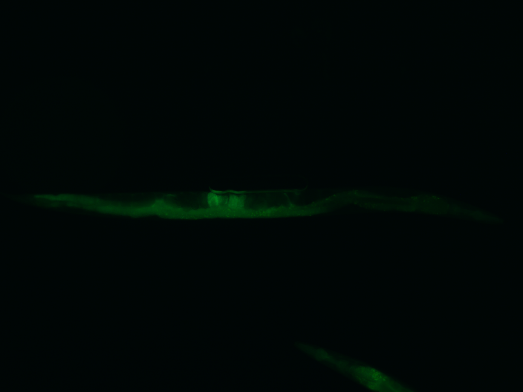

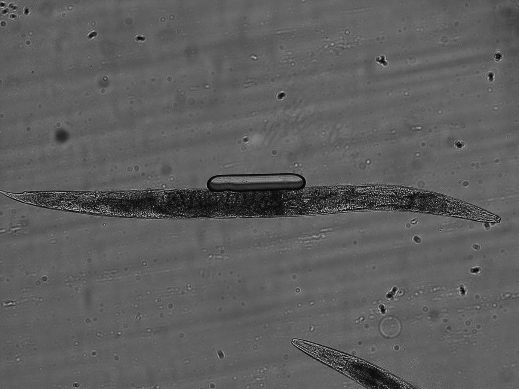

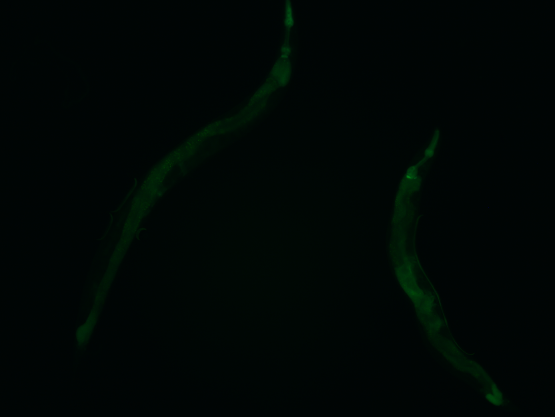


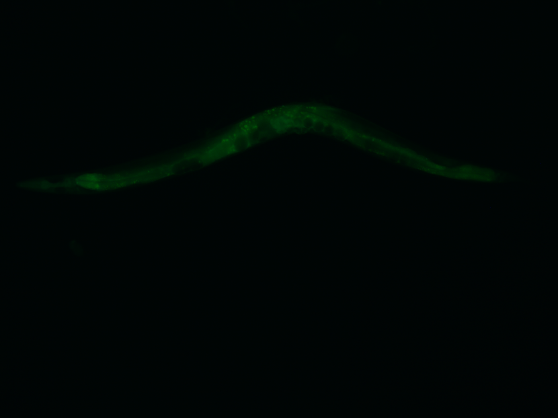

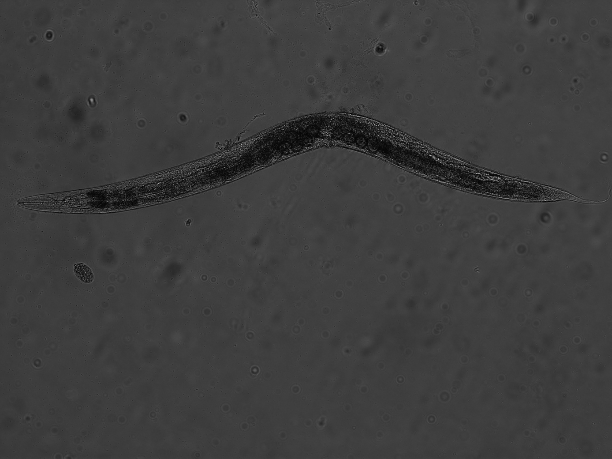

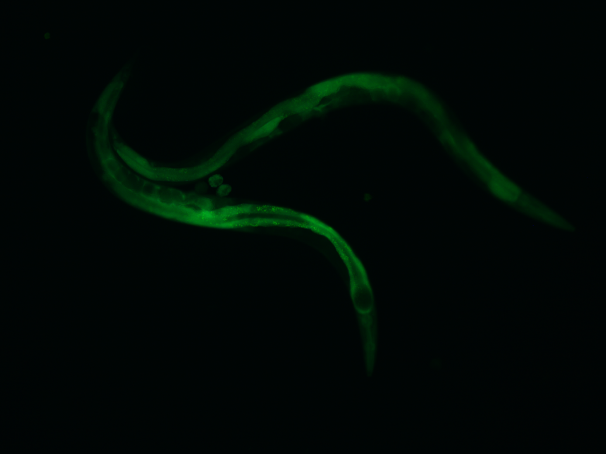


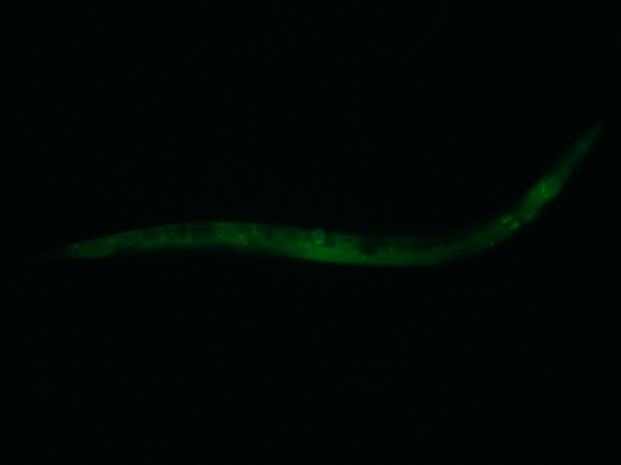

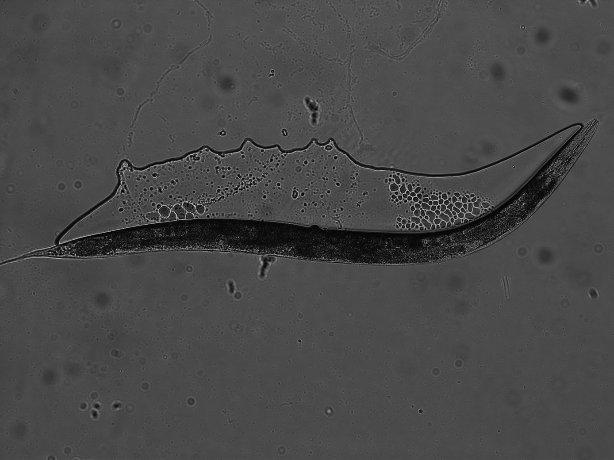

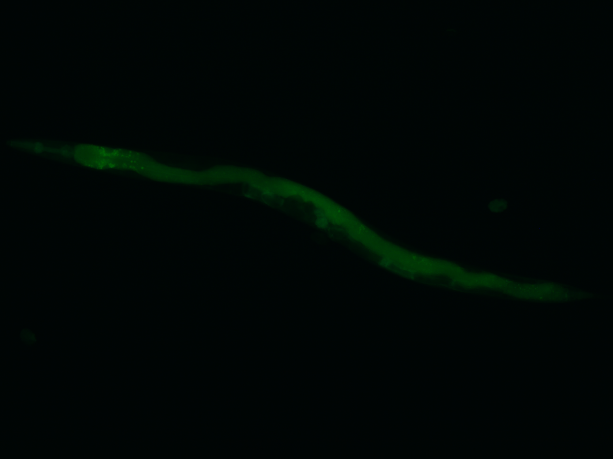

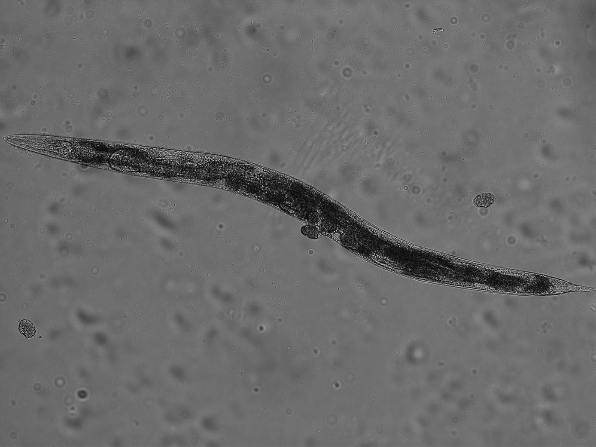

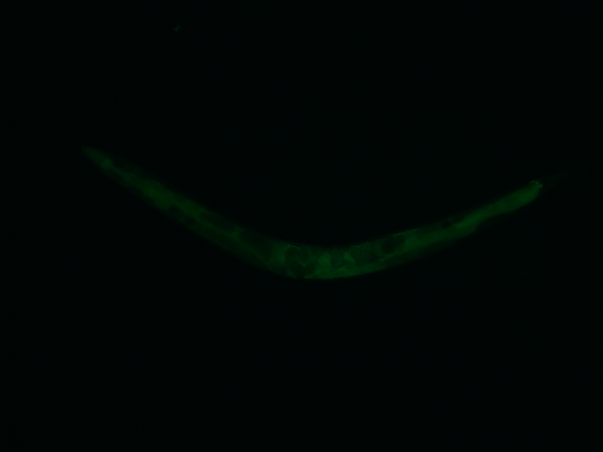

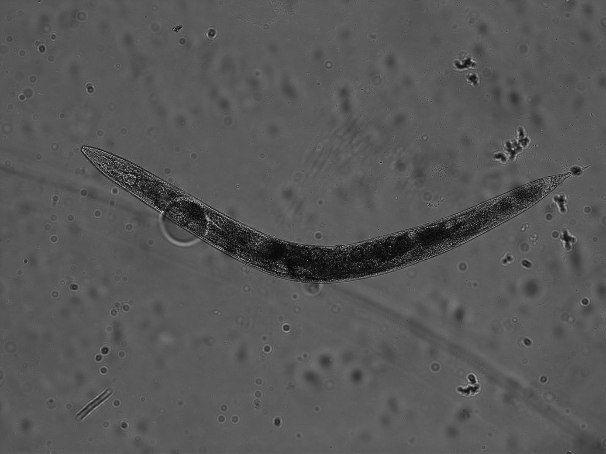

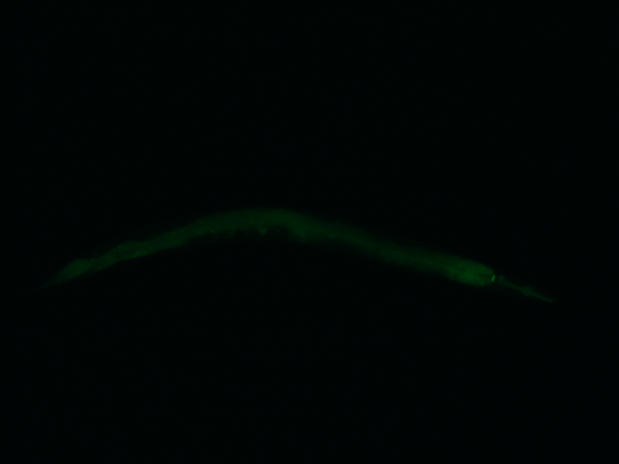

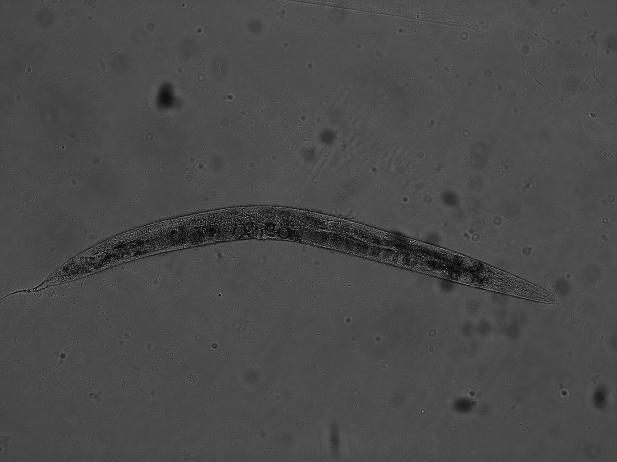

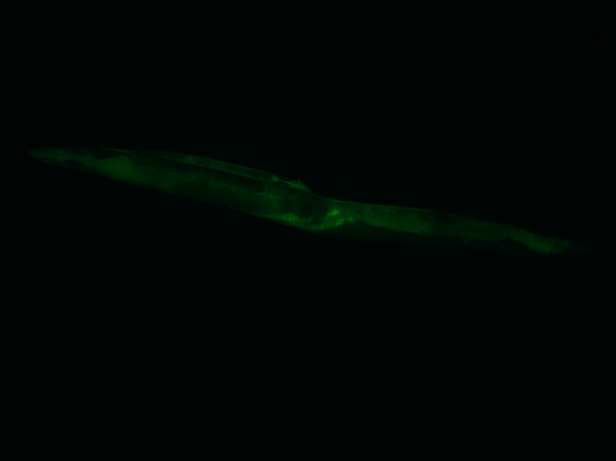

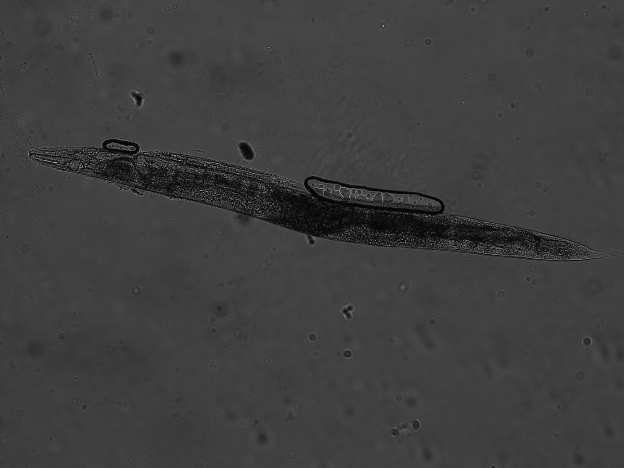

Supplement: Supplementary file 2 [file Table8.DOCX]

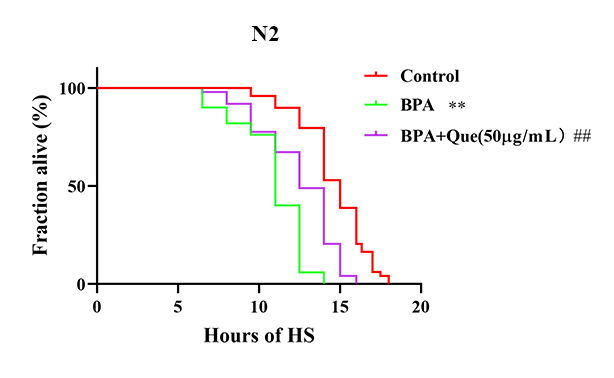

Supplement: Supplementary file 3 [file Image2.TIF]

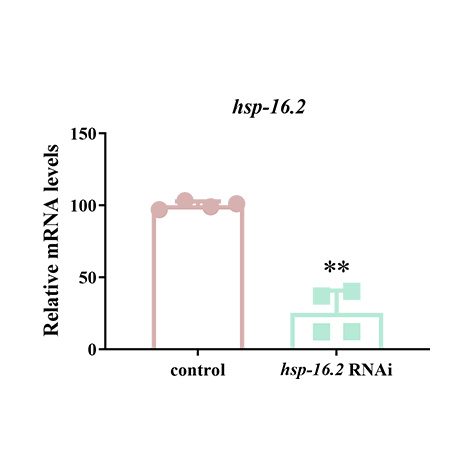

Supplement: Supplementary file 4 [file Image1.TIF]
