# Supplementary material for: BuShen HuoXue decoction improves fertility through intestinal hsp-16.2-mediated heat-shock signaling pathway in Caenorhabditis elegans
Source: Front Pharmacol. 2023 Jun 2;14:1210701. doi: 10.3389/fphar.2023.1210701 (PMC10272376; doi:10.3389/fphar.2023.1210701)
Supplement: Supplementary file 5 [file Table9.DOCX]

**Fig. 3 A**

| **Means and Medians for Survival Time** | | | | | | | | |
| --- | --- | --- | --- | --- | --- | --- | --- | --- |
| Group | Mean^a^ | | | | Median | | | |
|  | Estimate | Std. Error | 95% Confidence Interval | | Estimate | Std. Error | 95% Confidence Interval | |
|  |  |  | Lower Bound | Upper Bound |  |  | Lower Bound | Upper Bound |
| Control | 11.266 | .350 | 10.579 | 11.953 | 12.000 | .314 | 11.385 | 12.615 |
| BPA | 9.530 | .312 | 8.918 | 10.142 | 10.000 | .261 | 9.488 | 10.512 |
| BPA+BSHX | 10.790 | .259 | 10.283 | 11.297 | 11.000 | .254 | 10.503 | 11.497 |
| Overall | 10.517 | .186 | 10.153 | 10.881 | 11.000 | .128 | 10.749 | 11.251 |
| a. Estimation is limited to the largest survival time if it is censored. | | | | | | | | |

| **Pairwise Comparisons** | | | | | | | |
| --- | --- | --- | --- | --- | --- | --- | --- |
|  | Group | Control | | BPA | | BPA+BSHX | |
|  |  | Chi-Square | Sig. | Chi-Square | Sig. | Chi-Square | Sig. |
| Log Rank (Mantel-Cox) | Control |  |  | 22.107 | .000 | 3.360 | .067 |
|  | BPA | 22.107 | .000 |  |  | 9.463 | .002 |
|  | BPA+BSHX | 3.360 | .067 | 9.463 | .002 |  |  |

**Fig. 3 B**

| **Means and Medians for Survival Time** | | | | | | | | |
| --- | --- | --- | --- | --- | --- | --- | --- | --- |
| Group | Mean^a^ | | | | Median | | | |
|  | Estimate | Std. Error | 95% Confidence Interval | | Estimate | Std. Error | 95% Confidence Interval | |
|  |  |  | Lower Bound | Upper Bound |  |  | Lower Bound | Upper Bound |
| Control | 5.540 | .293 | 4.965 | 6.115 | 5.000 | .421 | 4.176 | 5.824 |
| BSHX | 5.440 | .261 | 4.929 | 5.951 | 5.000 | .319 | 4.375 | 5.625 |
| Overall | 5.490 | .195 | 5.107 | 5.873 | 5.000 | .260 | 4.491 | 5.509 |
| a. Estimation is limited to the largest survival time if it is censored. | | | | | | | | |

| **Pairwise Comparisons** | | | | | |
| --- | --- | --- | --- | --- | --- |
|  | Group | Control | | BSHX | |
|  |  | Chi-Square | Sig. | Chi-Square | Sig. |
| Log Rank (Mantel-Cox) | Control |  |  | .067 | .796 |
|  | BSHX | .067 | .796 |  |  |

**Fig. 3 C**

| **Means and Medians for Survival Time** | | | | | | | | |
| --- | --- | --- | --- | --- | --- | --- | --- | --- |
| Group | Mean^a^ | | | | Median | | | |
|  | Estimate | Std. Error | 95% Confidence Interval | | Estimate | Std. Error | 95% Confidence Interval | |
|  |  |  | Lower Bound | Upper Bound |  |  | Lower Bound | Upper Bound |
| Control | 4.060 | .204 | 3.659 | 4.461 | 3.500 | .264 | 2.983 | 4.017 |
| BSHX | 4.280 | .212 | 3.864 | 4.696 | 3.500 | .292 | 2.927 | 4.073 |
| Overall | 4.170 | .147 | 3.882 | 4.458 | 3.500 | .197 | 3.114 | 3.886 |
| a. Estimation is limited to the largest survival time if it is censored. | | | | | | | | |

| **Pairwise Comparisons** | | | | | |
| --- | --- | --- | --- | --- | --- |
|  | Group | Control | | BSHX | |
|  |  | Chi-Square | Sig. | Chi-Square | Sig. |
| Log Rank (Mantel-Cox) | Control |  |  | .566 | .452 |
|  | BSHX | .566 | .452 |  |  |

**Fig. 3 D**

1. *Hsp-16.1*

| **Tests of Normality** | | | | | | | |
| --- | --- | --- | --- | --- | --- | --- | --- |
|  | Group | Kolmogorov-Smirnov^a^ | | | Shapiro-Wilk | | |
|  |  | Statistic | df | Sig. | Statistic | df | Sig. |
| *Hsp-16.1* | Control | .214 | 3 | . | .989 | 3 | .803 |
|  | BPA | .322 | 3 | . | .880 | 3 | .325 |
|  | BPA+BSHX | .239 | 3 | . | .975 | 3 | .697 |
| a. Lilliefors Significance Correction | | | | | | | |

- 1. Control VS BPA

| **Group Statistics** | | | | | |
| --- | --- | --- | --- | --- | --- |
|  | Group | N | Mean | Std. Deviation | Std. Error Mean |
| *Hsp-16.1* | Control | 3 | 100.7467 | 7.67089 | 4.42879 |
|  | BPA | 3 | 19.5533 | 2.21152 | 1.27682 |

| **Independent Samples Test** | | | | | | | | | | |
| --- | --- | --- | --- | --- | --- | --- | --- | --- | --- | --- |
|  | | Levene's Test for Equality of Variances | | t-test for Equality of Means | | | | | | |
|  |  | F | Sig. | t | df | Sig. (2-tailed) | Mean Difference | Std. Error Difference | 95% Confidence Interval of the Difference | |
|  |  |  |  |  |  |  |  |  | Lower | Upper |
| *Hsp-16.1* | Equal variances assumed | 2.599 | .182 | 17.616 | 4 | .000 | 81.19333 | 4.60917 | 68.39622 | 93.99044 |
|  | Equal variances not assumed |  |  | 17.616 | 2.330 | .002 | 81.19333 | 4.60917 | 63.82550 | 98.56116 |

- 1. BPA VS BPA+BSHX

| **Group Statistics** | | | | | |
| --- | --- | --- | --- | --- | --- |
|  | Group | N | Mean | Std. Deviation | Std. Error Mean |
| *Hsp-16.1* | BPA | 3 | 19.5533 | 2.21152 | 1.27682 |
|  | BPA+BSHX | 3 | 38.6667 | .84056 | .48529 |

| **Independent Samples Test** | | | | | | | | | | |
| --- | --- | --- | --- | --- | --- | --- | --- | --- | --- | --- |
|  | | Levene's Test for Equality of Variances | | t-test for Equality of Means | | | | | | |
|  |  | F | Sig. | t | df | Sig. (2-tailed) | Mean Difference | Std. Error Difference | 95% Confidence Interval of the Difference | |
|  |  |  |  |  |  |  |  |  | Lower | Upper |
| *Hsp-16.1* | Equal variances assumed | 4.179 | .110 | -13.993 | 4 | .000 | -19.11333 | 1.36594 | -22.90579 | -15.32088 |
|  | Equal variances not assumed |  |  | -13.993 | 2.566 | .002 | -19.11333 | 1.36594 | -23.90736 | -14.31931 |

1. *Hsp-16.2*

| **Tests of Normality** | | | | | | | |
| --- | --- | --- | --- | --- | --- | --- | --- |
|  | Group | Kolmogorov-Smirnov^a^ | | | Shapiro-Wilk | | |
|  |  | Statistic | df | Sig. | Statistic | df | Sig. |
| *Hsp-16.2* | Control | .291 | 3 | . | .924 | 3 | .467 |
|  | BPA | .270 | 3 | . | .948 | 3 | .561 |
|  | BPA+BSHX | .367 | 3 | . | .793 | 3 | .098 |
| a. Lilliefors Significance Correction | | | | | | | |

- 1. Control VS BPA

| **Group Statistics** | | | | | |
| --- | --- | --- | --- | --- | --- |
|  | Group | N | Mean | Std. Deviation | Std. Error Mean |
| *Hsp-16.2* | Control | 3 | 100.8100 | 1.44596 | .83483 |
|  | BPA | 3 | 13.0900 | 1.33000 | .76788 |

| **Independent Samples Test** | | | | | | | | | | |
| --- | --- | --- | --- | --- | --- | --- | --- | --- | --- | --- |
|  | | Levene's Test for Equality of Variances | | t-test for Equality of Means | | | | | | |
|  |  | F | Sig. | t | df | Sig. (2-tailed) | Mean Difference | Std. Error Difference | 95% Confidence Interval of the Difference | |
|  |  |  |  |  |  |  |  |  | Lower | Upper |
| *Hsp-16.2* | Equal variances assumed | .045 | .843 | 77.336 | 4 | .000 | 87.72000 | 1.13427 | 84.57076 | 90.86924 |
|  | Equal variances not assumed |  |  | 77.336 | 3.972 | .000 | 87.72000 | 1.13427 | 84.56211 | 90.87789 |

2.2 BPA VS BPA+BSHX

| **Group Statistics** | | | | | |
| --- | --- | --- | --- | --- | --- |
|  | Group | N | Mean | Std. Deviation | Std. Error Mean |
| *Hsp-16.2* | BPA | 3 | 13.0900 | 1.33000 | .76788 |
|  | BPA+BSHX | 3 | 25.5033 | 1.17356 | .67755 |

| **Independent Samples Test** | | | | | | | | | | |
| --- | --- | --- | --- | --- | --- | --- | --- | --- | --- | --- |
|  | | Levene's Test for Equality of Variances | | t-test for Equality of Means | | | | | | |
|  |  | F | Sig. | t | df | Sig. (2-tailed) | Mean Difference | Std. Error Difference | 95% Confidence Interval of the Difference | |
|  |  |  |  |  |  |  |  |  | Lower | Upper |
| *Hsp-16.2* | Equal variances assumed | .037 | .856 | -12.122 | 4 | .000 | -12.41333 | 1.02407 | -15.25660 | -9.57007 |
|  | Equal variances not assumed |  |  | -12.122 | 3.939 | .000 | -12.41333 | 1.02407 | -15.27406 | -9.55261 |

1. *Hsp-16.41*

| **Tests of Normality** | | | | | | | |
| --- | --- | --- | --- | --- | --- | --- | --- |
|  | Group | Kolmogorov-Smirnov^a^ | | | Shapiro-Wilk | | |
|  |  | Statistic | df | Sig. | Statistic | df | Sig. |
| *Hsp-16.41* | Control | .212 | 3 | . | .990 | 3 | .810 |
|  | BPA | .295 | 3 | . | .920 | 3 | .452 |
|  | BPA+BSHX | .176 | 3 | . | 1.000 | 3 | .985 |
| a. Lilliefors Significance Correction | | | | | | | |

- 1. Control VS BPA

| **Group Statistics** | | | | | |
| --- | --- | --- | --- | --- | --- |
|  | Group | N | Mean | Std. Deviation | Std. Error Mean |
| *Hsp-16.41* | Control | 3 | 100.1700 | 2.44207 | 1.40993 |
|  | BPA | 3 | 16.8567 | .23459 | .13544 |

| **Independent Samples Test** | | | | | | | | | | |
| --- | --- | --- | --- | --- | --- | --- | --- | --- | --- | --- |
|  | | Levene's Test for Equality of Variances | | t-test for Equality of Means | | | | | | |
|  |  | F | Sig. | t | df | Sig. (2-tailed) | Mean Difference | Std. Error Difference | 95% Confidence Interval of the Difference | |
|  |  |  |  |  |  |  |  |  | Lower | Upper |
| *Hsp-16.41* | Equal variances assumed | 4.521 | .101 | 58.820 | 4 | .000 | 83.31333 | 1.41642 | 79.38072 | 87.24594 |
|  | Equal variances not assumed |  |  | 58.820 | 2.037 | .000 | 83.31333 | 1.41642 | 77.32358 | 89.30308 |

- 1. BPA VS BPA+BSHX

| **Group Statistics** | | | | | |
| --- | --- | --- | --- | --- | --- |
|  | Group | N | Mean | Std. Deviation | Std. Error Mean |
| *Hsp-16.41* | BPA | 3 | 16.8567 | .23459 | .13544 |
|  | BPA+BSHX | 3 | 32.3200 | 1.11503 | .64376 |

| **Independent Samples Test** | | | | | | | | | | |
| --- | --- | --- | --- | --- | --- | --- | --- | --- | --- | --- |
|  | | Levene's Test for Equality of Variances | | t-test for Equality of Means | | | | | | |
|  |  | F | Sig. | t | df | Sig. (2-tailed) | Mean Difference | Std. Error Difference | 95% Confidence Interval of the Difference | |
|  |  |  |  |  |  |  |  |  | Lower | Upper |
| *Hsp-16.41* | Equal variances assumed | 2.353 | .200 | -23.506 | 4 | .000 | -15.46333 | .65786 | -17.28984 | -13.63683 |
|  | Equal variances not assumed |  |  | -23.506 | 2.177 | .001 | -15.46333 | .65786 | -18.08468 | -12.84199 |

1. *Hsp-16.48*

| **Tests of Normality** | | | | | | | |
| --- | --- | --- | --- | --- | --- | --- | --- |
|  | Group | Kolmogorov-Smirnov^a^ | | | Shapiro-Wilk | | |
|  |  | Statistic | df | Sig. | Statistic | df | Sig. |
| *Hsp-16.48* | Control | .353 | 3 | . | .824 | 3 | .172 |
|  | BPA | .181 | 3 | . | .999 | 3 | .941 |
|  | BPA+BSHX | .233 | 3 | . | .979 | 3 | .722 |
| a. Lilliefors Significance Correction | | | | | | | |

- 1. Control VS BPA

| **Group Statistics** | | | | | |
| --- | --- | --- | --- | --- | --- |
|  | Group | N | Mean | Std. Deviation | Std. Error Mean |
| *Hsp-16.48* | Control | 3 | 99.2167 | 3.43769 | 1.98475 |
|  | BPA | 3 | 22.0800 | 1.67081 | .96464 |

| **Independent Samples Test** | | | | | | | | | | |
| --- | --- | --- | --- | --- | --- | --- | --- | --- | --- | --- |
|  | | Levene's Test for Equality of Variances | | t-test for Equality of Means | | | | | | |
|  |  | F | Sig. | t | df | Sig. (2-tailed) | Mean Difference | Std. Error Difference | 95% Confidence Interval of the Difference | |
|  |  |  |  |  |  |  |  |  | Lower | Upper |
| *Hsp-16.48* | Equal variances assumed | 2.991 | .159 | 34.955 | 4 | .000 | 77.13667 | 2.20676 | 71.00973 | 83.26361 |
|  | Equal variances not assumed |  |  | 34.955 | 2.895 | .000 | 77.13667 | 2.20676 | 69.96738 | 84.30595 |

- 1. BPA VS BPA+BSHX

| **Group Statistics** | | | | | |
| --- | --- | --- | --- | --- | --- |
|  | Group | N | Mean | Std. Deviation | Std. Error Mean |
| *Hsp-16.48* | BPA | 3 | 22.0800 | 1.67081 | .96464 |
|  | BPA+BSHX | 3 | 36.6567 | .33858 | .19548 |

| **Independent Samples Test** | | | | | | | | | | |
| --- | --- | --- | --- | --- | --- | --- | --- | --- | --- | --- |
|  | | Levene's Test for Equality of Variances | | t-test for Equality of Means | | | | | | |
|  |  | F | Sig. | t | df | Sig. (2-tailed) | Mean Difference | Std. Error Difference | 95% Confidence Interval of the Difference | |
|  |  |  |  |  |  |  |  |  | Lower | Upper |
| *Hsp-16.48* | Equal variances assumed | 2.672 | .177 | -14.810 | 4 | .000 | -14.57667 | .98425 | -17.30938 | -11.84396 |
|  | Equal variances not assumed |  |  | -14.810 | 2.164 | .003 | -14.57667 | .98425 | -18.51841 | -10.63492 |

**Fig. 3 E**

| **Tests of Normality** | | | | | | | |
| --- | --- | --- | --- | --- | --- | --- | --- |
|  | Group | Kolmogorov-Smirnov^a^ | | | Shapiro-Wilk | | |
|  |  | Statistic | df | Sig. | Statistic | df | Sig. |
| Relative fluorescence intensity | Control | .205 | 13 | .138 | .949 | 13 | .584 |
|  | BPA | .126 | 17 | .200^*^ | .964 | 17 | .714 |
|  | BPA+BSHX | .137 | 15 | .200^*^ | .905 | 15 | .112 |
| *. This is a lower bound of the true significance. | | | | | | | |
| a. Lilliefors Significance Correction | | | | | | | |

1. Control VS BPA

| **Group Statistics** | | | | | |
| --- | --- | --- | --- | --- | --- |
|  | Group | N | Mean | Std. Deviation | Std. Error Mean |
| Relative fluorescence intensity | Control | 13 | 16.5447 | 3.59619 | .99740 |
|  | BPA | 17 | 7.3965 | 1.41866 | .34408 |

| **Independent Samples Test** | | | | | | | | | | |
| --- | --- | --- | --- | --- | --- | --- | --- | --- | --- | --- |
|  | | Levene's Test for Equality of Variances | | t-test for Equality of Means | | | | | | |
|  |  | F | Sig. | t | df | Sig. (2-tailed) | Mean Difference | Std. Error Difference | 95% Confidence Interval of the Difference | |
|  |  |  |  |  |  |  |  |  | Lower | Upper |
| Relative fluorescence intensity | Equal variances assumed | 13.998 | .001 | 9.598 | 28 | .000 | 9.14816 | .95315 | 7.19572 | 11.10060 |
|  | Equal variances not assumed |  |  | 8.671 | 14.868 | .000 | 9.14816 | 1.05508 | 6.89757 | 11.39876 |

# BPA VS BPA+BSHX

| **Group Statistics** | | | | | |
| --- | --- | --- | --- | --- | --- |
|  | Group | N | Mean | Std. Deviation | Std. Error Mean |
| Relative fluorescence intensity | BPA | 17 | 7.3965 | 1.41866 | .34408 |
|  | BPA+BSHX | 15 | 12.1819 | 3.17213 | .81904 |

| **Independent Samples Test** | | | | | | | | | | |
| --- | --- | --- | --- | --- | --- | --- | --- | --- | --- | --- |
|  | | Levene's Test for Equality of Variances | | t-test for Equality of Means | | | | | | |
|  |  | F | Sig. | t | df | Sig. (2-tailed) | Mean Difference | Std. Error Difference | 95% Confidence Interval of the Difference | |
|  |  |  |  |  |  |  |  |  | Lower | Upper |
| Relative fluorescence intensity | Equal variances assumed | 6.564 | .016 | -5.624 | 30 | .000 | -4.78540 | .85087 | -6.52310 | -3.04770 |
|  | Equal variances not assumed |  |  | -5.387 | 18.863 | .000 | -4.78540 | .88838 | -6.64571 | -2.92510 |
