# Supplementary material for: BuShen HuoXue decoction improves fertility through intestinal hsp-16.2-mediated heat-shock signaling pathway in Caenorhabditis elegans
Source: Front Pharmacol. 2023 Jun 2;14:1210701. doi: 10.3389/fphar.2023.1210701 (PMC10272376; doi:10.3389/fphar.2023.1210701)
Supplement: Supplementary file 6 [file Table1.DOC]

| **RNAi primer** | | |
| --- | --- | --- |
| Gene name | Forward | Reverse |
| *hsp-16.2* | ATAGGTACCAGATTCGAAGCAACTGCA | CTACTGCAGAATTTGGATGGTCGTACG |
| **qPCR primer** | | |
| Gene name | Forward | Reverse |
| *act-3* | ATCCGTAAGGACTTGTACGCCAAC | GGGCGATGATCTTGATCTTCATGG |
| *hsp-16.1* | CAATGTCTCGCAGTTCAAGC | GCAACTGCACCAACATCAAC |
| *hsp-16,2* | TTGCCATCAATCTCAACGTCTC | CTTTCTTTGGCGCTTCAATCGA |
| *hsp-16.41* | TTTTCGGTTCAACTCGATGTCTC | TCTTTGGAGCCTCAATTTGGAG |
| *hsp-16.48* | CATGCTCCGTTCTCCATTTT | TGGTTTGAAATGAGAAACATCG |
| *clc-2* | GATGAAGTTTCCGGTTTTCG | ACATCCGAAAGCAATTACCG |
| *egl-8* | GCCAGTGGCTGTGGATAAAT | TTGCTGCTGTTTTTGAATGC |
| *ifb-2* | TGGAAATGACATCGGACAGA | ATAGCTCCTCCCCAAGTGGT |
| *dlg-1* | CAATGAGGAGCTACGCACAA | TTCGAGTCGCTCAATCACAC |
| *act-5* | CCATCCAACGTCTCGATCTT | ATCTCTCGTTTCCGATGGTG |
| *gtl-1* | AGGAAAACGTGGATCTGTGG | GGTGATCGGAGCCTTGTAAA |
| *nfm-1* | TGGATCTTTGCATTGGAACA | TCCATTTGCTGCCTCTCTTT |
| *let-413* | TCACCAAAGTCACACCTGGA | GGCTTGGCTCAGATGTTCTC |
| *par-6* | GAATGGCGTCGTTTCTCAAT | CGAACGATTTTCGGAGGTTA |
| *abts-4* | TTCCTACCCAGCAAATACCG | TTCACAAATATGGCGTTGGA |
| *pkc-3* | GCTTCCGTGTCAAGGAGAAG | ACGACAATCCACACATCGAA |
| *mtm-6* | TACCGCACACGTTGCTCTAC | GTGCCACGTGGGATTCTAGT |
| *par-3* | AGTGCGATGCGAAACTTCTTTG | TCCACGTCGTTGTGATTGATGT |
| *erm-1* | TCTTGGTGTCGATGCTCTTG | GGAGTCGTGGAGCGTAGAAG |
