# Supplementary material for: BuShen HuoXue decoction improves fertility through intestinal hsp-16.2-mediated heat-shock signaling pathway in Caenorhabditis elegans
Source: Front Pharmacol. 2023 Jun 2;14:1210701. doi: 10.3389/fphar.2023.1210701 (PMC10272376; doi:10.3389/fphar.2023.1210701)
Supplement: Supplementary file 7 [file Table2.DOCX]

**Supplementary Table S2. Qualitative information of the components identified**

| **Standard/Name** | **Formula** | **Ionization mode** | ***m/z* predicted** | **R.T. (min)** |
| --- | --- | --- | --- | --- |
| Catalpol | C_15_H_22_O_10_ | [M+COOH]^-^ | 407.1195 | 2.196 |
| Loganin | C_17_H_26_O_10_ | [M+COOH]^-^ | 435.1508 | 13.009 |
| Paeoniflorin | C_23_H_28_O_11_ | [M+COOH]^-^ | 525.1614 | 14.608 |
| Verbascoside | C_29_H_36_O_15_ | [M-H]^-^ | 623.1981 | 17.883 |
| Salvianolic acid B | C_36_H_30_O_16_ | [M-H]^-^ | 717.1461 | 20.777 |
| Quercetin | C_15_H_10_O_7_ | [M-H]^-^ | 301.0354 | 21.996 |
| Asperosaponin | C_47_H_76_O_18_ | [M-H]^-^ | 927.4959 | 22.148 |
