# Supplementary material for: BuShen HuoXue decoction improves fertility through intestinal hsp-16.2-mediated heat-shock signaling pathway in Caenorhabditis elegans
Source: Front Pharmacol. 2023 Jun 2;14:1210701. doi: 10.3389/fphar.2023.1210701 (PMC10272376; doi:10.3389/fphar.2023.1210701)
Supplement: Supplementary file 8 [file Table5.DOCX]

Fig. 2E Control


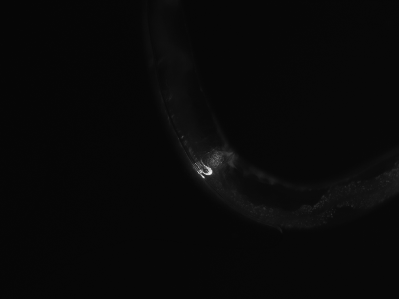

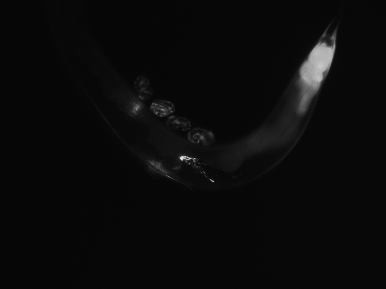

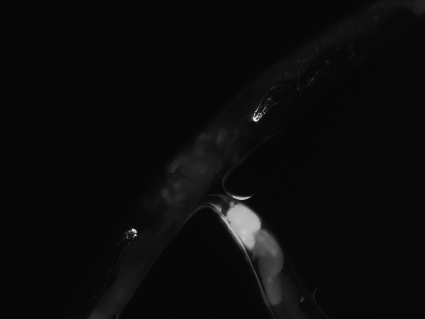


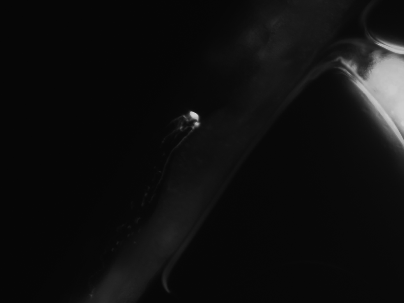

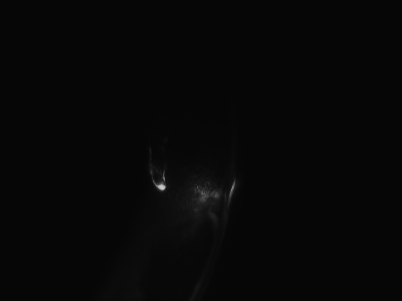

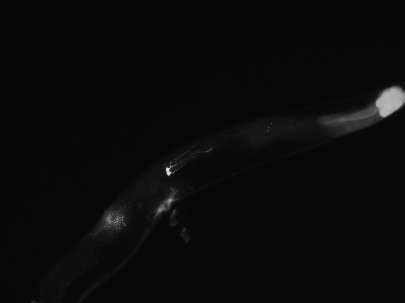


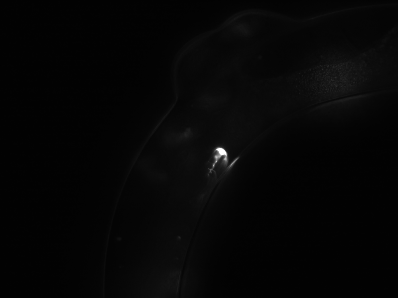

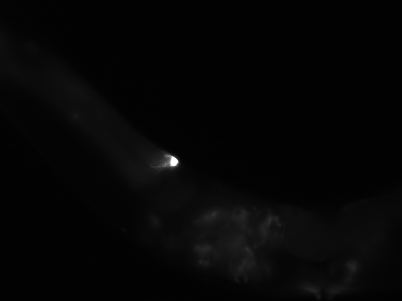

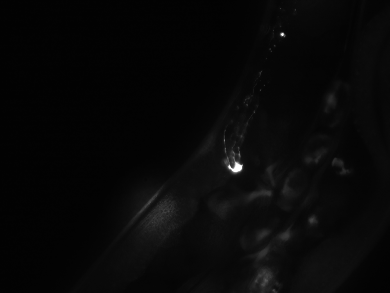


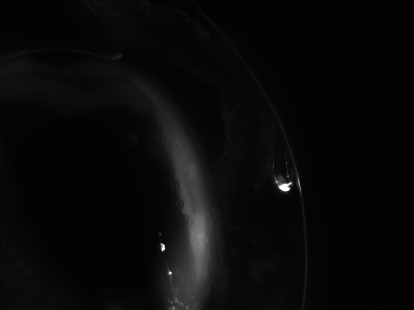

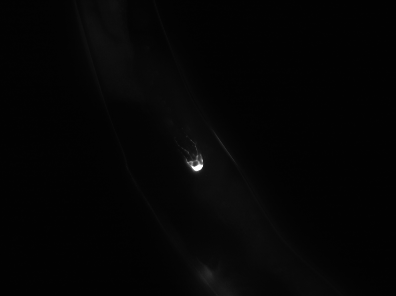

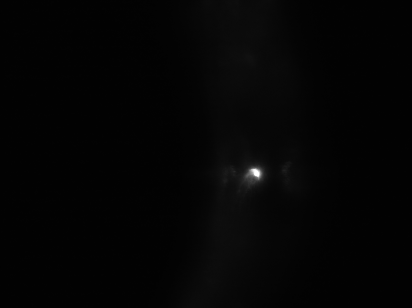


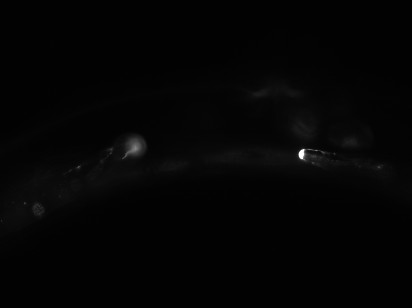

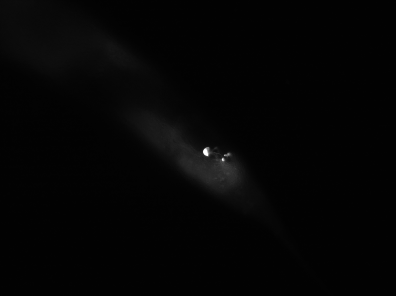

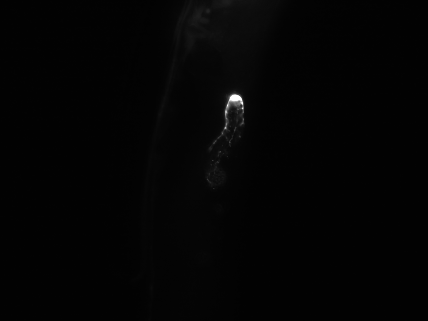


Fig. 2E BPA


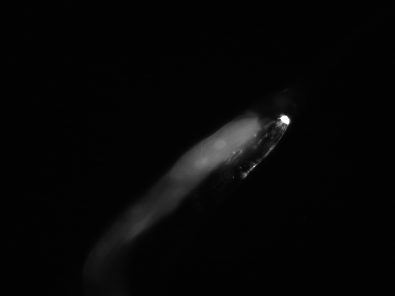

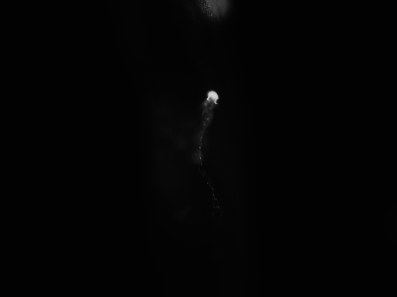

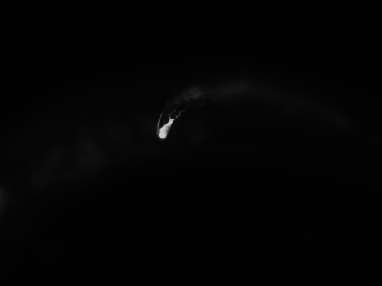


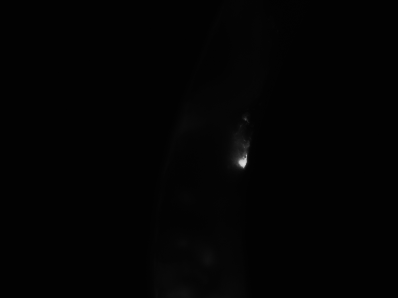

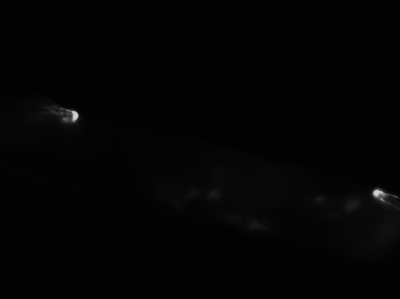

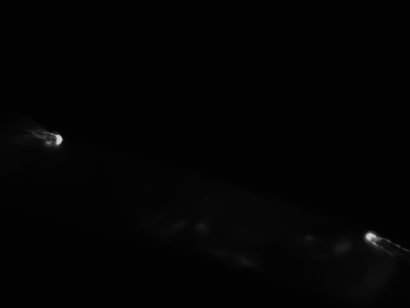


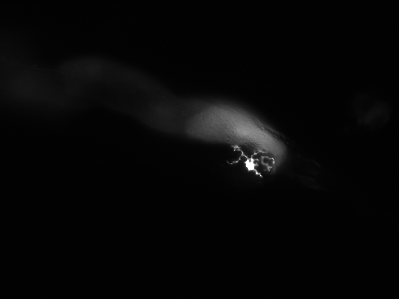

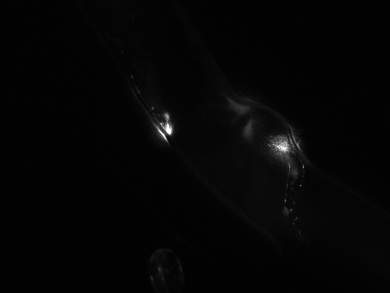

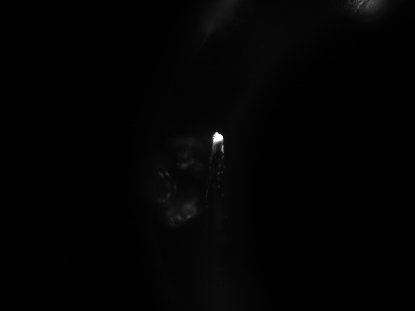


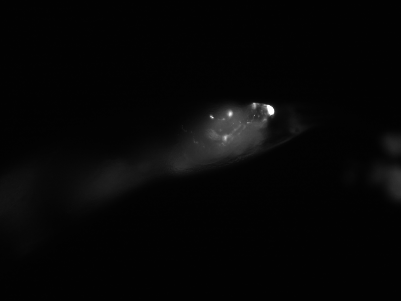

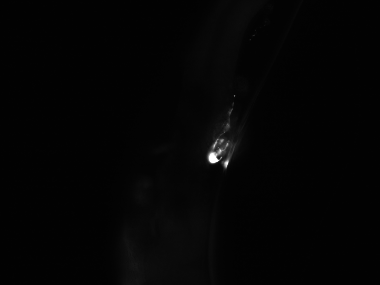

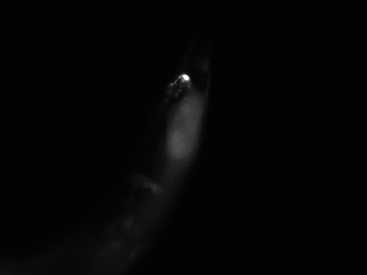


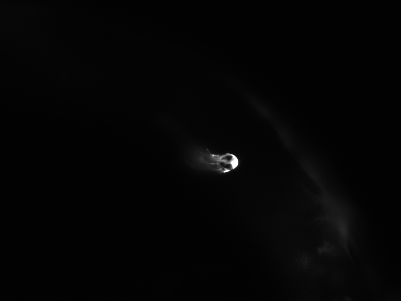

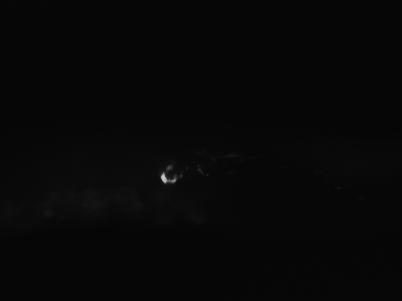

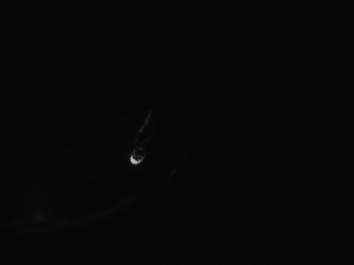


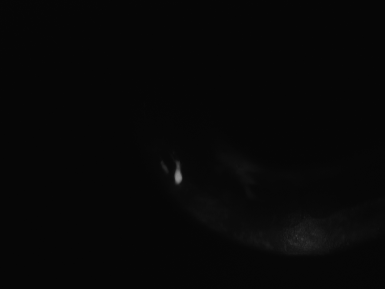

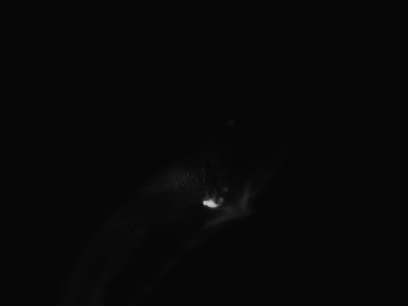

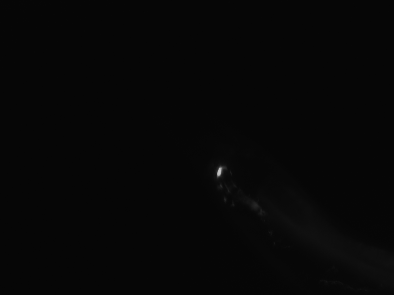


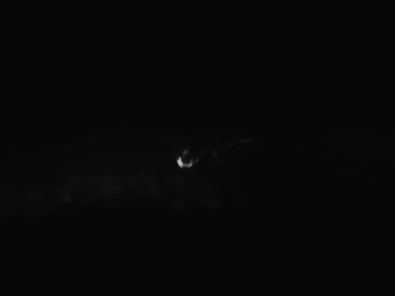

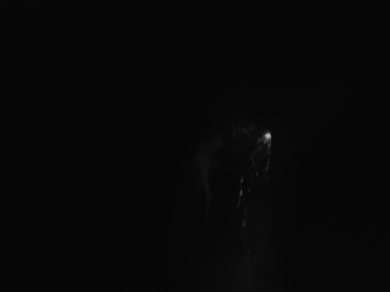

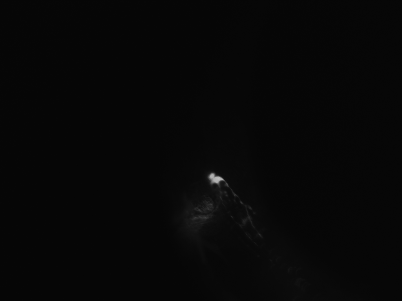


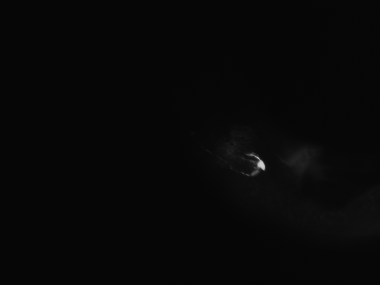

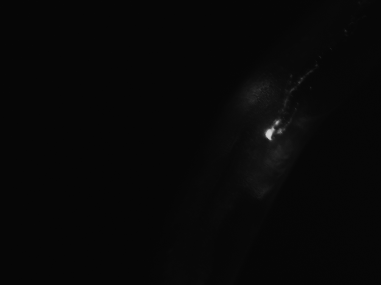

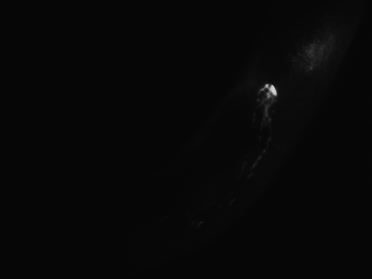


Fig. 2E BPA+BSHX


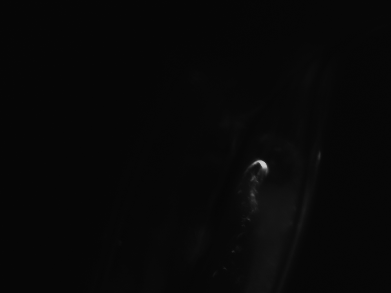

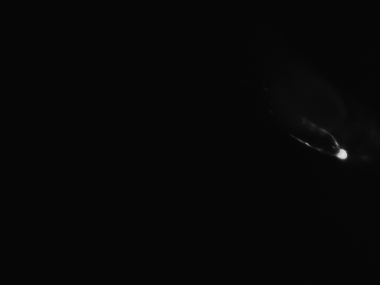

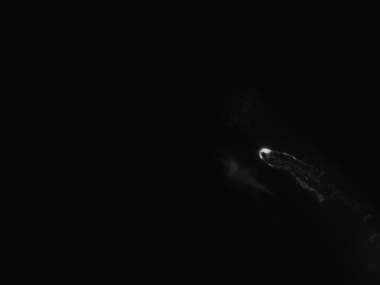

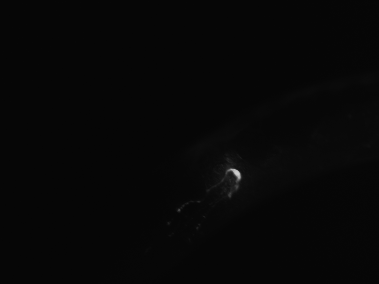

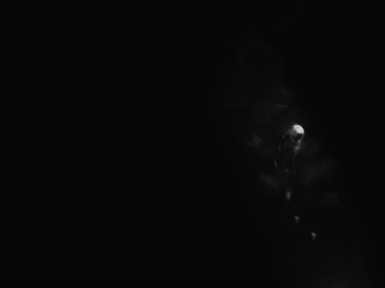

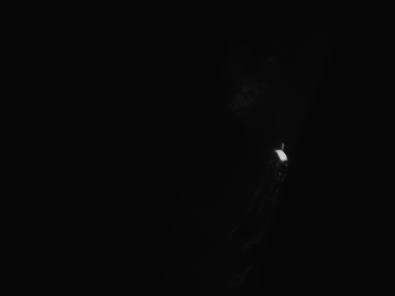

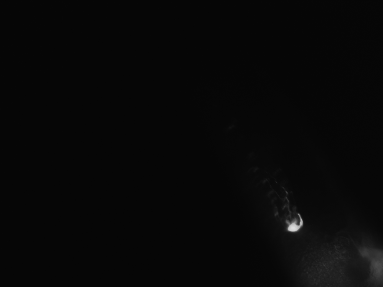

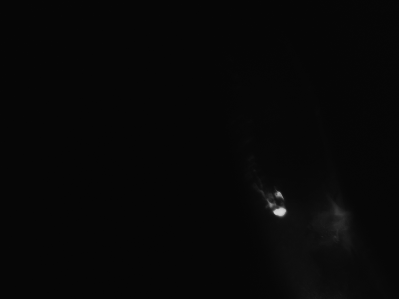

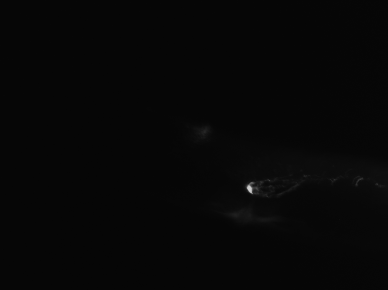

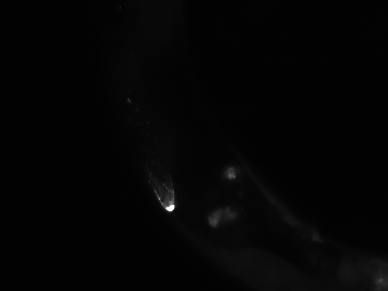

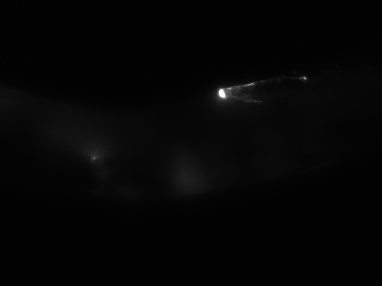

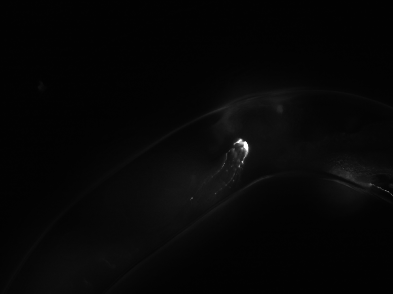

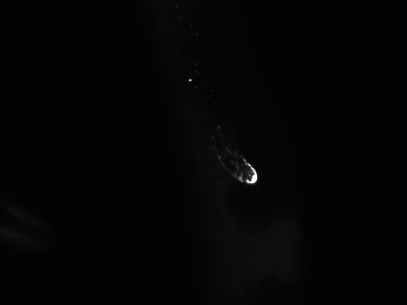

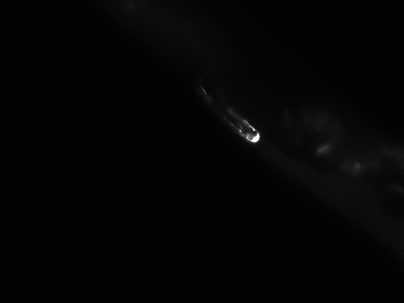

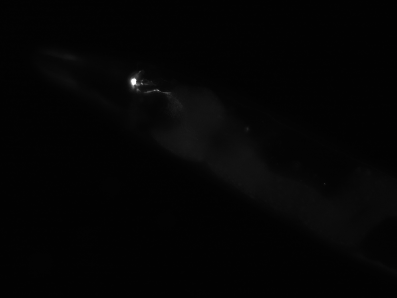

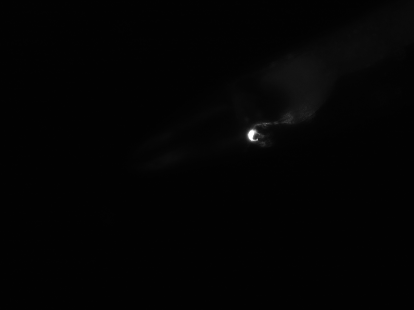

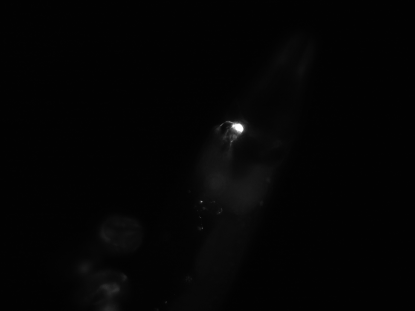

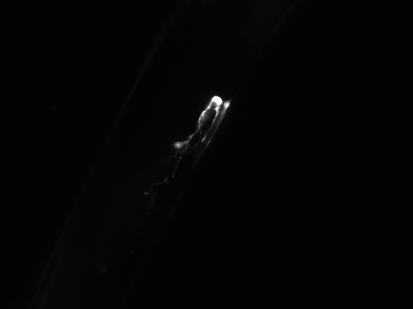


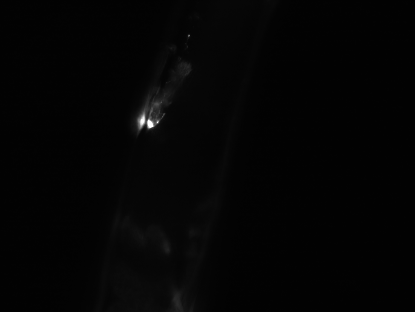


Fig. 2F Control


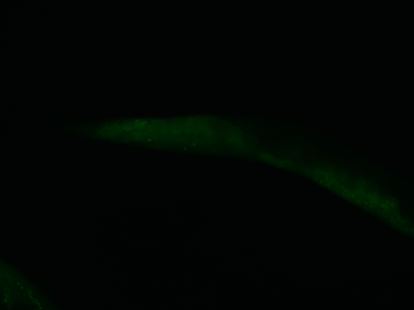

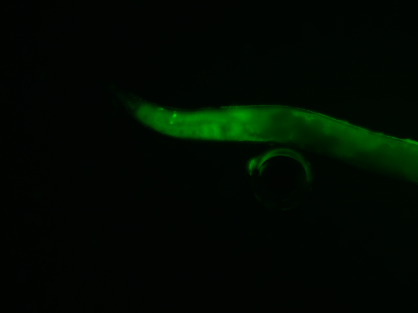

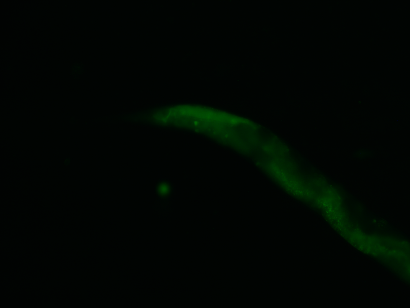

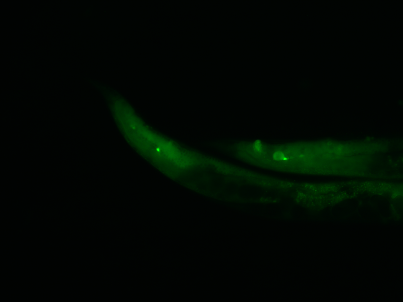

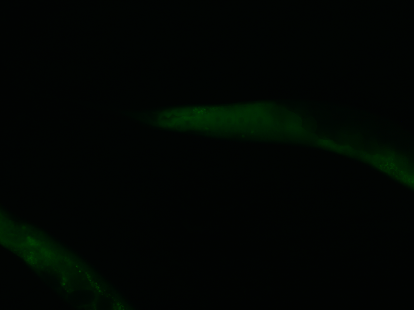

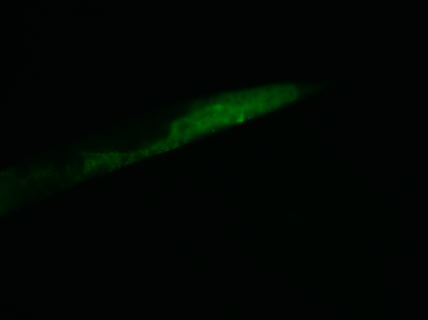

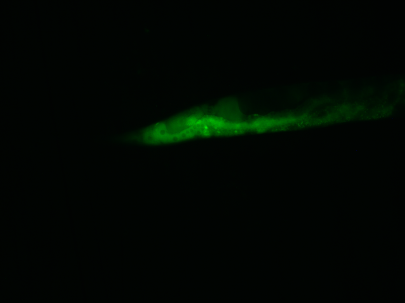

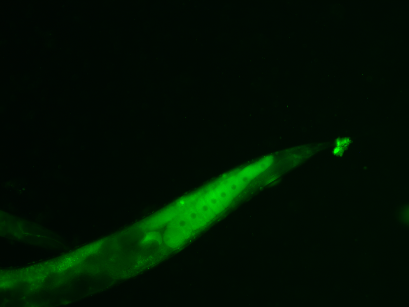

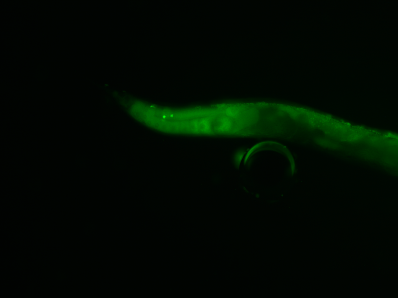


Fig. 2F BPA


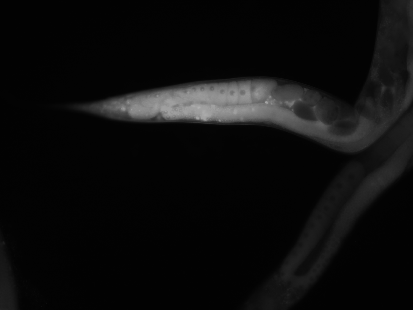

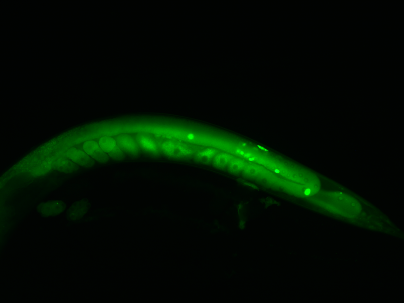

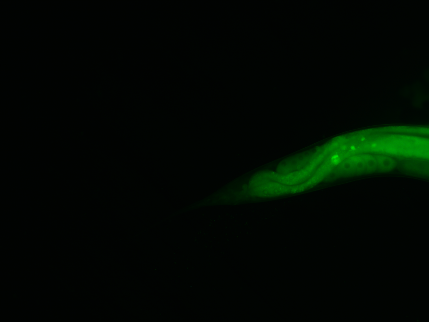

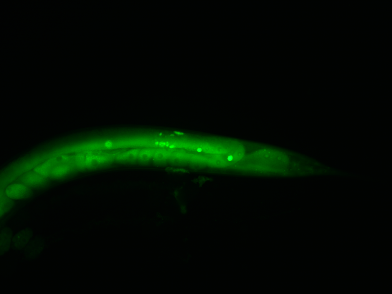

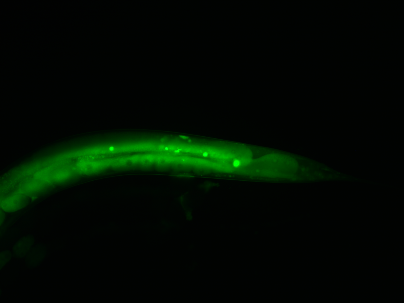

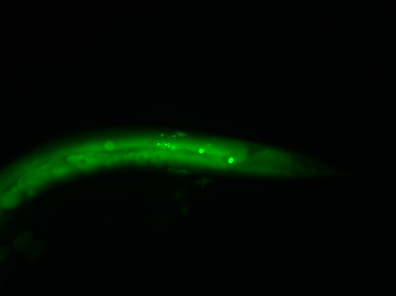

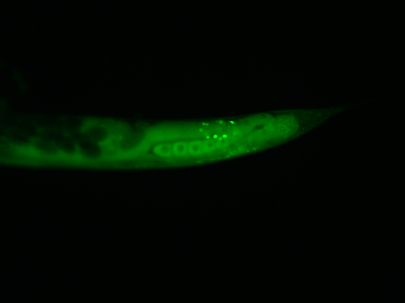

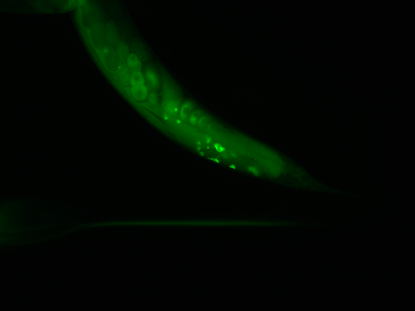

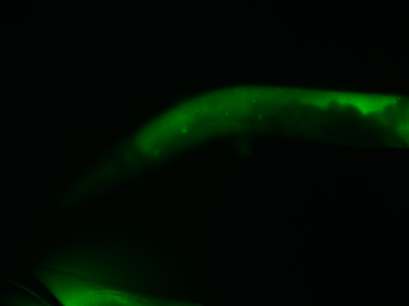

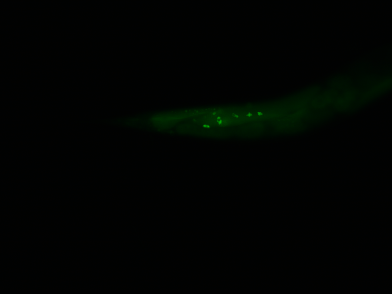

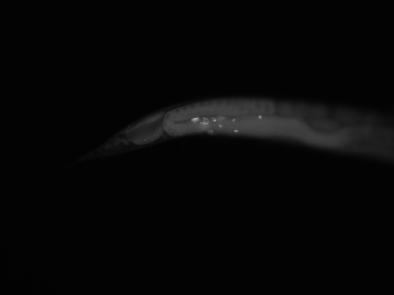

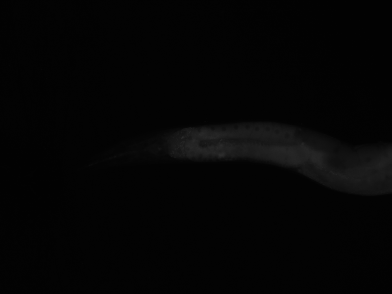

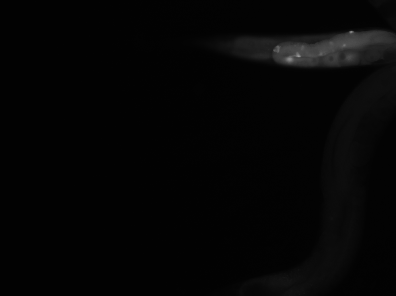

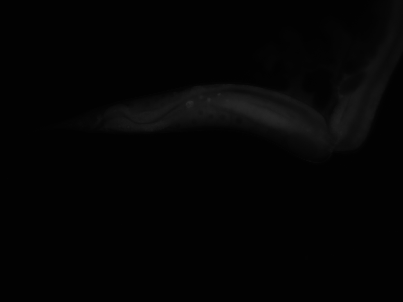

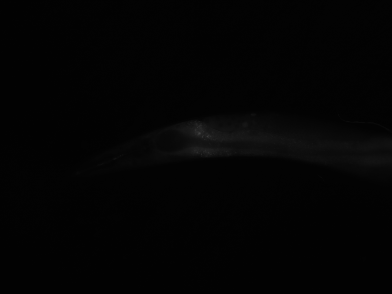

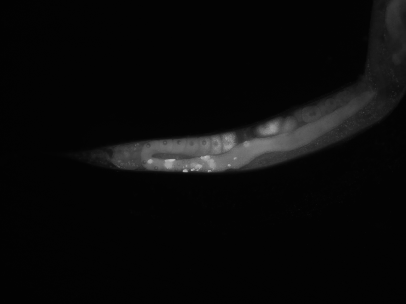

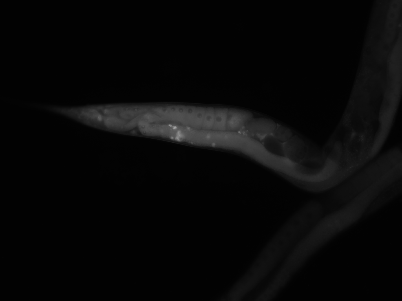

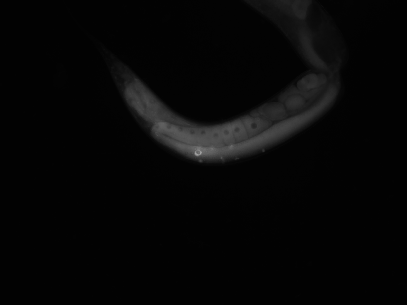


Fig. 2F BPA+BSHX


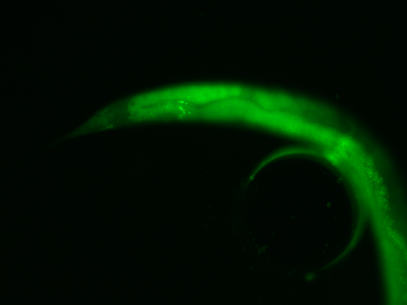

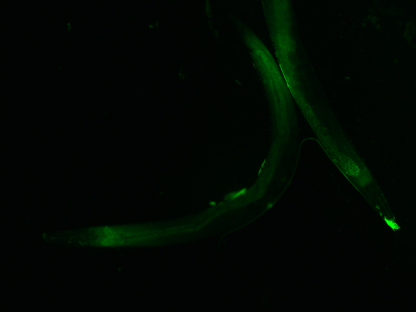

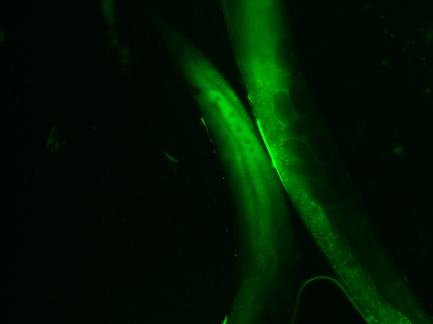

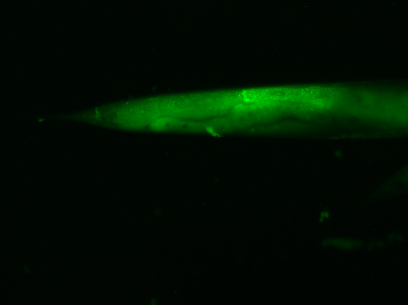

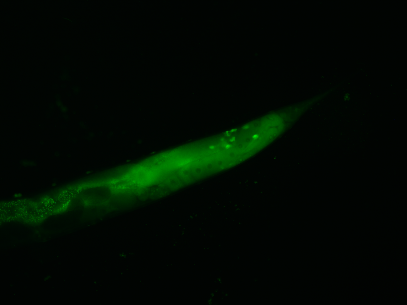

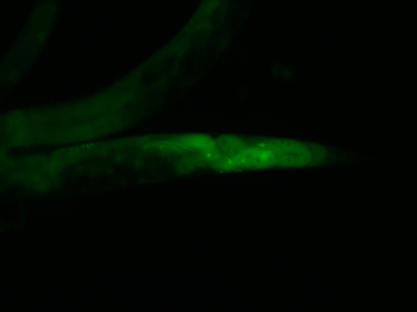

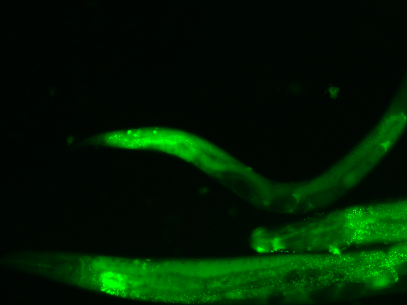

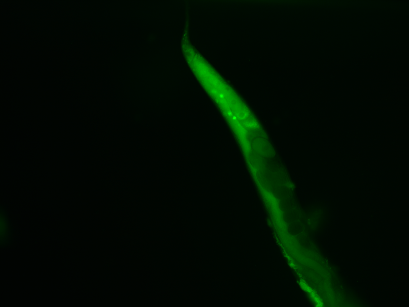

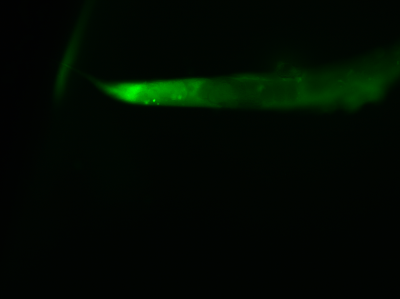

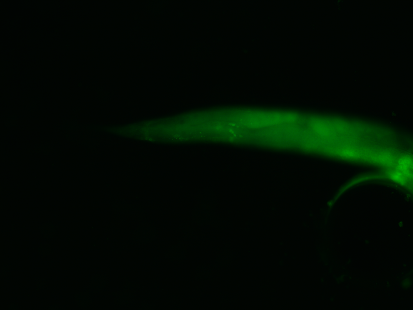

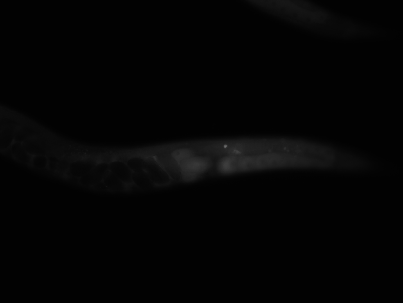

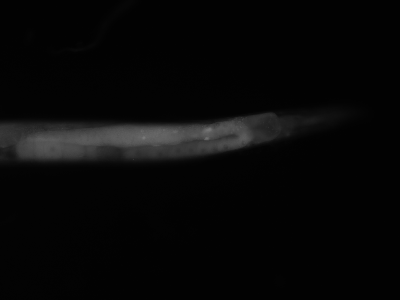

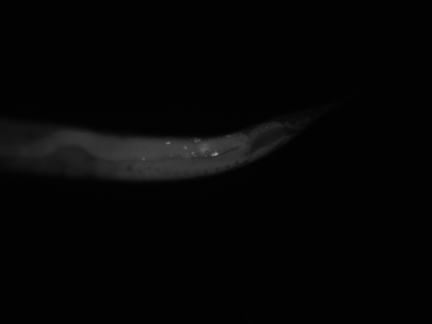


Fig. 2G Control


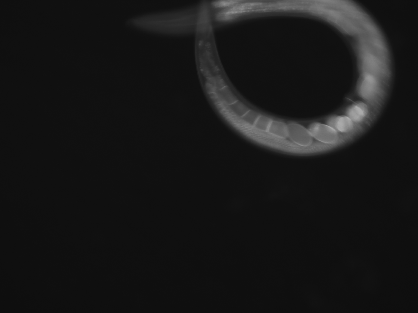

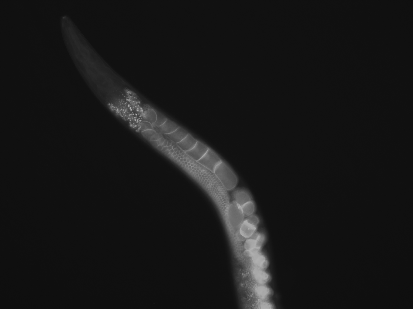


Fig. 2G BPA

Fig. 2G BPA+BSHX
