# Supplementary material for: BuShen HuoXue decoction improves fertility through intestinal hsp-16.2-mediated heat-shock signaling pathway in Caenorhabditis elegans
Source: Front Pharmacol. 2023 Jun 2;14:1210701. doi: 10.3389/fphar.2023.1210701 (PMC10272376; doi:10.3389/fphar.2023.1210701)

1. **Total ion chromatograms of mixed standards by LC-Q/TOF operated in the negative ionization mode**


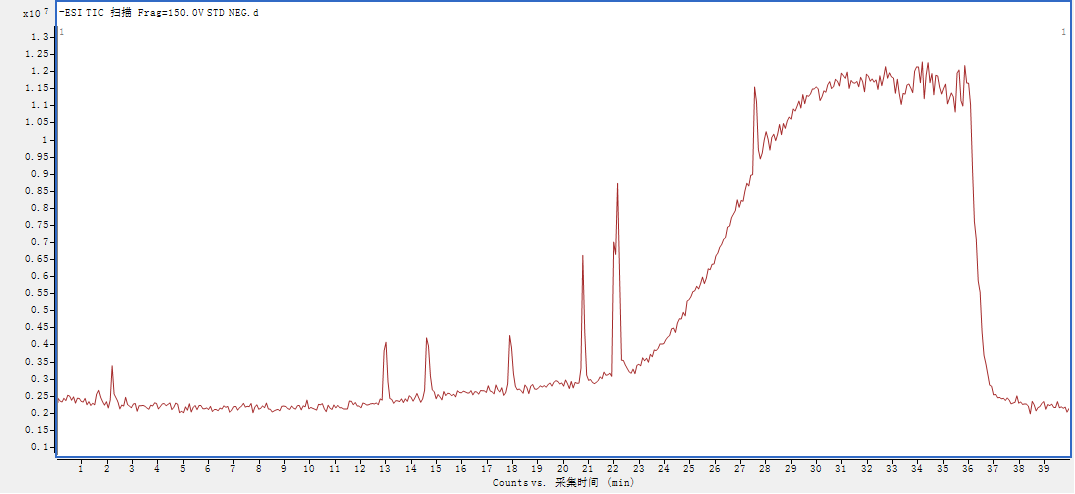


1. Total ion chromatograms of  Bushen Huoxue decoction sample by LC-Q/TOF operated in the negative ionization mode


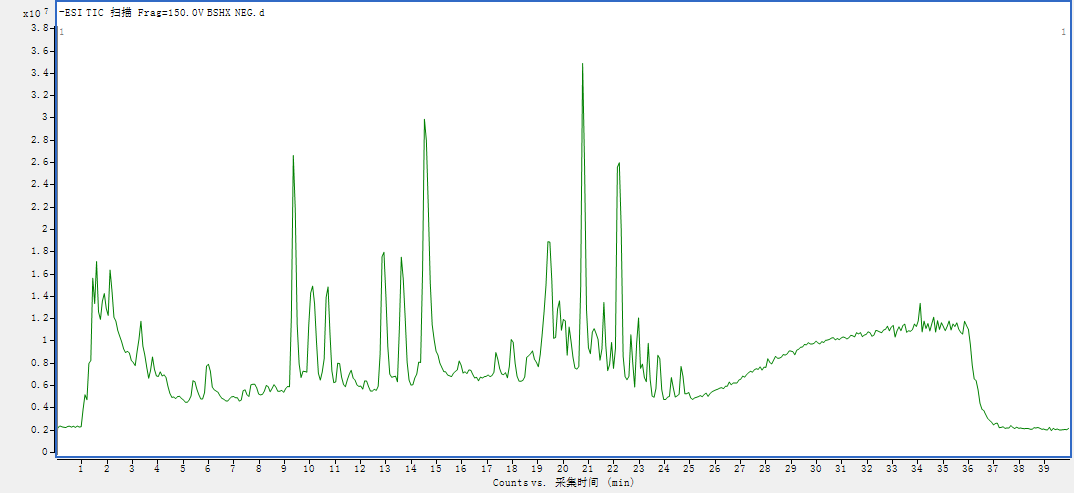


1. Total ion chromatograms of Bushen Huoxue-treated nematodes sample by LC-Q/TOF operated in the negative ionization mode


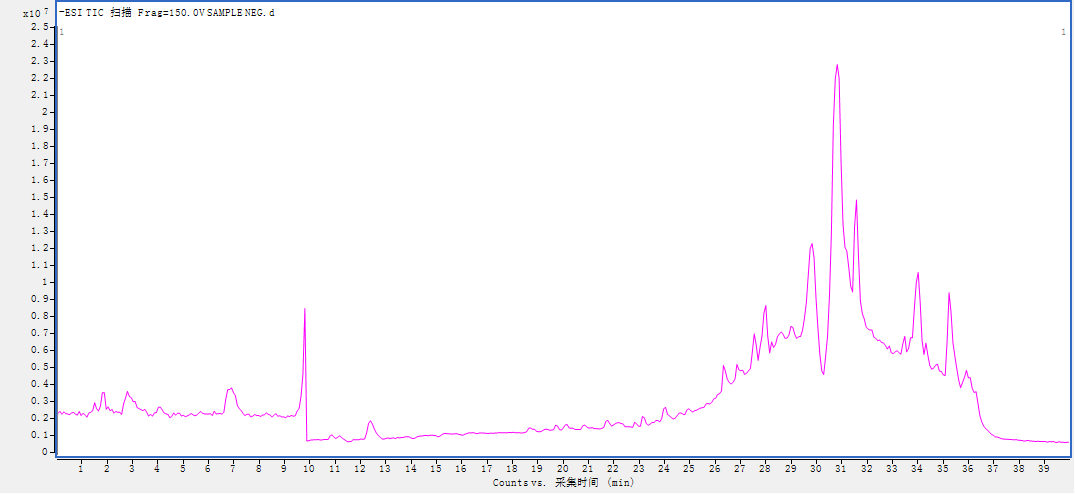

Supplement: Supplementary file 10 [file Table3.DOCX]
