# Supplementary material for: BuShen HuoXue decoction improves fertility through intestinal hsp-16.2-mediated heat-shock signaling pathway in Caenorhabditis elegans
Source: Front Pharmacol. 2023 Jun 2;14:1210701. doi: 10.3389/fphar.2023.1210701 (PMC10272376; doi:10.3389/fphar.2023.1210701)
Supplement: Supplementary file 11 [file Table13.DOCX]

Fig. 5 C Control


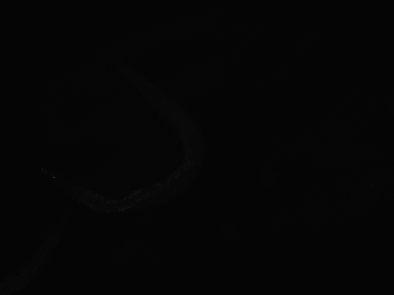

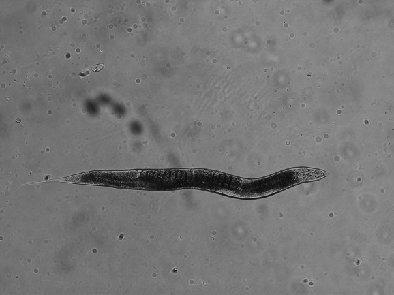

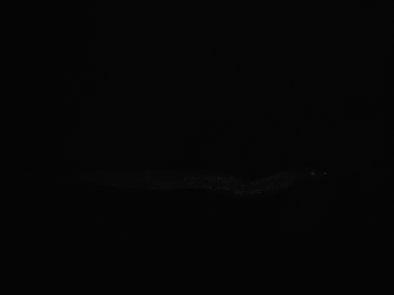

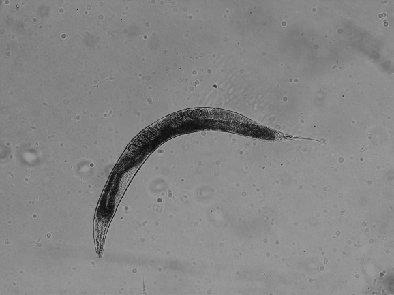

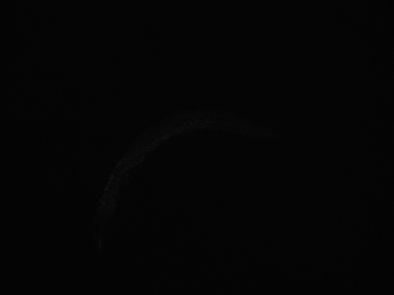

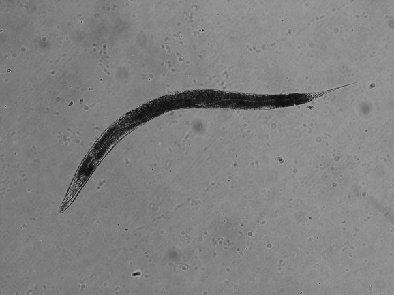

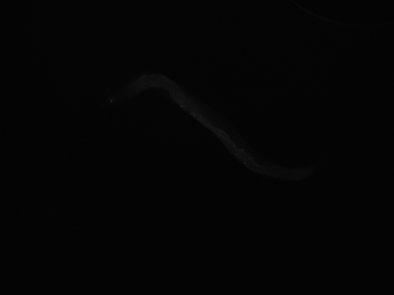

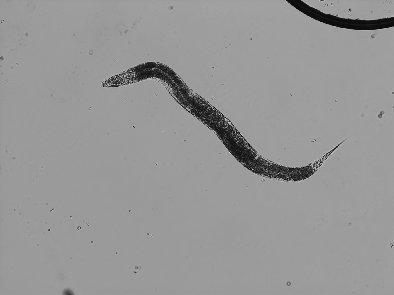


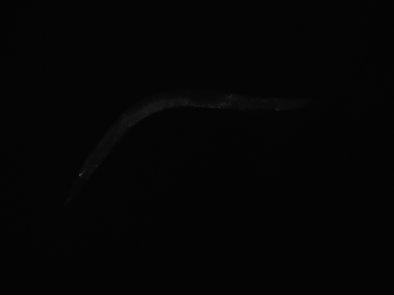

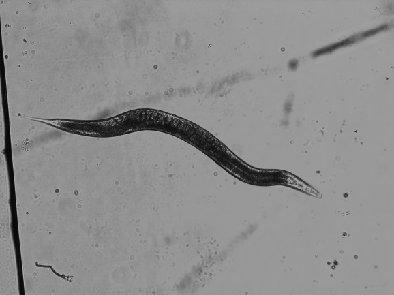

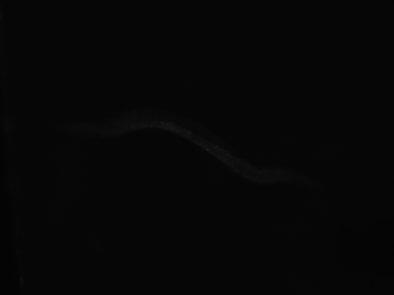

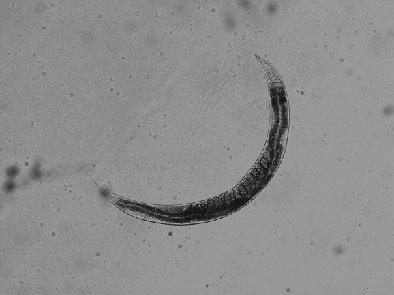

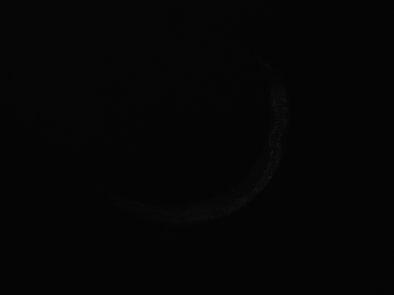

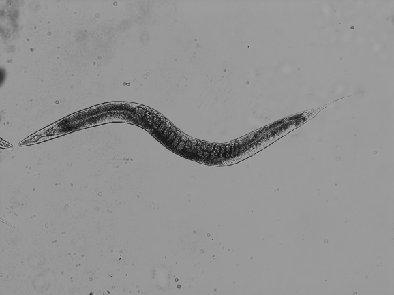

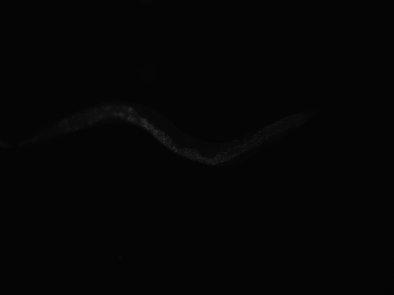

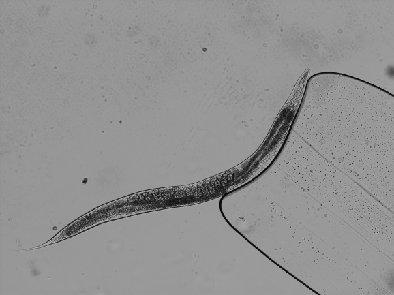

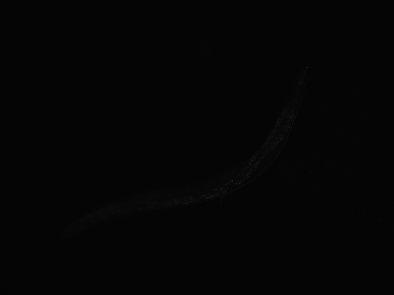

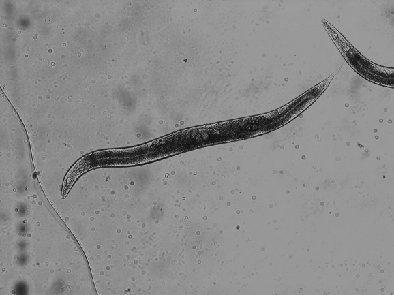

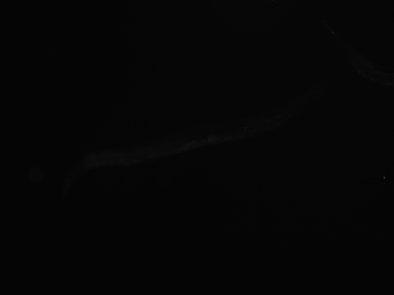

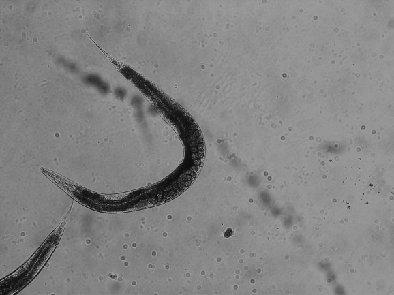


Fig. 5 C BPA


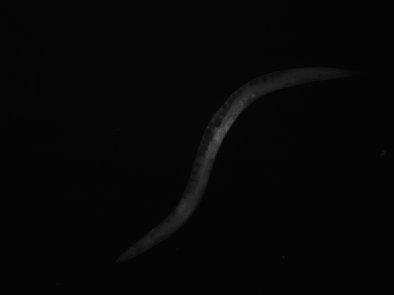

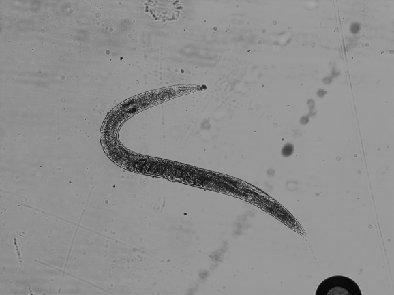

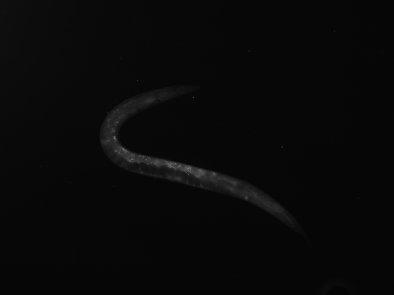


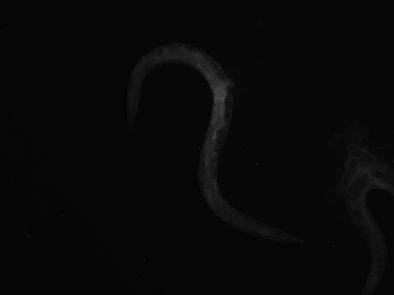

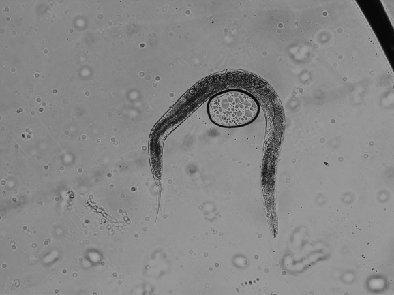

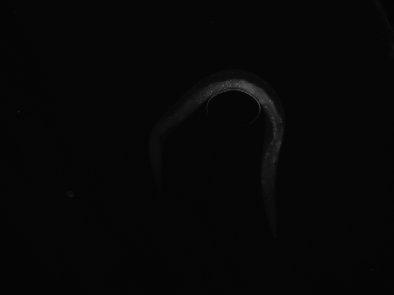

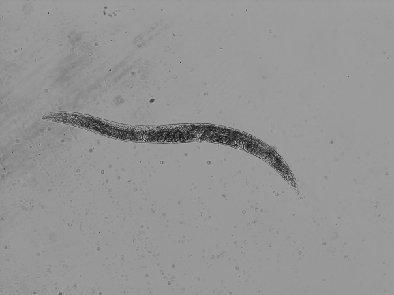

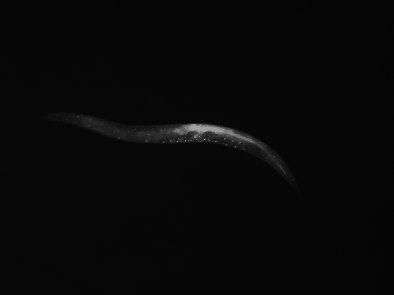

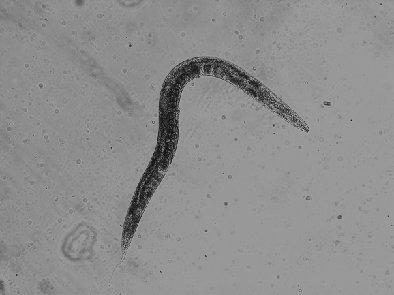

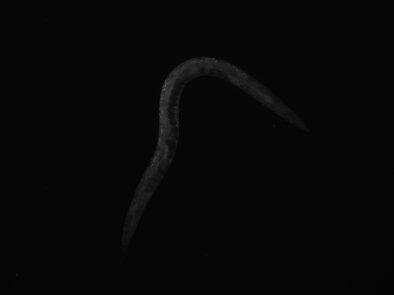

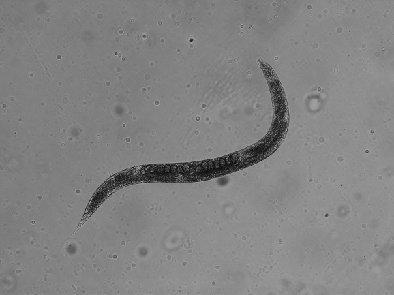

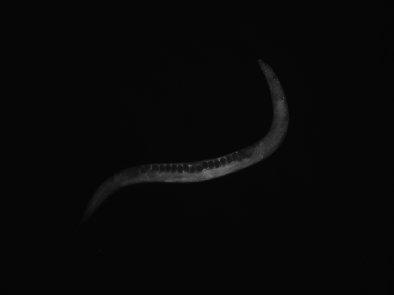

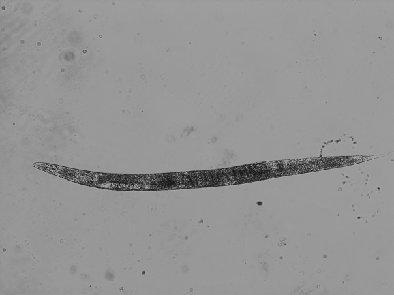

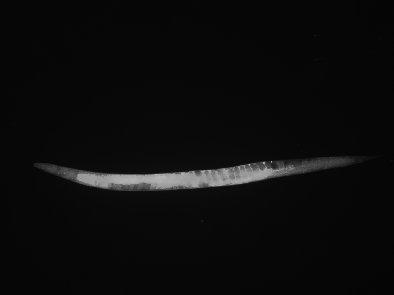

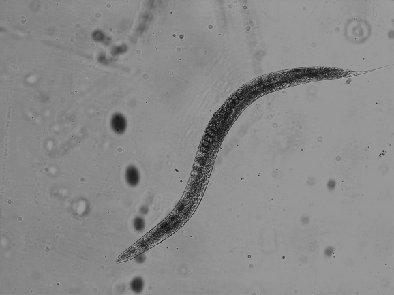


Fig. 5 C BPA+BSHX


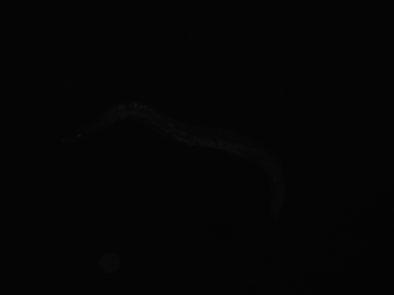

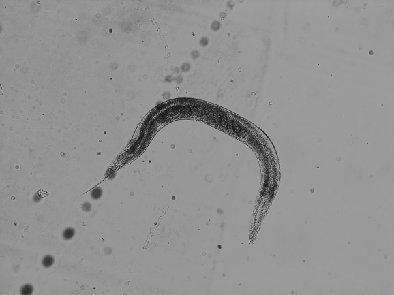

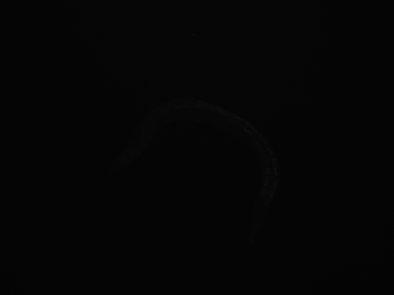

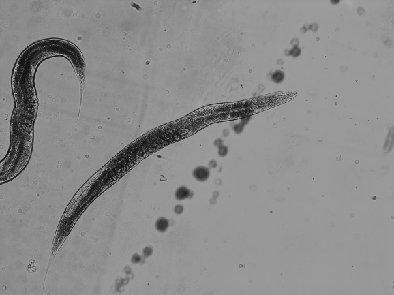

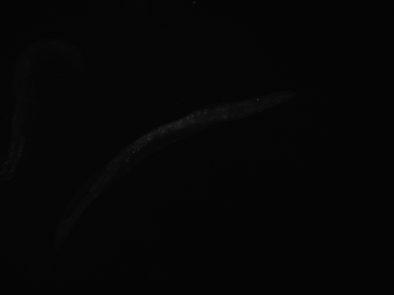


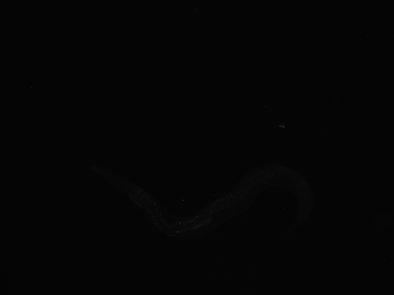

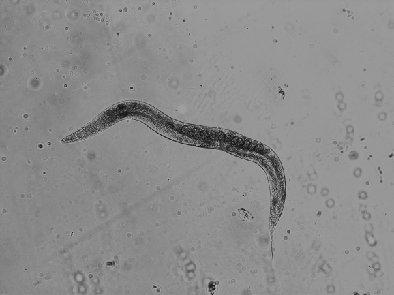


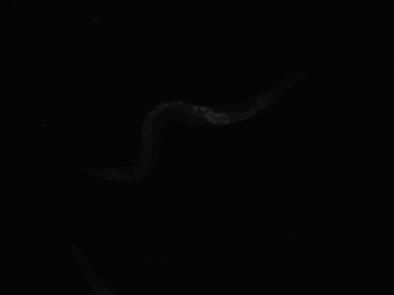

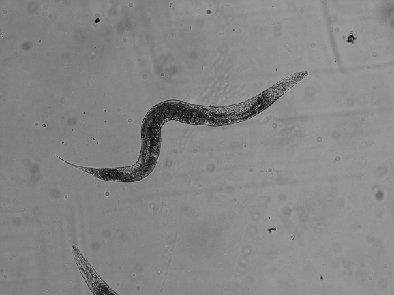

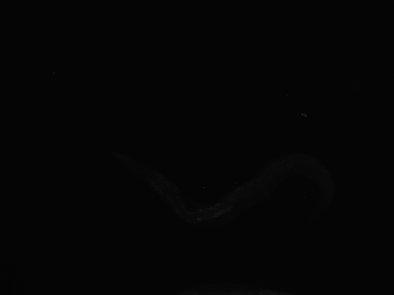

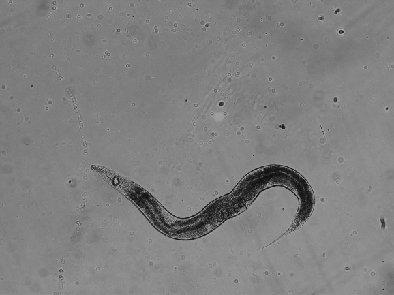

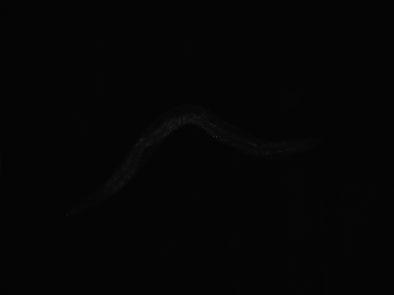

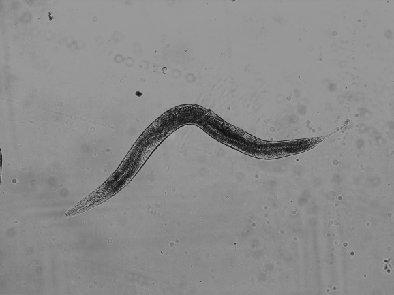

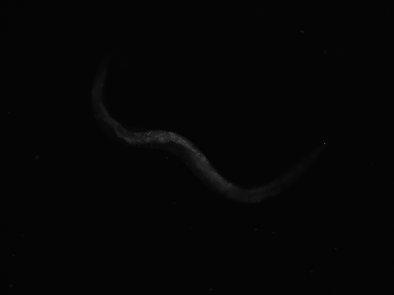

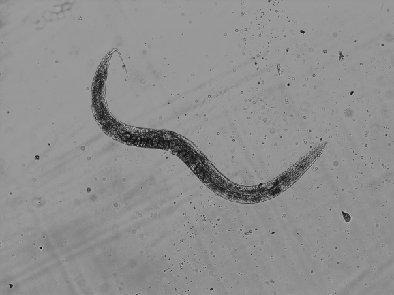

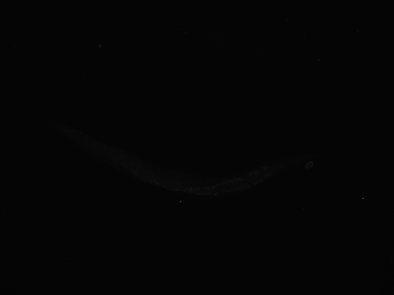

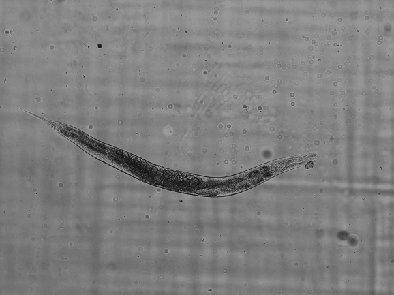


Fig. 5 C BPA+BSHX+hsp-16.2 RNAi


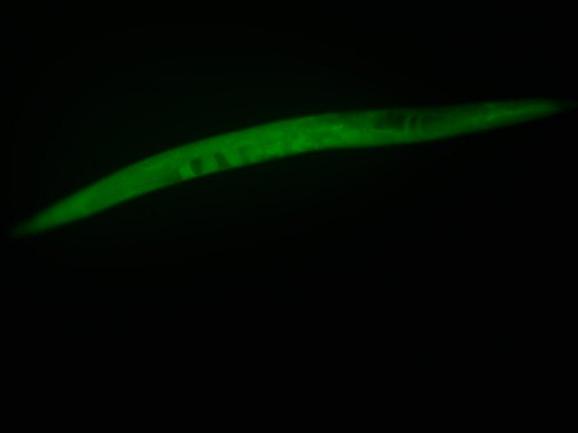

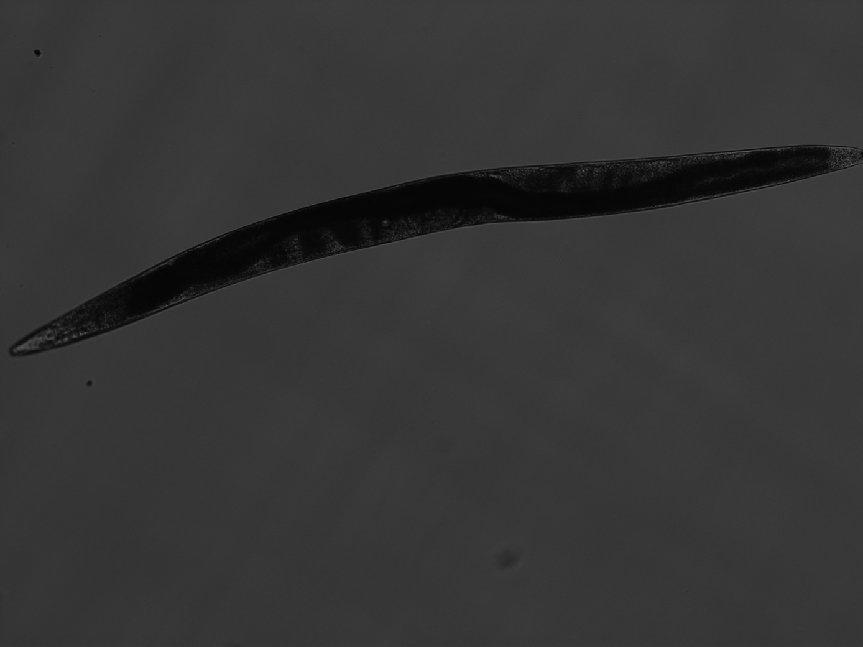

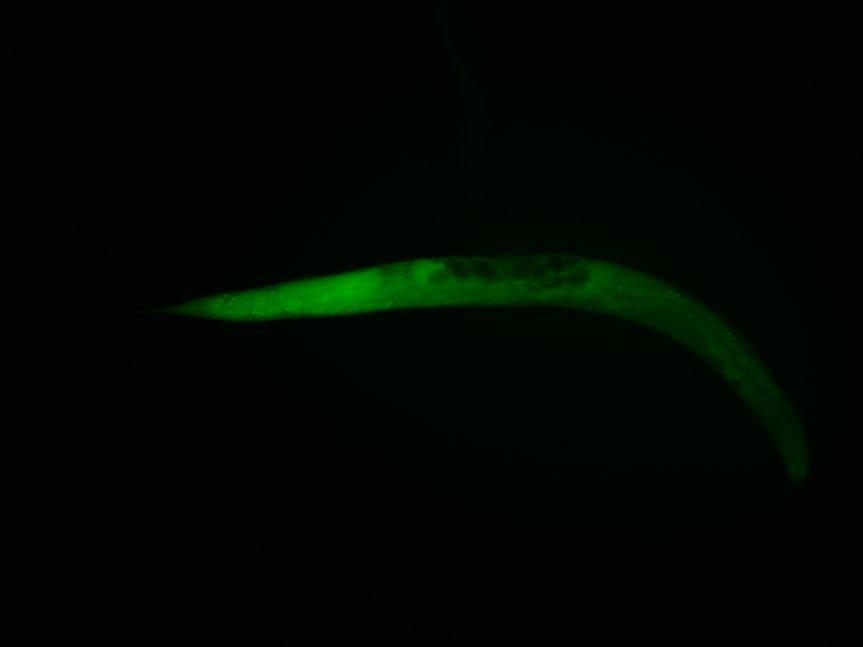

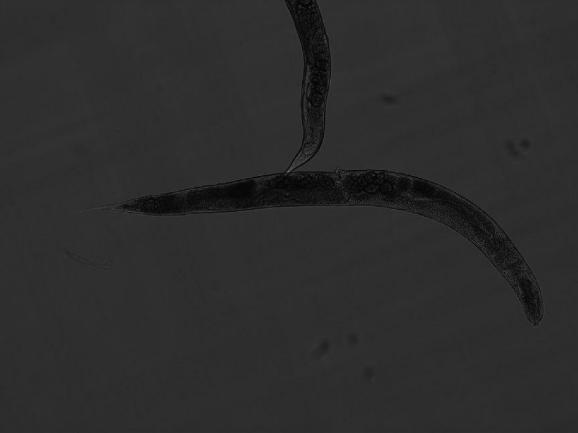

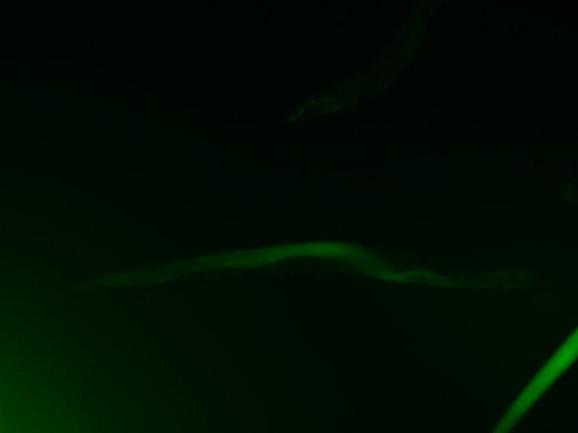

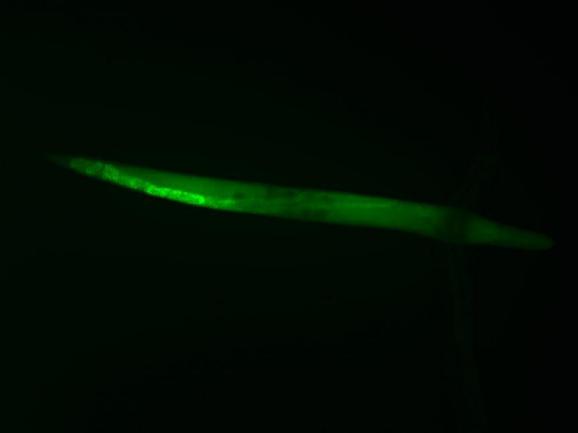

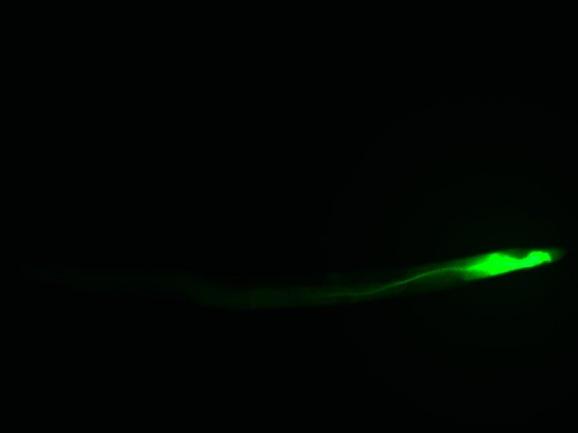

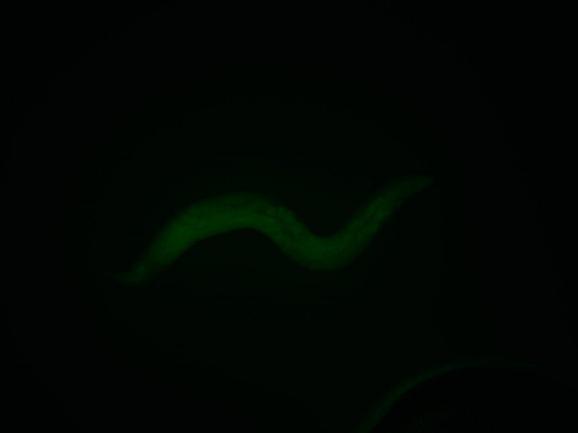

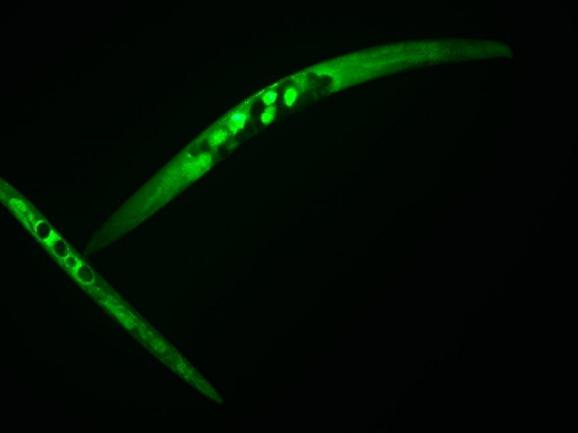

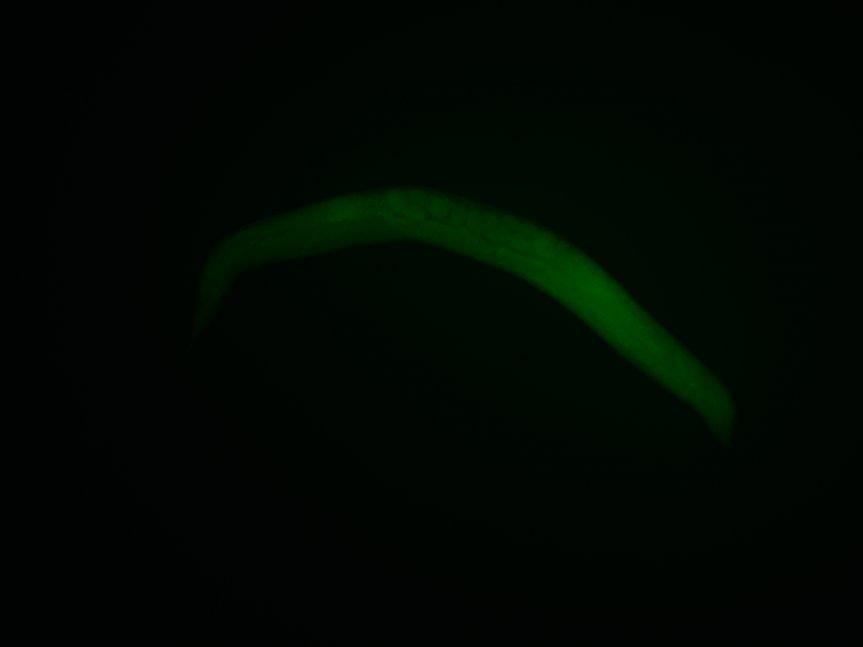

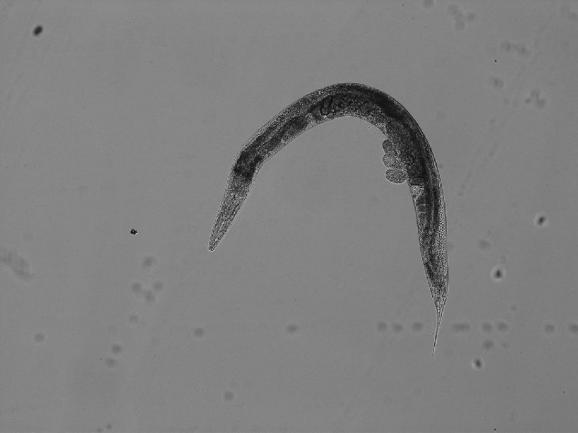

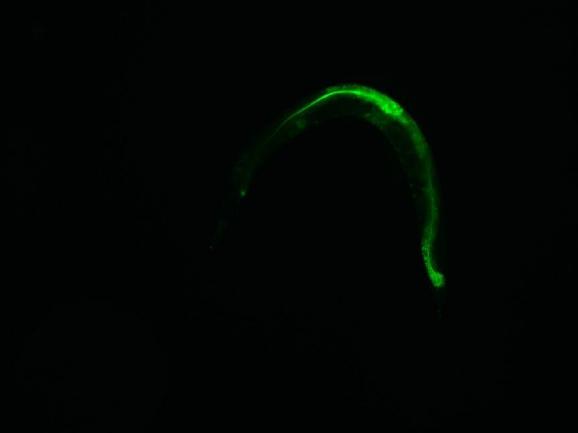

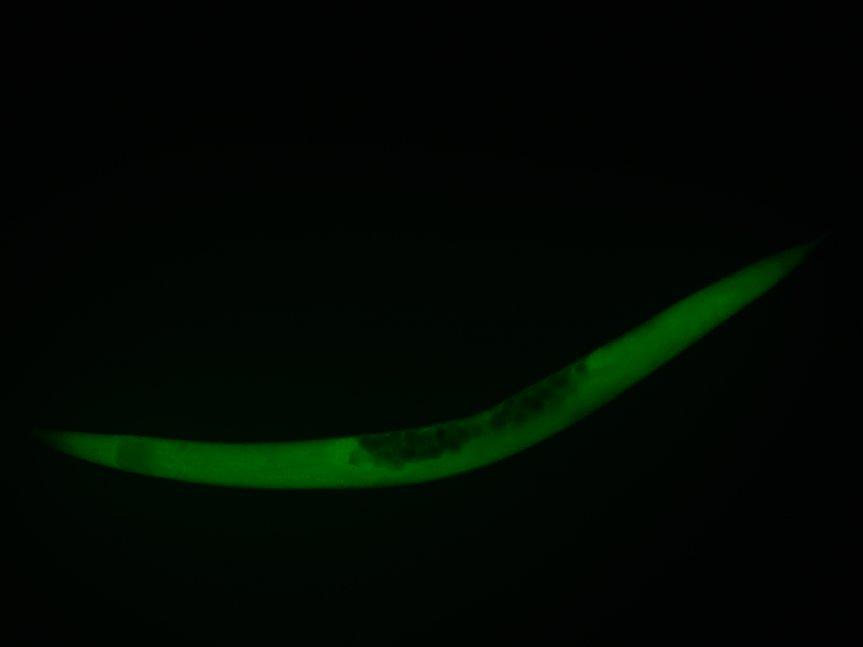

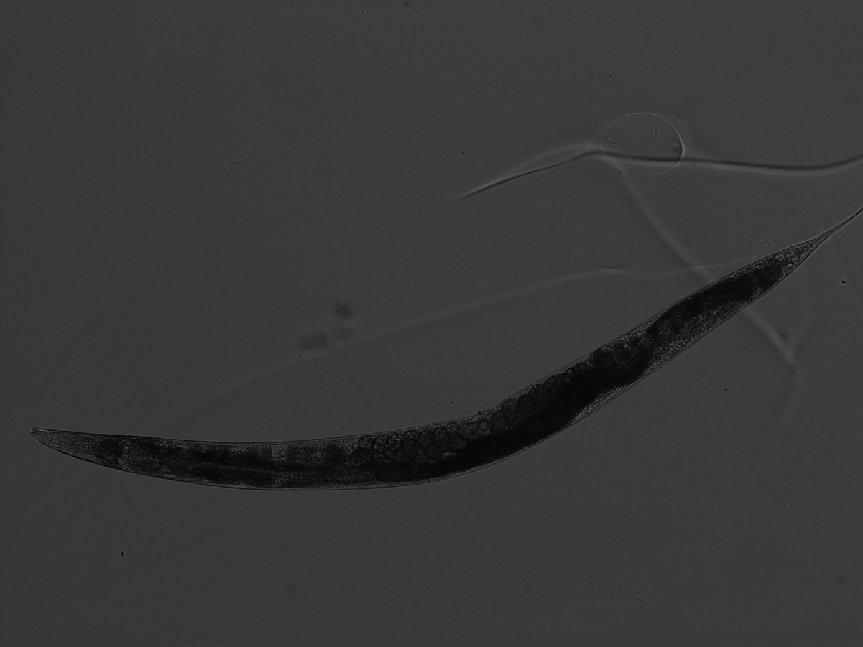

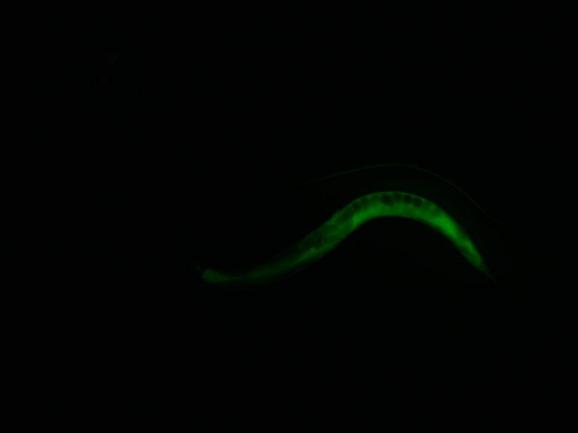

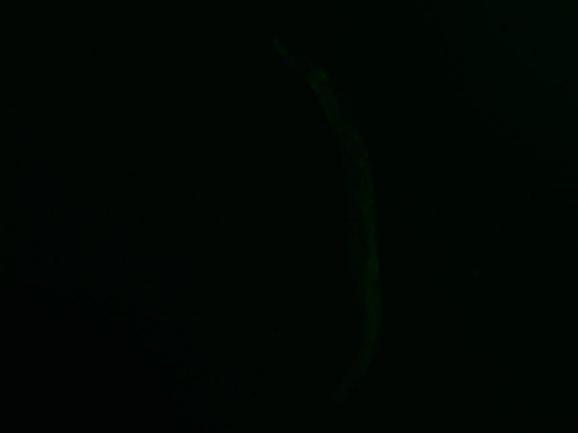

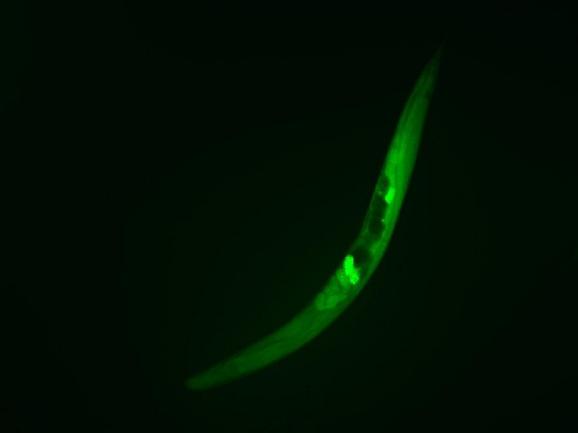


Fig. 5 D Control


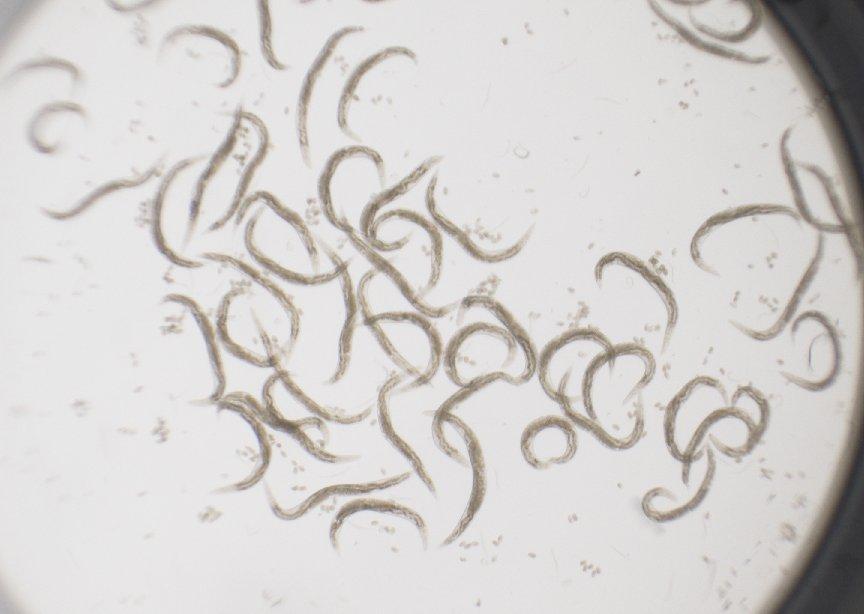

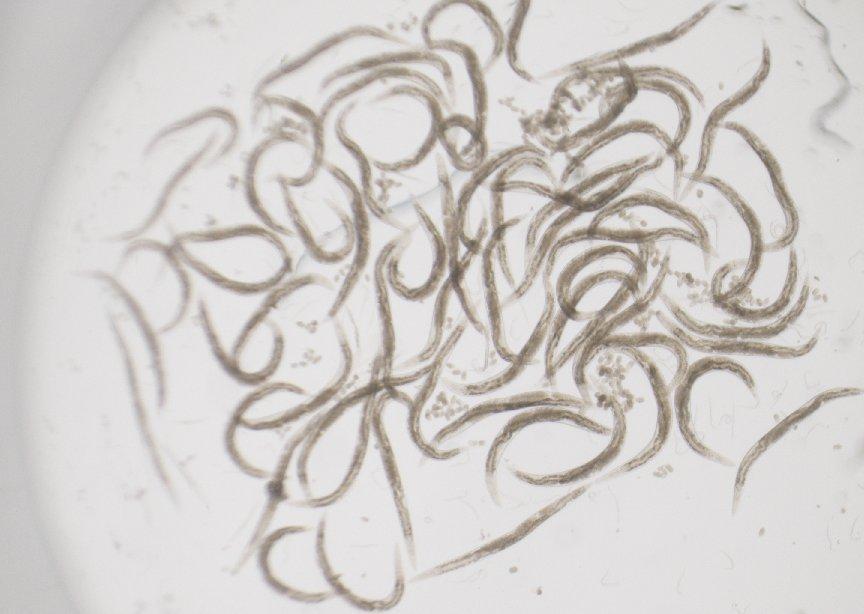

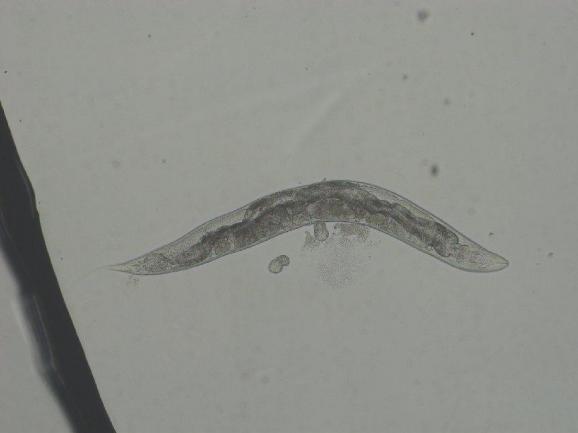

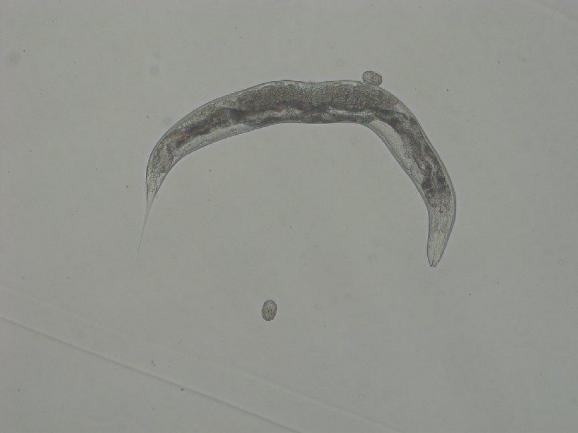

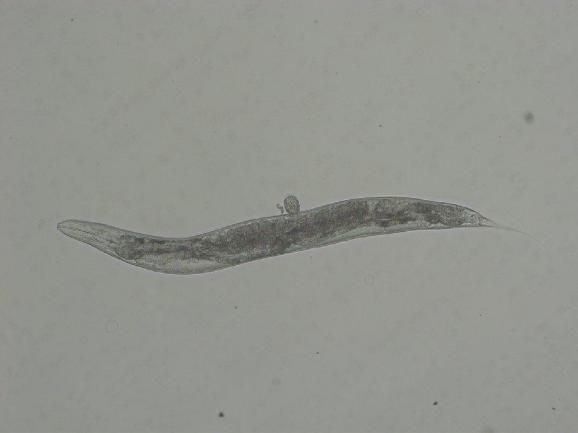

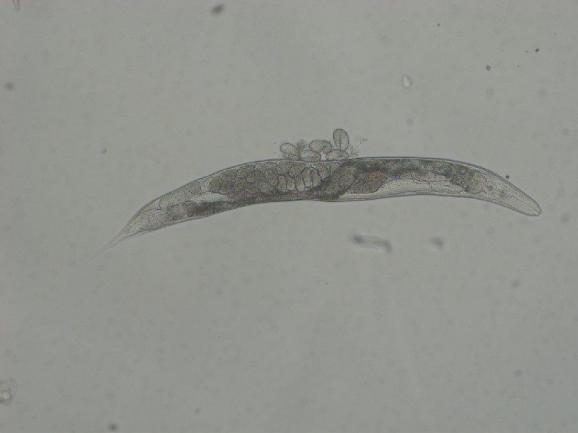

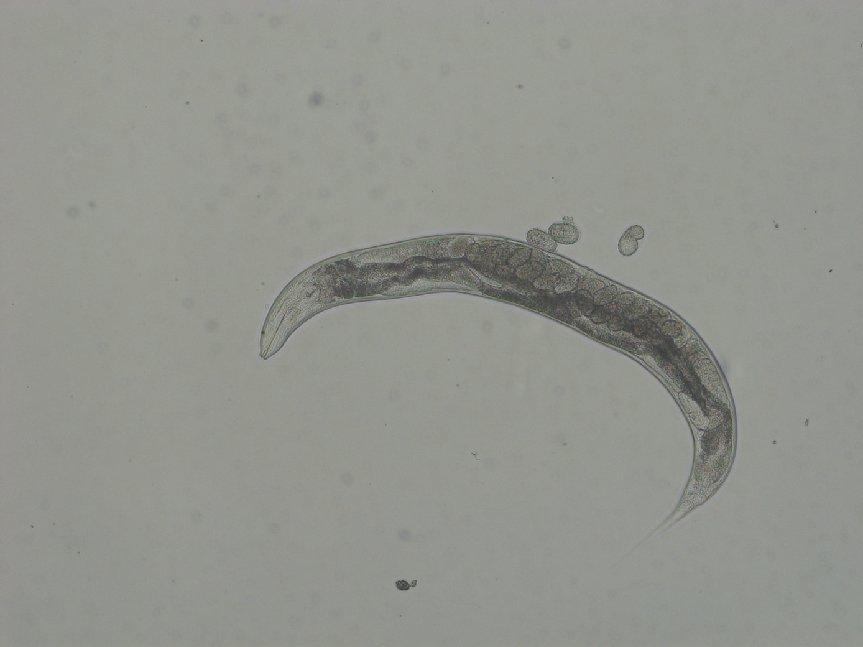

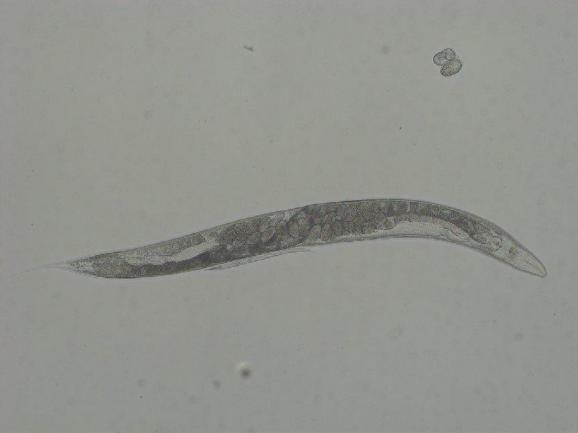

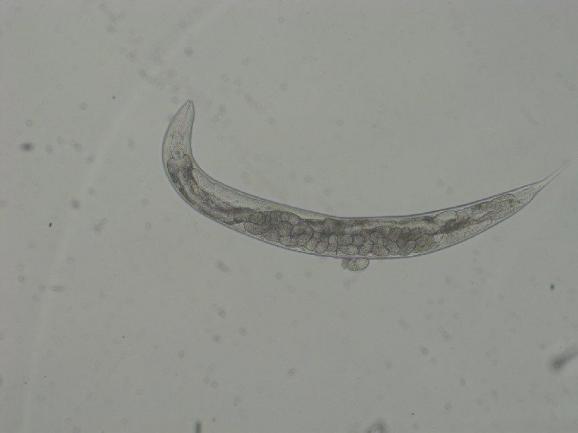


Fig. 5 D Control +Erioglaucine disodium


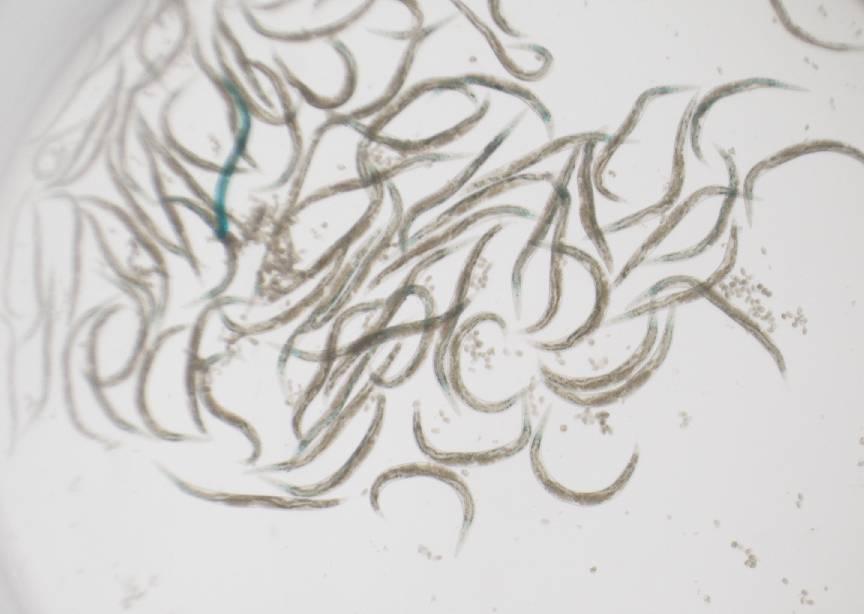

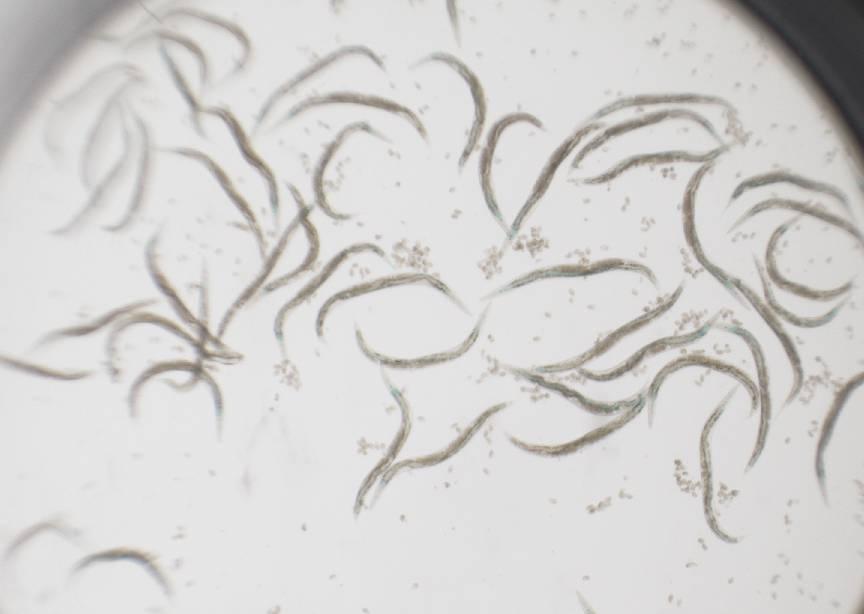

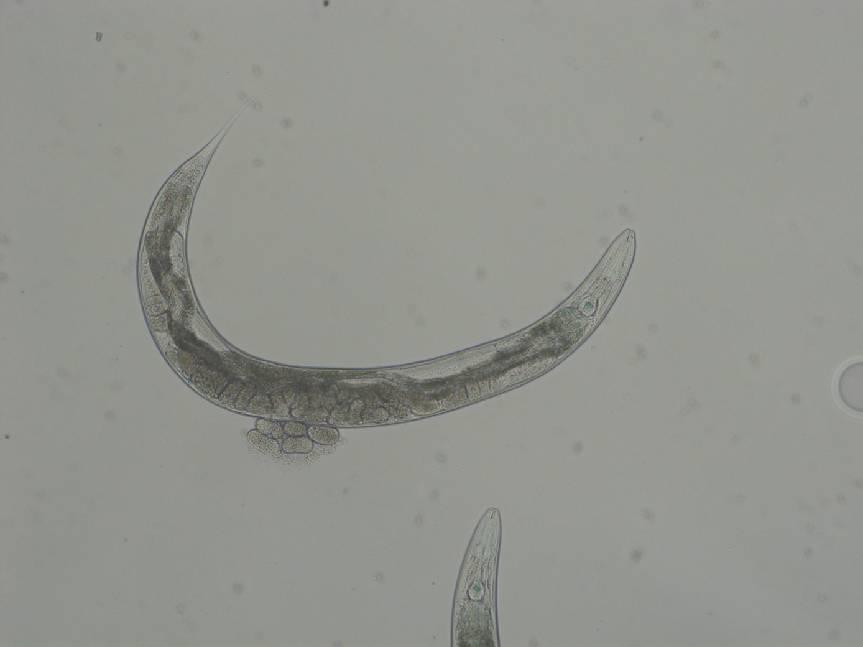

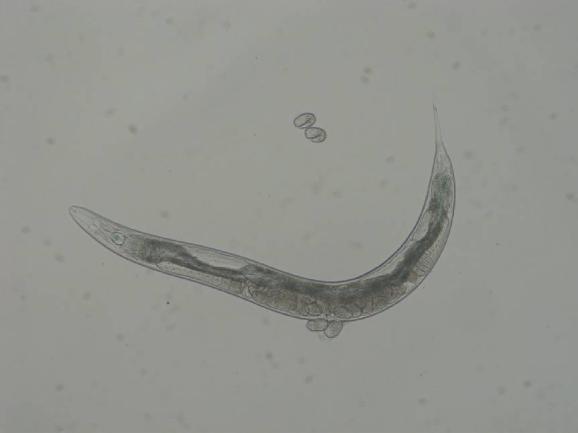

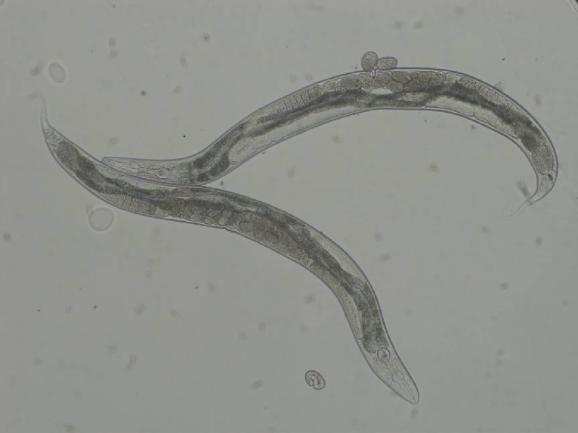

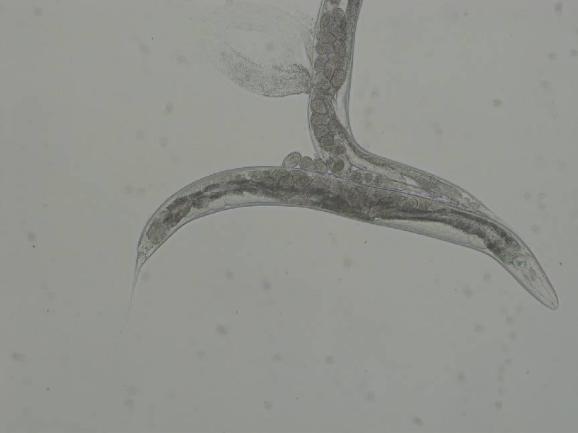

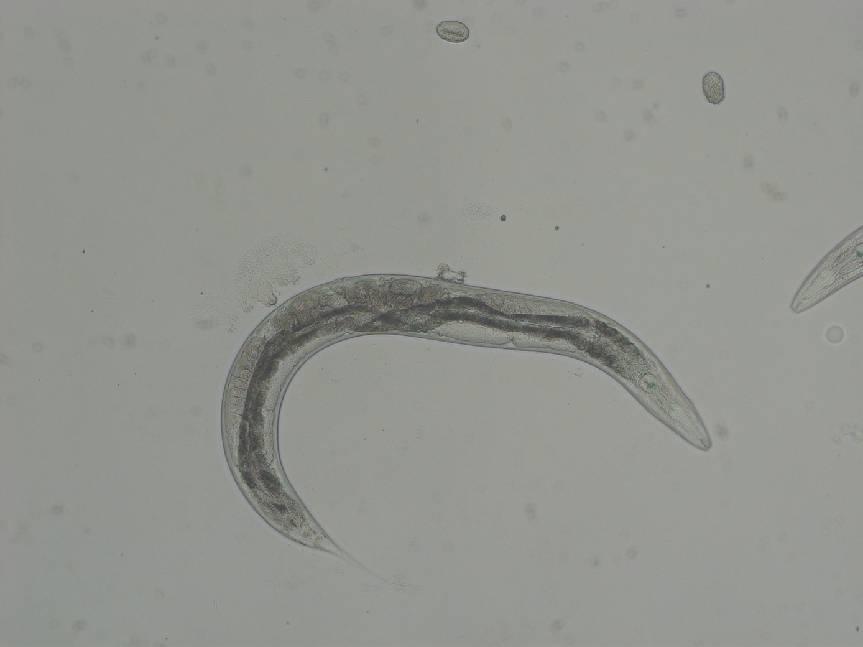

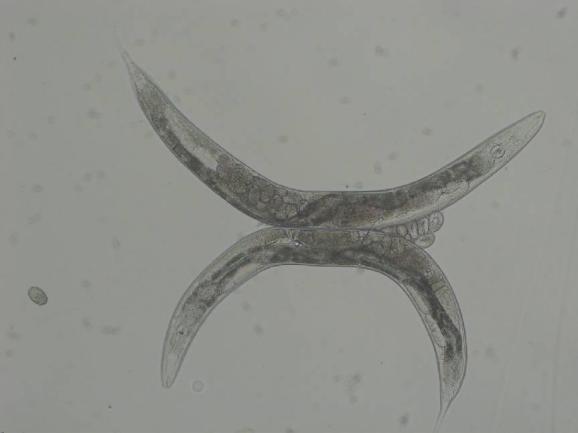

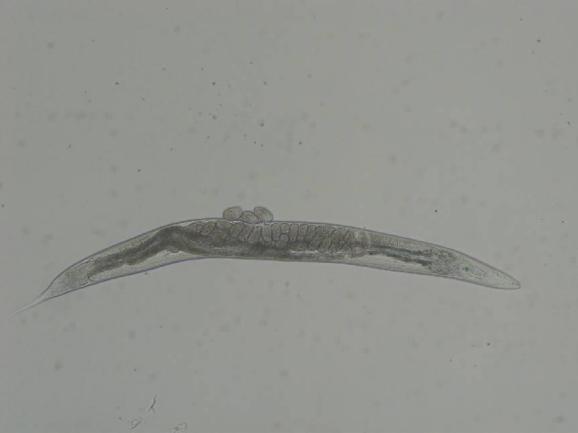


Fig. 5 D BPA+ Erioglaucine disodium


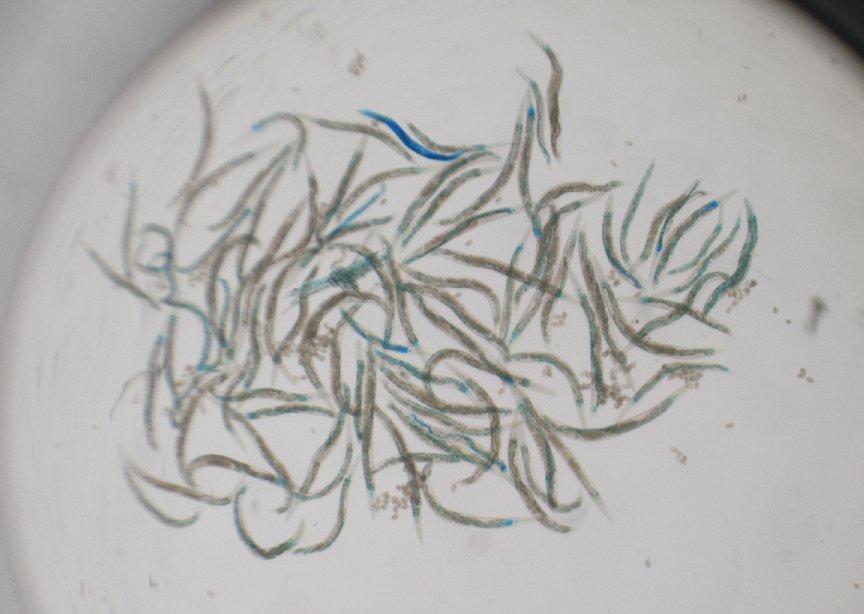

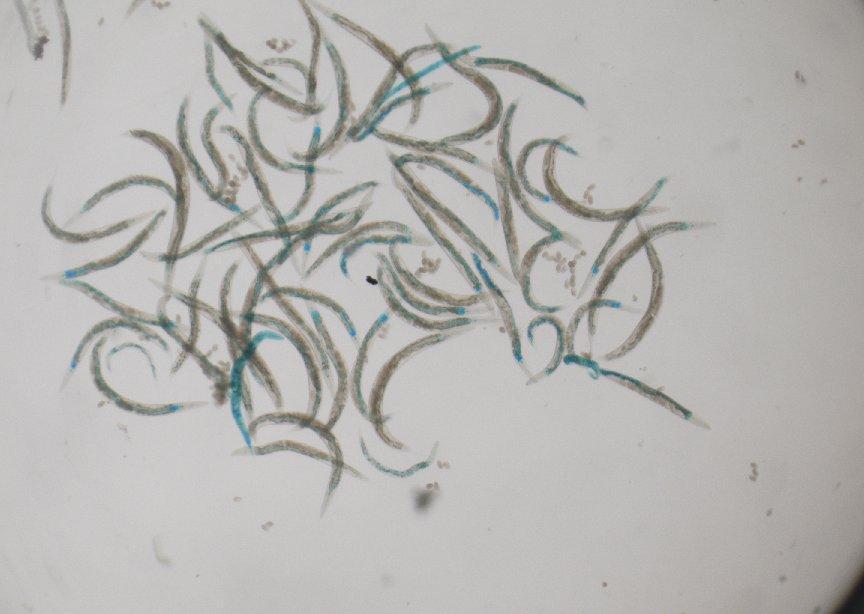

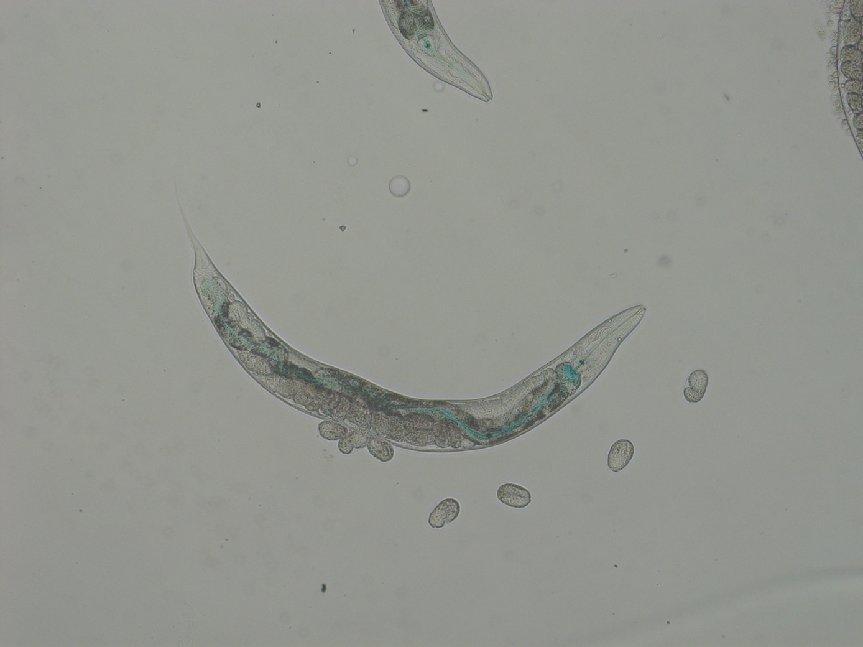

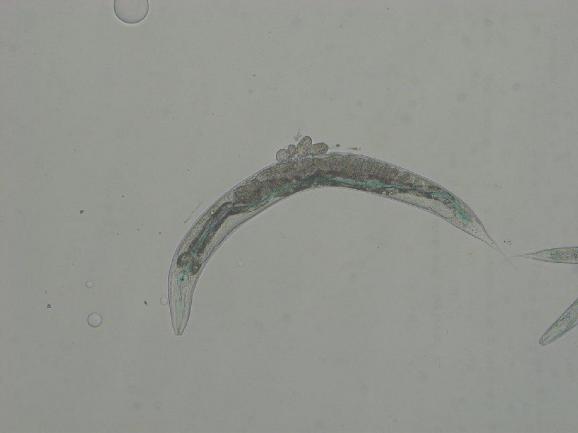

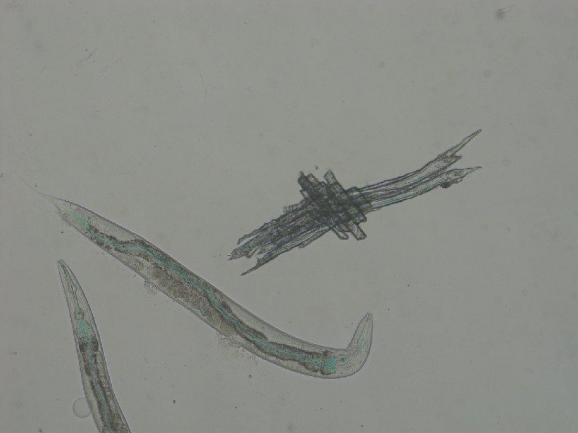

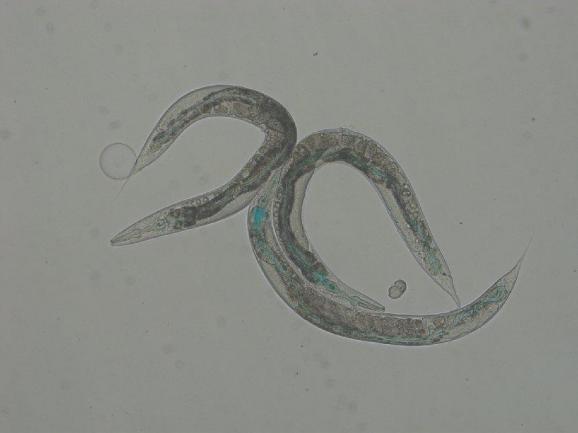

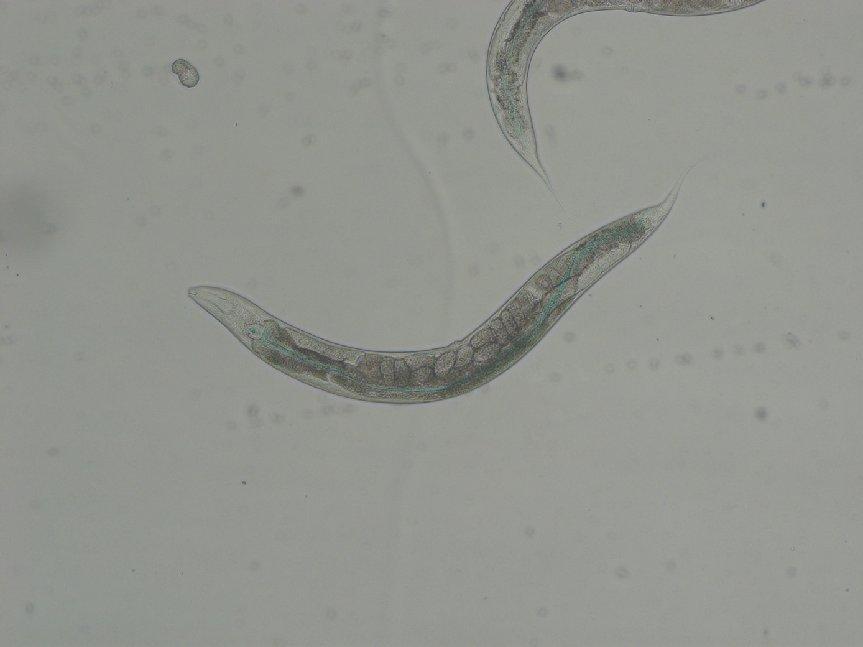

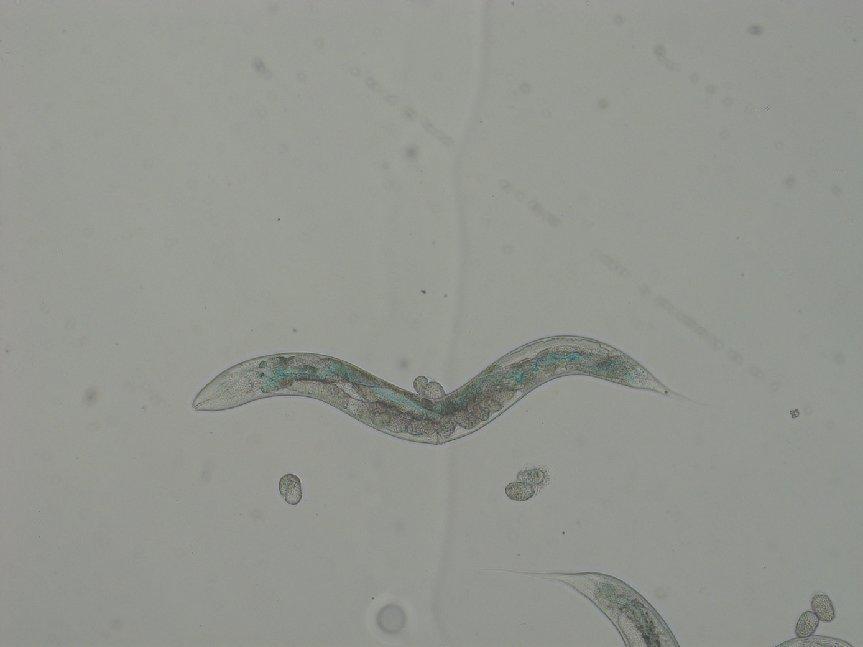

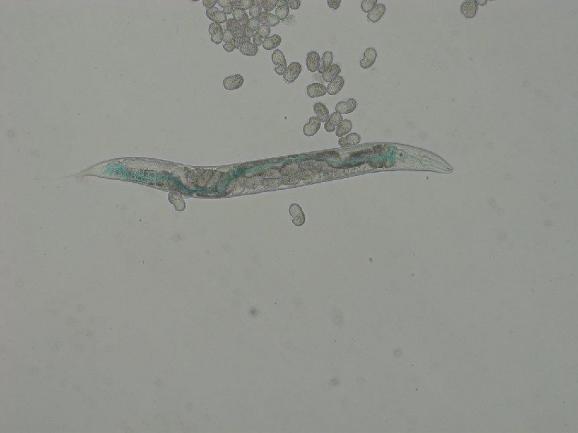


Fig. 5 D BPA+BSHX + Erioglaucine disodium


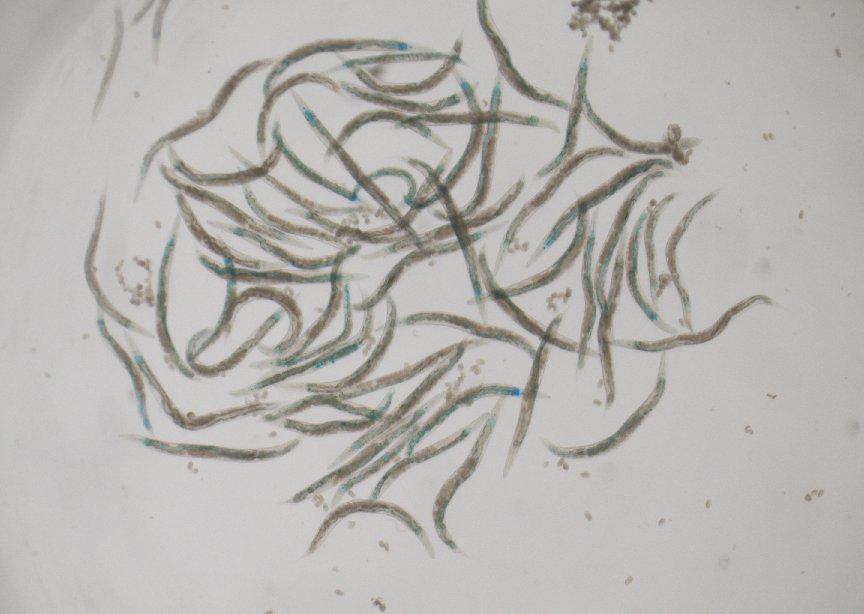

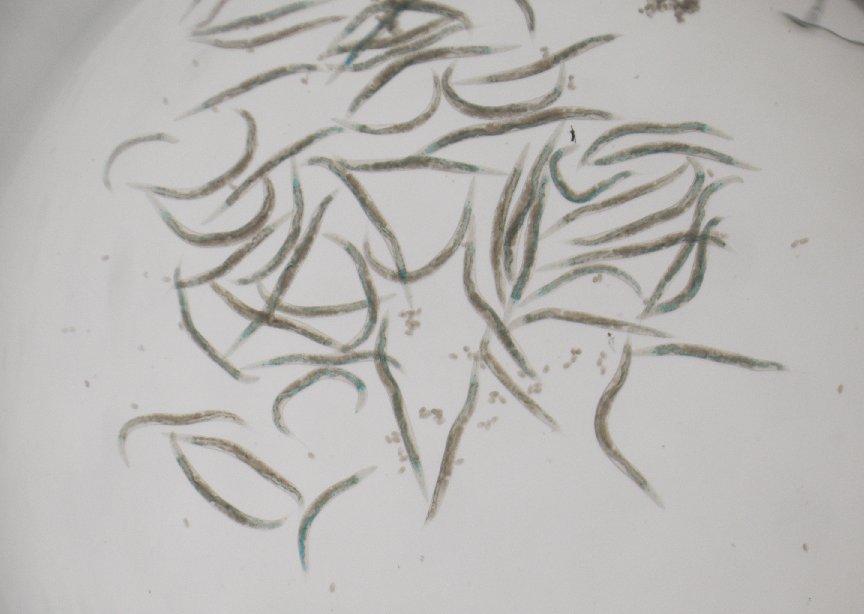

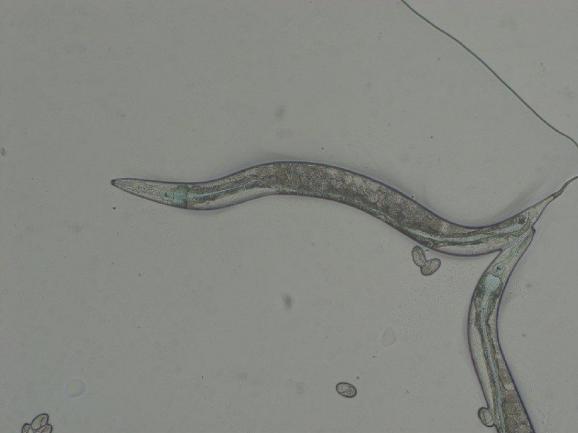

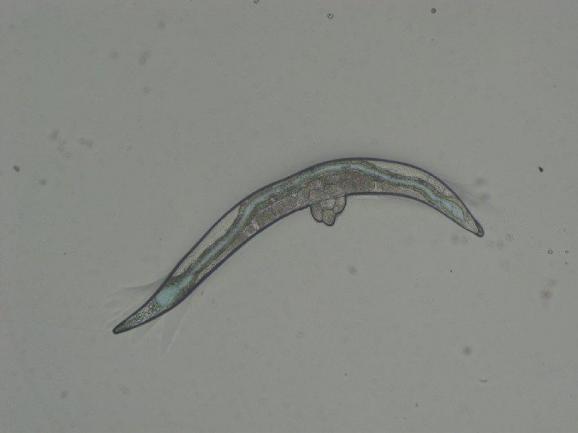


Fig. 5 D BPA+BSHX+hsp-16.2 RNAi+ Erioglaucine disodium
