# Supplementary material for: BuShen HuoXue decoction improves fertility through intestinal hsp-16.2-mediated heat-shock signaling pathway in Caenorhabditis elegans
Source: Front Pharmacol. 2023 Jun 2;14:1210701. doi: 10.3389/fphar.2023.1210701 (PMC10272376; doi:10.3389/fphar.2023.1210701)
Supplement: Supplementary file 12 [file Table11.DOCX]

**Fig. 4 A**

| **Tests of Normality** | | | | | | | |
| --- | --- | --- | --- | --- | --- | --- | --- |
|  | Group | Kolmogorov-Smirnov^a^ | | | Shapiro-Wilk | | |
|  |  | Statistic | df | Sig. | Statistic | df | Sig. |
| Brood size | Control | .162 | 7 | .200^*^ | .953 | 7 | .760 |
|  | BPA | .181 | 13 | .200^*^ | .947 | 13 | .558 |
|  | BPA+BSHX | .129 | 14 | .200^*^ | .948 | 14 | .523 |
|  | BPA+BSHX+*hsp-16.2* RNAi | .171 | 15 | .200^*^ | .963 | 15 | .742 |
| *. This is a lower bound of the true significance. | | | | | | | |
| a. Lilliefors Significance Correction | | | | | | | |

# Control VS BPA

| **Group Statistics** | | | | | |
| --- | --- | --- | --- | --- | --- |
|  | Group | N | Mean | Std. Deviation | Std. Error Mean |
| Brood size | Control | 7 | 172.4286 | 42.75846 | 16.16118 |
|  | BPA | 13 | 82.1538 | 22.72241 | 6.30206 |

| **Independent Samples Test** | | | | | | | | | | |
| --- | --- | --- | --- | --- | --- | --- | --- | --- | --- | --- |
|  | | Levene's Test for Equality of Variances | | t-test for Equality of Means | | | | | | |
|  |  | F | Sig. | t | df | Sig. (2-tailed) | Mean Difference | Std. Error Difference | 95% Confidence Interval of the Difference | |
|  |  |  |  |  |  |  |  |  | Lower | Upper |
| Brood size | Equal variances assumed | 2.047 | .170 | 6.236 | 18 | .000 | 90.27473 | 14.47722 | 59.85922 | 120.69023 |
|  | Equal variances not assumed |  |  | 5.204 | 7.872 | .001 | 90.27473 | 17.34646 | 50.16063 | 130.38882 |

# BPA VS BPA+BSHX

| **Group Statistics** | | | | | |
| --- | --- | --- | --- | --- | --- |
|  | Group | N | Mean | Std. Deviation | Std. Error Mean |
| Brood size | BPA | 13 | 82.1538 | 22.72241 | 6.30206 |
|  | BPA+BSHX | 14 | 115.6429 | 33.36651 | 8.91758 |

| **Independent Samples Test** | | | | | | | | | | |
| --- | --- | --- | --- | --- | --- | --- | --- | --- | --- | --- |
|  | | Levene's Test for Equality of Variances | | t-test for Equality of Means | | | | | | |
|  |  | F | Sig. | t | df | Sig. (2-tailed) | Mean Difference | Std. Error Difference | 95% Confidence Interval of the Difference | |
|  |  |  |  |  |  |  |  |  | Lower | Upper |
| Brood size | Equal variances assumed | 2.071 | .163 | -3.024 | 25 | .006 | -33.48901 | 11.07478 | -56.29794 | -10.68008 |
|  | Equal variances not assumed |  |  | -3.067 | 23.010 | .005 | -33.48901 | 10.91967 | -56.07752 | -10.90050 |

1. BPA+BSHX VS BPA+BSHX+*hsp-16.2* RNAi

| **Group Statistics** | | | | | |
| --- | --- | --- | --- | --- | --- |
|  | Group | N | Mean | Std. Deviation | Std. Error Mean |
| Brood size | BPA+BSHX | 14 | 115.6429 | 33.36651 | 8.91758 |
|  | BPA+BSHX+*hsp-16.2* RNAi | 15 | 78.8000 | 38.65267 | 9.98008 |

| **Independent Samples Test** | | | | | | | | | | |
| --- | --- | --- | --- | --- | --- | --- | --- | --- | --- | --- |
|  | | Levene's Test for Equality of Variances | | t-test for Equality of Means | | | | | | |
|  |  | F | Sig. | t | df | Sig. (2-tailed) | Mean Difference | Std. Error Difference | 95% Confidence Interval of the Difference | |
|  |  |  |  |  |  |  |  |  | Lower | Upper |
| Brood size | Equal variances assumed | .768 | .389 | 2.738 | 27 | .011 | 36.84286 | 13.45381 | 9.23791 | 64.44780 |
|  | Equal variances not assumed |  |  | 2.753 | 26.849 | .010 | 36.84286 | 13.38376 | 9.37440 | 64.31132 |

**Fig. 4 B**

| **Tests of Normality** | | | | | | | |
| --- | --- | --- | --- | --- | --- | --- | --- |
|  | Group | Kolmogorov-Smirnov^a^ | | | Shapiro-Wilk | | |
|  |  | Statistic | df | Sig. | Statistic | df | Sig. |
| Brood size | Control | .244 | 10 | .092 | .881 | 10 | .134 |
|  | BPA | .108 | 13 | .200^*^ | .975 | 13 | .947 |
|  | BPA+BSHX | .124 | 14 | .200^*^ | .968 | 14 | .844 |
|  | BPA+BSHX+*hsp-16.2* RNAi | .272 | 13 | .009 | .872 | 13 | .056 |
| *. This is a lower bound of the true significance. | | | | | | | |
| a. Lilliefors Significance Correction | | | | | | | |

1. Control VS BPA

| **Group Statistics** | | | | | |
| --- | --- | --- | --- | --- | --- |
|  | Group | N | Mean | Std. Deviation | Std. Error Mean |
| Brood size | Control | 10 | 179.8000 | 34.54723 | 10.92479 |
|  | BPA | 13 | 76.9231 | 17.72222 | 4.91526 |

| **Independent Samples Test** | | | | | | | | | | |
| --- | --- | --- | --- | --- | --- | --- | --- | --- | --- | --- |
|  | | Levene's Test for Equality of Variances | | t-test for Equality of Means | | | | | | |
|  |  | F | Sig. | t | df | Sig. (2-tailed) | Mean Difference | Std. Error Difference | 95% Confidence Interval of the Difference | |
|  |  |  |  |  |  |  |  |  | Lower | Upper |
| Brood size | Equal variances assumed | 3.501 | .075 | 9.305 | 21 | .000 | 102.87692 | 11.05666 | 79.88333 | 125.87052 |
|  | Equal variances not assumed |  |  | 8.588 | 12.624 | .000 | 102.87692 | 11.97960 | 76.91809 | 128.83575 |

# BPA VS BPA+BSHX

| **Group Statistics** | | | | | |
| --- | --- | --- | --- | --- | --- |
|  | Group | N | Mean | Std. Deviation | Std. Error Mean |
| Brood size | BPA | 13 | 76.9231 | 17.72222 | 4.91526 |
|  | BPA+BSHX | 14 | 129.6429 | 33.90622 | 9.06182 |

| **Independent Samples Test** | | | | | | | | | | |
| --- | --- | --- | --- | --- | --- | --- | --- | --- | --- | --- |
|  | | Levene's Test for Equality of Variances | | t-test for Equality of Means | | | | | | |
|  |  | F | Sig. | t | df | Sig. (2-tailed) | Mean Difference | Std. Error Difference | 95% Confidence Interval of the Difference | |
|  |  |  |  |  |  |  |  |  | Lower | Upper |
| Brood size | Equal variances assumed | 5.756 | .024 | -5.003 | 25 | .000 | -52.71978 | 10.53807 | -74.42334 | -31.01622 |
|  | Equal variances not assumed |  |  | -5.114 | 19.908 | .000 | -52.71978 | 10.30904 | -74.23044 | -31.20912 |

1. BPA+BSHX VS BPA+BSHX+*hsp-16.2* RNAi

| **Group Statistics** | | | | | |
| --- | --- | --- | --- | --- | --- |
|  | Group | N | Mean | Std. Deviation | Std. Error Mean |
| Brood size | BPA+BSHX | 14 | 129.6429 | 33.90622 | 9.06182 |
|  | BPA+BSHX+*hsp-16.2* RNAi | 13 | 66.1538 | 27.59482 | 7.65343 |

| **Independent Samples Test** | | | | | | | | | | |
| --- | --- | --- | --- | --- | --- | --- | --- | --- | --- | --- |
|  | | Levene's Test for Equality of Variances | | t-test for Equality of Means | | | | | | |
|  |  | F | Sig. | t | df | Sig. (2-tailed) | Mean Difference | Std. Error Difference | 95% Confidence Interval of the Difference | |
|  |  |  |  |  |  |  |  |  | Lower | Upper |
| Brood size | Equal variances assumed | .639 | .432 | 5.311 | 25 | .000 | 63.48901 | 11.95448 | 38.86830 | 88.10972 |
|  | Equal variances not assumed |  |  | 5.353 | 24.601 | .000 | 63.48901 | 11.86135 | 39.03998 | 87.93804 |

**Fig. 4 C**

| **Tests of Normality** | | | | | | | |
| --- | --- | --- | --- | --- | --- | --- | --- |
|  | Group | Kolmogorov-Smirnov^a^ | | | Shapiro-Wilk | | |
|  |  | Statistic | df | Sig. | Statistic | df | Sig. |
| Brood size | Control | .203 | 9 | .200^*^ | .864 | 9 | .107 |
|  | BPA | .134 | 14 | .200^*^ | .953 | 14 | .604 |
|  | BPA+BSHX | .104 | 15 | .200^*^ | .970 | 15 | .851 |
|  | BPA+BSHX+*hsp-16.2* RNAi | .188 | 12 | .200^*^ | .935 | 12 | .436 |
| *. This is a lower bound of the true significance. | | | | | | | |
| a. Lilliefors Significance Correction | | | | | | | |

# Control VS BPA

| **Group Statistics** | | | | | |
| --- | --- | --- | --- | --- | --- |
|  | Group | N | Mean | Std. Deviation | Std. Error Mean |
| Brood size | Control | 9 | 208.2222 | 17.45550 | 5.81850 |
|  | BPA | 14 | 112.4286 | 22.92367 | 6.12661 |

| **Independent Samples Test** | | | | | | | | | | |
| --- | --- | --- | --- | --- | --- | --- | --- | --- | --- | --- |
|  | | Levene's Test for Equality of Variances | | t-test for Equality of Means | | | | | | |
|  |  | F | Sig. | t | df | Sig. (2-tailed) | Mean Difference | Std. Error Difference | 95% Confidence Interval of the Difference | |
|  |  |  |  |  |  |  |  |  | Lower | Upper |
| VAR00002 | Equal variances assumed | 1.175 | .291 | 10.672 | 21 | .000 | 95.79365 | 8.97605 | 77.12694 | 114.46036 |
|  | Equal variances not assumed |  |  | 11.337 | 20.253 | .000 | 95.79365 | 8.44928 | 78.18287 | 113.40443 |

# BPA VS BPA+BSHX

| **Group Statistics** | | | | | |
| --- | --- | --- | --- | --- | --- |
|  | Group | N | Mean | Std. Deviation | Std. Error Mean |
| Brood size | BPA | 14 | 112.4286 | 22.92367 | 6.12661 |
|  | BPA+BSHX | 15 | 169.0667 | 32.18133 | 8.30918 |

| **Independent Samples Test** | | | | | | | | | | |
| --- | --- | --- | --- | --- | --- | --- | --- | --- | --- | --- |
|  | | Levene's Test for Equality of Variances | | t-test for Equality of Means | | | | | | |
|  |  | F | Sig. | t | df | Sig. (2-tailed) | Mean Difference | Std. Error Difference | 95% Confidence Interval of the Difference | |
|  |  |  |  |  |  |  |  |  | Lower | Upper |
| Brood size | Equal variances assumed | .981 | .331 | -5.423 | 27 | .000 | -56.63810 | 10.44496 | -78.06937 | -35.20682 |
|  | Equal variances not assumed |  |  | -5.486 | 25.306 | .000 | -56.63810 | 10.32366 | -77.88705 | -35.38914 |

1. BPA+BSHX VS BPA+BSHX+*hsp-16.2* RNAi

| **Group Statistics** | | | | | |
| --- | --- | --- | --- | --- | --- |
|  | Group | N | Mean | Std. Deviation | Std. Error Mean |
| Brood size | BPA+BSHX | 15 | 169.0667 | 32.18133 | 8.30918 |
|  | BPA+BSHX+*hsp-16.2* RNAi | 12 | 153.5000 | 35.25105 | 10.17610 |

| **Independent Samples Test** | | | | | | | | | | |
| --- | --- | --- | --- | --- | --- | --- | --- | --- | --- | --- |
|  | | Levene's Test for Equality of Variances | | t-test for Equality of Means | | | | | | |
|  |  | F | Sig. | t | df | Sig. (2-tailed) | Mean Difference | Std. Error Difference | 95% Confidence Interval of the Difference | |
|  |  |  |  |  |  |  |  |  | Lower | Upper |
| Brood size | Equal variances assumed | .000 | .985 | 1.197 | 25 | .242 | 15.56667 | 13.00029 | -11.20794 | 42.34127 |
|  | Equal variances not assumed |  |  | 1.185 | 22.648 | .248 | 15.56667 | 13.13756 | -11.63386 | 42.76720 |

**Fig. 4 D**

| **Means and Medians for Survival Time** | | | | | | | | |
| --- | --- | --- | --- | --- | --- | --- | --- | --- |
| Group | Mean^a^ | | | | Median | | | |
|  | Estimate | Std. Error | 95% Confidence Interval | | Estimate | Std. Error | 95% Confidence Interval | |
|  |  |  | Lower Bound | Upper Bound |  |  | Lower Bound | Upper Bound |
| Control | 13.539 | .279 | 12.992 | 14.087 | 14.000 | .195 | 13.618 | 14.382 |
| BPA | 10.417 | .279 | 9.870 | 10.964 | 11.000 | .210 | 10.589 | 11.411 |
| BPA+BSHX | 12.245 | .285 | 11.687 | 12.803 | 12.000 | .291 | 11.431 | 12.569 |
| BPA+BSHX+*hsp-16.2* RNAi | 11.143 | .170 | 10.810 | 11.476 | 11.000 | .183 | 10.641 | 11.359 |
| Overall | 11.867 | .153 | 11.568 | 12.167 | 12.000 | .136 | 11.733 | 12.267 |
| a. Estimation is limited to the largest survival time if it is censored. | | | | | | | | |

| **Pairwise Comparisons** | | | | | | | | | |
| --- | --- | --- | --- | --- | --- | --- | --- | --- | --- |
|  | Group | Control | | BPA | | BPA+BSHX | | BPA+BSHX+*hsp-16.2* RNAi | |
|  |  | Chi-Square | Sig. | Chi-Square | Sig. | Chi-Square | Sig. | Chi-Square | Sig. |
| Log Rank (Mantel-Cox) | Control |  |  | 55.172 | .000 | 8.382 | .004 | 46.665 | .000 |
|  | BPA | 55.172 | .000 |  |  | 21.981 | .000 | 2.593 | .107 |
|  | BPA+BSHX | 8.382 | .004 | 21.981 | .000 |  |  | 13.247 | .000 |
|  | BPA+BSHX+*hsp-16.2* RNAi | 46.665 | .000 | 2.593 | .107 | 13.247 | .000 |  |  |

**Fig. 4 E**

| **Means and Medians for Survival Time** | | | | | | | | |
| --- | --- | --- | --- | --- | --- | --- | --- | --- |
| Group | Mean^a^ | | | | Median | | | |
|  | Estimate | Std. Error | 95% Confidence Interval | | Estimate | Std. Error | 95% Confidence Interval | |
|  |  |  | Lower Bound | Upper Bound |  |  | Lower Bound | Upper Bound |
| Control | 11.814 | .288 | 11.250 | 12.378 | 11.000 | .307 | 10.397 | 11.603 |
| BPA | 9.093 | .270 | 8.564 | 9.622 | 10.000 | .194 | 9.621 | 10.379 |
| BPA+BSHX | 11.219 | .310 | 10.610 | 11.827 | 12.000 | .102 | 11.800 | 12.200 |
| BPA+BSHX+*hsp-16.2* RNAi | 9.821 | .243 | 9.345 | 10.298 | 11.000 | .000 | . | . |
| Overall | 10.461 | .158 | 10.150 | 10.771 | 11.000 | .108 | 10.789 | 11.211 |
| a. Estimation is limited to the largest survival time if it is censored. | | | | | | | | |

| **Pairwise Comparisons** | | | | | | | | | |
| --- | --- | --- | --- | --- | --- | --- | --- | --- | --- |
|  | Group | Control | | BPA | | BPA+BSHX | | BPA+BSHX+*hsp-16.2* RNAi | |
|  |  | Chi-Square | Sig. | Chi-Square | Sig. | Chi-Square | Sig. | Chi-Square | Sig. |
| Log Rank (Mantel-Cox) | Control |  |  | 41.901 | .000 | .695 | .405 | 26.813 | .000 |
|  | BPA | 41.901 | .000 |  |  | 43.563 | .000 | 5.238 | .022 |
|  | BPA+BSHX | .695 | .405 | 43.563 | .000 |  |  | 31.515 | .000 |
|  | BPA+BSHX+*hsp-16.2* RNAi | 26.813 | .000 | 5.238 | .022 | 31.515 | .000 |  |  |

**Fig. 4 F**

| **Means and Medians for Survival Time** | | | | | | | | |
| --- | --- | --- | --- | --- | --- | --- | --- | --- |
| Group | Mean^a^ | | | | Median | | | |
|  | Estimate | Std. Error | 95% Confidence Interval | | Estimate | Std. Error | 95% Confidence Interval | |
|  |  |  | Lower Bound | Upper Bound |  |  | Lower Bound | Upper Bound |
| Control | 12.811 | .307 | 12.210 | 13.412 | 13.000 | .494 | 12.033 | 13.967 |
| BPA | 8.043 | .326 | 7.403 | 8.682 | 7.000 | .426 | 6.165 | 7.835 |
| BPA+BSHX | 12.085 | .301 | 11.495 | 12.675 | 12.000 | .278 | 11.456 | 12.544 |
| BPA+BSHX+*hsp-16.2* RNAi | 10.060 | .188 | 9.691 | 10.429 | 10.000 | .171 | 9.666 | 10.334 |
| Overall | 10.762 | .193 | 10.384 | 11.141 | 11.000 | .214 | 10.581 | 11.419 |
| a. Estimation is limited to the largest survival time if it is censored. | | | | | | | | |

| **Pairwise Comparisons** | | | | | | | | | |
| --- | --- | --- | --- | --- | --- | --- | --- | --- | --- |
|  | Group | Control | | BPA | | BPA+BSHX | | BPA+BSHX+*hsp-16.2* RNAi | |
|  |  | Chi-Square | Sig. | Chi-Square | Sig. | Chi-Square | Sig. | Chi-Square | Sig. |
| Log Rank (Mantel-Cox) | Control |  |  | 70.970 | .000 | 2.898 | .089 | 50.896 | .000 |
|  | BPA | 70.970 | .000 |  |  | 54.087 | .000 | 14.382 | .000 |
|  | BPA+BSHX | 2.898 | .089 | 54.087 | .000 |  |  | 30.231 | .000 |
|  | BPA+BSHX+*hsp-16.2* RNAi | 50.896 | .000 | 14.382 | .000 | 30.231 | .000 |  |  |
