# Supplementary material for: BuShen HuoXue decoction improves fertility through intestinal hsp-16.2-mediated heat-shock signaling pathway in Caenorhabditis elegans
Source: Front Pharmacol. 2023 Jun 2;14:1210701. doi: 10.3389/fphar.2023.1210701 (PMC10272376; doi:10.3389/fphar.2023.1210701)
Supplement: Supplementary file 13 [file Table6.DOCX]

**Fig. 2 B.**

| **Tests of Normality** | | | | | | | | |
| --- | --- | --- | --- | --- | --- | --- | --- | --- |
|  | Group | | Kolmogorov-Smirnov^a^ | | | Shapiro-Wilk | | |
|  |  |  | Statistic | df | Sig. | Statistic | df | Sig. |
| Brood size | Control | | .178 | 19 | .114 | .919 | 19 | .108 |
|  | BPA  (µg/mL) | 100 | .123 | 19 | .200^*^ | .946 | 19 | .344 |
|  |  | 125 | .139 | 19 | .200^*^ | .916 | 19 | .097 |
|  |  | 150 | .158 | 18 | .200^*^ | .920 | 18 | .129 |
|  |  | 175 | .121 | 17 | .200^*^ | .948 | 17 | .425 |
| *. This is a lower bound of the true significance. | | | | | | | | |
| a. Lilliefors Significance Correction | | | | | | | | |

| **ANOVA** | | | | | |
| --- | --- | --- | --- | --- | --- |
| Broodsize | | | | | |
|  | Sum of Squares | df | Mean Square | F | Sig. |
| Between Groups | 53273.645 | 4 | 13318.411 | 4.269 | .003 |
| Within Groups | 271447.790 | 87 | 3120.090 |  |  |
| Total | 324721.435 | 91 |  |  |  |

| **Multiple Comparisons** | | | | | | |
| --- | --- | --- | --- | --- | --- | --- |
| Dependent Variable: Broodsize | | | | | | |
| LSD | | | | | | |
| (I)  Group | (J)  Group | Mean Difference (I-J) | Std. Error | Sig. | 95% Confidence Interval | |
|  |  |  |  |  | Lower Bound | Upper Bound |
| Control | BPA(100 μg/mL) | 17.89474 | 18.12265 | .326 | -18.1260 | 53.9155 |
|  | BPA(125 μg/mL) | 6.05263 | 18.12265 | .739 | -29.9681 | 42.0734 |
|  | BPA(150 μg/mL) | 47.63743^*^ | 18.37263 | .011 | 11.1198 | 84.1550 |
|  | BPA(175 μg/mL) | 62.93808^*^ | 18.64805 | .001 | 25.8731 | 100.0031 |
| BPA(100 μg/mL) | Control | -17.89474 | 18.12265 | .326 | -53.9155 | 18.1260 |
|  | BPA(125 μg/mL) | -11.84211 | 18.12265 | .515 | -47.8628 | 24.1786 |
|  | BPA(150 μg/mL) | 29.74269 | 18.37263 | .109 | -6.7749 | 66.2603 |
|  | BPA(175 μg/mL) | 45.04334^*^ | 18.64805 | .018 | 7.9783 | 82.1084 |
| BPA(125 μg/mL) | Control | -6.05263 | 18.12265 | .739 | -42.0734 | 29.9681 |
|  | BPA(100 μg/mL) | 11.84211 | 18.12265 | .515 | -24.1786 | 47.8628 |
|  | BPA(150 μg/mL) | 41.58480^*^ | 18.37263 | .026 | 5.0672 | 78.1024 |
|  | BPA(175 μg/mL) | 56.88545^*^ | 18.64805 | .003 | 19.8204 | 93.9505 |
| BPA(150 μg/mL) | Control | -47.63743^*^ | 18.37263 | .011 | -84.1550 | -11.1198 |
|  | BPA(100 μg/mL) | -29.74269 | 18.37263 | .109 | -66.2603 | 6.7749 |
|  | BPA(125 μg/mL) | -41.58480^*^ | 18.37263 | .026 | -78.1024 | -5.0672 |
|  | BPA(175 μg/mL) | 15.30065 | 18.89108 | .420 | -22.2474 | 52.8487 |
| BPA(175 μg/mL) | Control | -62.93808^*^ | 18.64805 | .001 | -100.0031 | -25.8731 |
|  | BPA(100 μg/mL) | -45.04334^*^ | 18.64805 | .018 | -82.1084 | -7.9783 |
|  | BPA(125 μg/mL) | -56.88545^*^ | 18.64805 | .003 | -93.9505 | -19.8204 |
|  | BPA(150 μg/mL) | -15.30065 | 18.89108 | .420 | -52.8487 | 22.2474 |
| *. The mean difference is significant at the 0.05 level. | | | | | | |

**Fig. 2 C.**

| **Tests of Normality** | | | | | | | | |
| --- | --- | --- | --- | --- | --- | --- | --- | --- |
|  | Group | | Kolmogorov-Smirnov^a^ | | | Shapiro-Wilk | | |
|  |  |  | Statistic | df | Sig. | Statistic | df | Sig. |
| Brood size | Control | | .108 | 19 | .200^*^ | .954 | 19 | .456 |
|  | BPA | | .181 | 12 | .200^*^ | .943 | 12 | .539 |
|  | BPA+BSHX  (mg/mL) | 31.25 | .153 | 17 | .200^*^ | .896 | 17 | .058 |
|  |  | 62.5 | .173 | 18 | .160 | .943 | 18 | .321 |
|  |  | 125 | .120 | 15 | .200^*^ | .957 | 15 | .633 |
| *. This is a lower bound of the true significance. | | | | | | | | |
| a. Lilliefors Significance Correction | | | | | | | | |

# Control VS BPA

| **Group Statistics** | | | | | |
| --- | --- | --- | --- | --- | --- |
|  | Group | N | Mean | Std. Deviation | Std. Error Mean |
| Brood size | Control | 19 | 215.3684 | 33.28031 | 7.63503 |
|  | BPA | 12 | 151.0833 | 19.92923 | 5.75307 |

| **Independent Samples Test** | | | | | | | | | | |
| --- | --- | --- | --- | --- | --- | --- | --- | --- | --- | --- |
|  | | Levene's Test for Equality of Variances | | t-test for Equality of Means | | | | | | |
|  |  | F | Sig. | t | df | Sig. (2-tailed) | Mean Difference | Std. Error Difference | 95% Confidence Interval of the Difference | |
|  |  |  |  |  |  |  |  |  | Lower | Upper |
| Brood size | Equal variances assumed | 4.324 | .047 | 6.022 | 29 | .000 | 64.28509 | 10.67494 | 42.45239 | 86.11778 |
|  | Equal variances not assumed |  |  | 6.724 | 28.964 | .000 | 64.28509 | 9.55989 | 44.73186 | 83.83832 |

# BPA VS BPA+BSHX (31.25 mg/mL、62.5 mg/mL、125 mg/mL)

| **ANOVA** | | | | | |
| --- | --- | --- | --- | --- | --- |
| Broodsize | | | | | |
|  | Sum of Squares | df | Mean Square | F | Sig. |
| Between Groups | 25597.707 | 3 | 8532.569 | 6.559 | .001 |
| Within Groups | 76757.372 | 59 | 1300.972 |  |  |
| Total | 102355.079 | 62 |  |  |  |

| **Multiple Comparisons** | | | | | | | |
| --- | --- | --- | --- | --- | --- | --- | --- |
| Dependent Variable: Broodsize | | | | | | | |
|  | (I) Group | (J) Group | Mean Difference (I-J) | Std. Error | Sig. | 95% Confidence Interval | |
|  |  |  |  |  |  | Lower Bound | Upper Bound |
| LSD | BPA | BPA+31.25 mg/mL BSHX | -14.02778 | 13.44212 | .301 | -40.9254 | 12.8698 |
|  |  | BPA+62.5 mg/mL BSHX | -46.30556^*^ | 13.44212 | .001 | -73.2032 | -19.4079 |
|  |  | BPA+125 mg/mL BSHX | 3.88333 | 13.96946 | .782 | -24.0695 | 31.8362 |
|  | BPA+31.25 mg/mL BSHX | BPA | 14.02778 | 13.44212 | .301 | -12.8698 | 40.9254 |
|  |  | BPA+62.5 mg/mL BSHX | -32.27778^*^ | 12.02300 | .009 | -56.3357 | -8.2198 |
|  |  | BPA+125 mg/mL BSHX | 17.91111 | 12.60983 | .161 | -7.3211 | 43.1433 |
|  | BPA+62.5 mg/mL BSHX | BPA | 46.30556^*^ | 13.44212 | .001 | 19.4079 | 73.2032 |
|  |  | BPA+31.25 mg/mL BSHX | 32.27778^*^ | 12.02300 | .009 | 8.2198 | 56.3357 |
|  |  | BPA+125 mg/mL BSHX | 50.18889^*^ | 12.60983 | .000 | 24.9567 | 75.4211 |
|  | BPA+125 mg/mL BSHX | BPA | -3.88333 | 13.96946 | .782 | -31.8362 | 24.0695 |
|  |  | BPA+31.25 mg/mL BSHX | -17.91111 | 12.60983 | .161 | -43.1433 | 7.3211 |
|  |  | BPA+62.5 mg/mL BSHX | -50.18889^*^ | 12.60983 | .000 | -75.4211 | -24.9567 |
| Dunnett t (2-sided)^b^ | BPA+31.25 mg/mL BSHX | BPA | 14.02778 | 13.44212 | .574 | -18.1201 | 46.1756 |
|  | BPA+31.25 mg/mL BSHX | BPA | 46.30556^*^ | 13.44212 | .003 | 14.1577 | 78.4534 |
|  | BPA+125 mg/mL BSHX | BPA | -3.88333 | 13.96946 | .983 | -37.2924 | 29.5257 |
| *. The mean difference is significant at the 0.05 level. | | | | | | | |
| b. Dunnett t-tests treat one group as a control, and compare all other groups against it. | | | | | | | |

**Fig. 2 D**

| **Tests of Normality** | | | | | | | |
| --- | --- | --- | --- | --- | --- | --- | --- |
|  | Group | Kolmogorov-Smirnov^a^ | | | Shapiro-Wilk | | |
|  |  | Statistic | df | Sig. | Statistic | df | Sig. |
|  | Control | .090 | 15 | .200^*^ | .984 | 15 | .990 |
|  | BPA | .172 | 11 | .200^*^ | .917 | 11 | .296 |
| Dosing duration | 24 h | .172 | 12 | .200^*^ | .939 | 12 | .491 |
|  | 48 h | .157 | 14 | .200^*^ | .938 | 14 | .391 |
|  | 72 h | .207 | 12 | .164 | .924 | 12 | .319 |
| *. This is a lower bound of the true significance. | | | | | | | |
| a. Lilliefors Significance Correction | | | | | | | |

# Control VS BPA

| **Group Statistics** | | | | | |
| --- | --- | --- | --- | --- | --- |
|  | Group | N | Mean | Std. Deviation | Std. Error Mean |
|  | Control | 15 | 255.4000 | 28.23827 | 7.29109 |
|  | BPA | 11 | 133.7273 | 68.90441 | 20.77546 |

| **Independent Samples Test** | | | | | | | | | | |
| --- | --- | --- | --- | --- | --- | --- | --- | --- | --- | --- |
|  | | Levene's Test for Equality of Variances | | t-test for Equality of Means | | | | | | |
|  |  | F | Sig. | t | df | Sig. (2-tailed) | Mean Difference | Std. Error Difference | 95% Confidence Interval of the Difference | |
|  |  |  |  |  |  |  |  |  | Lower | Upper |
| Dosing duration | Equal variances assumed | 12.375 | .002 | 6.201 | 24 | .000 | 121.67273 | 19.62197 | 81.17497 | 162.17049 |
|  | Equal variances not assumed |  |  | 5.526 | 12.480 | .000 | 121.67273 | 22.01772 | 73.90398 | 169.44147 |

# BPA VS BSHX (24h、48h、72h)

| **ANOVA** | | | | | |
| --- | --- | --- | --- | --- | --- |
| VAR00002 | | | | | |
|  | Sum of Squares | df | Mean Square | F | Sig. |
| Between Groups | 53346.218 | 3 | 17782.073 | 5.783 | .002 |
| Within Groups | 138368.598 | 45 | 3074.858 |  |  |
| Total | 191714.816 | 48 |  |  |  |

| **Multiple Comparisons** | | | | | | | |
| --- | --- | --- | --- | --- | --- | --- | --- |
| Dependent Variable: Broodsize | | | | | | | |
|  | (I) Group | (J) Group | Mean Difference (I-J) | Std. Error | Sig. | 95% Confidence Interval | |
|  |  |  |  |  |  | Lower Bound | Upper Bound |
| LSD | BPA | BPA+BSHX(24h) | -93.27273^*^ | 23.14672 | .000 | -139.8926 | -46.6528 |
|  |  | BPA+BSHX(48h) | -57.77273^*^ | 22.34201 | .013 | -102.7718 | -12.7736 |
|  |  | BPA+BSHX(72h) | -69.35606^*^ | 23.14672 | .004 | -115.9759 | -22.7362 |
|  | BPA+BSHX(24h) | BPA | 93.27273^*^ | 23.14672 | .000 | 46.6528 | 139.8926 |
|  |  | BPA+BSHX(48h) | 35.50000 | 21.81446 | .111 | -8.4366 | 79.4366 |
|  |  | BPA+BSHX(72h) | 23.91667 | 22.63794 | .296 | -21.6785 | 69.5118 |
|  | BPA+BSHX(48h) | BPA | 57.77273^*^ | 22.34201 | .013 | 12.7736 | 102.7718 |
|  |  | BPA+BSHX(24h) | -35.50000 | 21.81446 | .111 | -79.4366 | 8.4366 |
|  |  | BPA+BSHX(72h) | -11.58333 | 21.81446 | .598 | -55.5199 | 32.3533 |
|  | BPA+BSHX(72h) | BPA | 69.35606^*^ | 23.14672 | .004 | 22.7362 | 115.9759 |
|  |  | BPA+BSHX(24h) | -23.91667 | 22.63794 | .296 | -69.5118 | 21.6785 |
|  |  | BPA+BSHX(48h) | 11.58333 | 21.81446 | .598 | -32.3533 | 55.5199 |
| Dunnett t (2-sided)^b^ | BPA+BSHX(24h) | BPA | 93.27273^*^ | 23.14672 | .001 | 37.1780 | 149.3675 |
|  | BPA+BSHX(48h) | BPA | 57.77273^*^ | 22.34201 | .034 | 3.6282 | 111.9173 |
|  | BPA+BSHX(72h) | BPA | 69.35606^*^ | 23.14672 | .012 | 13.2613 | 125.4508 |
| *. The mean difference is significant at the 0.05 level. | | | | | | | |
| b. Dunnett t-tests treat one group as a control, and compare all other groups against it. | | | | | | | |

**Fig. 2 E**

| **Tests of Normality** | | | | | | | |
| --- | --- | --- | --- | --- | --- | --- | --- |
|  | Group | Kolmogorov-Smirnov^a^ | | | Shapiro-Wilk | | |
|  |  | Statistic | df | Sig. | Statistic | df | Sig. |
| Relative fluoresence intensity | Control | .184 | 15 | .185 | .892 | 15 | .072 |
|  | BPA | .155 | 24 | .143 | .943 | 24 | .190 |
|  | BPA+BSHX | .232 | 19 | .008 | .846 | 19 | .006 |
| a. Lilliefors Significance Correction | | | | | | | |

| **Hypothesis Test Summary** | | | | |
| --- | --- | --- | --- | --- |
|  | Null Hypothesis | Test | Sig.^b,c^ | Decision |
| Control | The distribution of relative fluoresence intensity ： is the same across categories of group. | Independent-Samples Mann-Whitney U Test | .^a^ | Unable to compute. |
| BPA | The distribution of relative fluoresence intensity： is the same across categories of group. | Independent-Samples Kruskal-Wallis Test | .018 | Reject the null hypothesis. |
| a. The group field does not have exactly two values. | | | | |
| b. The significance level is .050. | | | | |
| c. Asymptotic significance is displayed. | | | | |

| **Independent-Samples Kruskal-Wallis Test Summary** | |
| --- | --- |
| Total N | 58 |
| Test Statistic | 8.045^a^ |
| Degree Of Freedom | 2 |
| Asymptotic Sig.(2-sided test) | .018 |
| a. The test statistic is adjusted for ties. | |

| **Pairwise Comparisons of Group** | | | | | |
| --- | --- | --- | --- | --- | --- |
| Sample 1-Sample 2 | Test Statistic | Std. Error | Std. Test Statistic | Sig. | Adj. Sig.^a^ |
| BPA—BPA+BSHX | -11.044 | 5.186 | -2.130 | .033 | .100 |
| BPA—Control | 14.367 | 5.558 | 2.585 | .010 | .029 |
| BPA+BSHX—Control | 3.323 | 5.833 | .570 | .569 | 1.000 |
| Each row tests the null hypothesis that the Sample 1 and Sample 2 distributions are the same.  Asymptotic significances (2-sided tests) are displayed. The significance level is .050. | | | | | |
| a. Significance values have been adjusted by the Bonferroni correction for multiple tests. | | | | | |

**Fig. 2 F**

| **Tests of Normality** | | | | | | | |
| --- | --- | --- | --- | --- | --- | --- | --- |
|  | Group | Kolmogorov-Smirnov^a^ | | | Shapiro-Wilk | | |
|  |  | Statistic | df | Sig. | Statistic | df | Sig. |
| Apoptotic cells | Control | .182 | 13 | .200^*^ | .936 | 13 | .404 |
|  | BPA | .200 | 16 | .086 | .933 | 16 | .272 |
|  | BPA+BSHX | .239 | 13 | .041 | .870 | 13 | .052 |
| *. This is a lower bound of the true significance. | | | | | | | |
| a. Lilliefors Significance Correction | | | | | | | |

# Control VS BPA

| **Group Statistics** | | | | | |
| --- | --- | --- | --- | --- | --- |
|  | Group | N | Mean | Std. Deviation | Std. Error Mean |
| Apoptotic cells | Control | 12 | 2.7692 | 1.48064 | .41066 |
|  | BPA | 16 | 7.0625 | 1.98221 | .49555 |

| **Independent Samples Test** | | | | | | | | | | |
| --- | --- | --- | --- | --- | --- | --- | --- | --- | --- | --- |
|  | | Levene's Test for Equality of Variances | | t-test for Equality of Means | | | | | | |
|  |  | F | Sig. | t | df | Sig. (2-tailed) | Mean Difference | Std. Error Difference | 95% Confidence Interval of the Difference | |
|  |  |  |  |  |  |  |  |  | Lower | Upper |
| Apoptotic cells | Equal variances assumed | .388 | .539 | -6.471 | 27 | .000 | -4.29327 | .66347 | -5.65459 | -2.93194 |
|  | Equal variances not assumed |  |  | -6.671 | 26.849 | .000 | -4.29327 | .64359 | -5.61416 | -2.97238 |

# BPA VS BPA+BSHX

| **Group Statistics** | | | | | |
| --- | --- | --- | --- | --- | --- |
|  | Group | N | Mean | Std. Deviation | Std. Error Mean |
| Apoptotic cells | BPA | 16 | 7.0625 | 1.98221 | .49555 |
|  | BPA+BSHX | 13 | 5.2308 | 1.96443 | .54483 |

| **Independent Samples Test** | | | | | | | | | | |
| --- | --- | --- | --- | --- | --- | --- | --- | --- | --- | --- |
|  | | Levene's Test for Equality of Variances | | t-test for Equality of Means | | | | | | |
|  |  | F | Sig. | t | df | Sig. (2-tailed) | Mean Difference | Std. Error Difference | 95% Confidence Interval of the Difference | |
|  |  |  |  |  |  |  |  |  | Lower | Upper |
| Apoptotic cells | Equal variances assumed | .018 | .895 | 2.485 | 27 | .019 | 1.83173 | .73720 | .31912 | 3.34434 |
|  | Equal variances not assumed |  |  | 2.487 | 25.891 | .020 | 1.83173 | .73649 | .31755 | 3.34592 |

**Fig. 2 G**

| **Tests of Normality** | | | | | | | |
| --- | --- | --- | --- | --- | --- | --- | --- |
|  | Group | Kolmogorov-Smirnov^a^ | | | Shapiro-Wilk | | |
|  |  | Statistic | df | Sig. | Statistic | df | Sig. |
| Number of Oocytes | Control | .185 | 14 | .200^*^ | .898 | 14 | .106 |
|  | BPA | .264 | 18 | .002 | .864 | 18 | .014 |
|  | BPA+BSHX | .159 | 13 | .200^*^ | .912 | 13 | .195 |
| *. This is a lower bound of the true significance. | | | | | | | |
| a. Lilliefors Significance Correction | | | | | | | |

| **Hypothesis Test Summary** | | | | |
| --- | --- | --- | --- | --- |
|  | Null Hypothesis | Test | Sig.^a,b^ | Decision |
| Control | The distribution of Number of Oocytes is the same across categories of group. | Independent-Samples Kruskal-Wallis Test | .000 | Reject the null hypothesis. |
| a. The significance level is .050. | | | | |
| b. Asymptotic significance is displayed. | | | | |

| **Independent-Samples Kruskal-Wallis Test Summary** | |
| --- | --- |
| Total N | 45 |
| Test Statistic | 16.128^a^ |
| Degree Of Freedom | 2 |
| Asymptotic Sig.(2-sided test) | .000 |
| a. The test statistic is adjusted for ties. | |

| **Pairwise Comparisons of Group** | | | | | |
| --- | --- | --- | --- | --- | --- |
| Sample 1-Sample 2 | Test Statistic | Std. Error | Std. Test Statistic | Sig. | Adj. Sig.^a^ |
| BPA—BPA+BSHX | -13.603 | 4.717 | -2.884 | .004 | .012 |
| BPA—Control | 17.369 | 4.618 | 3.761 | .000 | .001 |
| BPA+BSHX—Control | 3.766 | 4.992 | .755 | .451 | 1.000 |
| Each row tests the null hypothesis that the Sample 1 and Sample 2 distributions are the same.  Asymptotic significances (2-sided tests) are displayed. The significance level is .050. | | | | | |
| a. Significance values have been adjusted by the Bonferroni correction for multiple tests. | | | | | |
