# Supplementary material for: BuShen HuoXue decoction improves fertility through intestinal hsp-16.2-mediated heat-shock signaling pathway in Caenorhabditis elegans
Source: Front Pharmacol. 2023 Jun 2;14:1210701. doi: 10.3389/fphar.2023.1210701 (PMC10272376; doi:10.3389/fphar.2023.1210701)
Supplement: Supplementary file 17 [file Table14.DOCX]

**Fig. 5 A**

1.C*lc-2*

| **Tests of Normality** | | | | | | | |
| --- | --- | --- | --- | --- | --- | --- | --- |
|  | Group | Kolmogorov-Smirnov^a^ | | | Shapiro-Wilk | | |
|  |  | Statistic | df | Sig. | Statistic | df | Sig. |
| *Clc-2* | Control | .242 | 3 | . | .973 | 3 | .683 |
|  | BPA | .295 | 3 | . | .920 | 3 | .452 |
|  | BPA+BSHX | .368 | 3 | . | .791 | 3 | .092 |
| a. Lilliefors Significance Correction | | | | | | | |

- 1. Control VS BPA

| **Group Statistics** | | | | | |
| --- | --- | --- | --- | --- | --- |
|  | Group | N | Mean | Std. Deviation | Std. Error Mean |
| *Clc-2* | Control | 3 | 100.0500 | 3.88325 | 2.24199 |
|  | BPA | 3 | 69.2233 | 7.58459 | 4.37897 |

| **Independent Samples Test** | | | | | | | | | | |
| --- | --- | --- | --- | --- | --- | --- | --- | --- | --- | --- |
|  | | Levene's Test for Equality of Variances | | t-test for Equality of Means | | | | | | |
|  |  | F | Sig. | t | df | Sig. (2-tailed) | Mean Difference | Std. Error Difference | 95% Confidence Interval of the Difference | |
|  |  |  |  |  |  |  |  |  | Lower | Upper |
| *Clc-2* | Equal variances assumed | 1.980 | .232 | 6.266 | 4 | .003 | 30.82667 | 4.91954 | 17.16783 | 44.48550 |
|  | Equal variances not assumed |  |  | 6.266 | 2.981 | .008 | 30.82667 | 4.91954 | 15.11426 | 46.53907 |

1.2 BPA VS BPA+BSHX

| **Group Statistics** | | | | | |
| --- | --- | --- | --- | --- | --- |
|  | Group | N | Mean | Std. Deviation | Std. Error Mean |
| *Clc-2* | BPA | 3 | 69.2233 | 7.58459 | 4.37897 |
|  | BPA+BSHX | 3 | 179.1833 | 23.75382 | 13.71428 |

| **Independent Samples Test** | | | | | | | | | | |
| --- | --- | --- | --- | --- | --- | --- | --- | --- | --- | --- |
|  | | Levene's Test for Equality of Variances | | t-test for Equality of Means | | | | | | |
|  |  | F | Sig. | t | df | Sig. (2-tailed) | Mean Difference | Std. Error Difference | 95% Confidence Interval of the Difference | |
|  |  |  |  |  |  |  |  |  | Lower | Upper |
| *Clc-2* | Equal variances assumed | 6.507 | .063 | -7.638 | 4 | .002 | -109.96000 | 14.39641 | -149.93085 | -69.98915 |
|  | Equal variances not assumed |  |  | -7.638 | 2.404 | .010 | -109.96000 | 14.39641 | -162.92913 | -56.99087 |

2.E*gl-8*

| **Tests of Normality** | | | | | | | |
| --- | --- | --- | --- | --- | --- | --- | --- |
|  | Group | Kolmogorov-Smirnov^a^ | | | Shapiro-Wilk | | |
|  |  | Statistic | df | Sig. | Statistic | df | Sig. |
| *Egl-8* | Control | .289 | 3 | . | .928 | 3 | .480 |
|  | BPA | .367 | 3 | . | .792 | 3 | .096 |
|  | BPA+BSHX | .320 | 3 | . | .883 | 3 | .333 |
| a. Lilliefors Significance Correction | | | | | | | |

2.1 Control VS BPA

| **Group Statistics** | | | | | |
| --- | --- | --- | --- | --- | --- |
|  | Group | N | Mean | Std. Deviation | Std. Error Mean |
| *Egl-8* | Control | 3 | 101.1267 | 18.89585 | 10.90953 |
|  | BPA | 3 | 126.4967 | 35.58360 | 20.54420 |

| **Independent Samples Test** | | | | | | | | | | |
| --- | --- | --- | --- | --- | --- | --- | --- | --- | --- | --- |
|  | | Levene's Test for Equality of Variances | | t-test for Equality of Means | | | | | | |
|  |  | F | Sig. | t | df | Sig. (2-tailed) | Mean Difference | Std. Error Difference | 95% Confidence Interval of the Difference | |
|  |  |  |  |  |  |  |  |  | Lower | Upper |
| *Egl-8* | Equal variances assumed | 2.604 | .182 | -1.091 | 4 | .337 | -25.37000 | 23.26117 | -89.95335 | 39.21335 |
|  | Equal variances not assumed |  |  | -1.091 | 3.045 | .354 | -25.37000 | 23.26117 | -98.78405 | 48.04405 |

2.2 BPA VS BPA+BSHX

| **Group Statistics** | | | | | |
| --- | --- | --- | --- | --- | --- |
|  | Group | N | Mean | Std. Deviation | Std. Error Mean |
| *Egl-8* | BPA | 3 | 126.4967 | 35.58360 | 20.54420 |
|  | BPA+BSHX | 3 | 100.1000 | 46.80735 | 27.02424 |

| **Independent Samples Test** | | | | | | | | | | |
| --- | --- | --- | --- | --- | --- | --- | --- | --- | --- | --- |
|  | | Levene's Test for Equality of Variances | | t-test for Equality of Means | | | | | | |
|  |  | F | Sig. | t | df | Sig. (2-tailed) | Mean Difference | Std. Error Difference | 95% Confidence Interval of the Difference | |
|  |  |  |  |  |  |  |  |  | Lower | Upper |
| *Egl-8* | Equal variances assumed | .445 | .541 | .778 | 4 | .480 | 26.39667 | 33.94663 | -67.85428 | 120.64761 |
|  | Equal variances not assumed |  |  | .778 | 3.733 | .483 | 26.39667 | 33.94663 | -70.57433 | 123.36766 |

3.*Ifb-2*

| **Tests of Normality** | | | | | | | |
| --- | --- | --- | --- | --- | --- | --- | --- |
|  | Group | Kolmogorov-Smirnov^a^ | | | Shapiro-Wilk | | |
|  |  | Statistic | df | Sig. | Statistic | df | Sig. |
| *Ifb-2* | Control | .280 | 3 | . | .938 | 3 | .519 |
|  | BPA | .325 | 3 | . | .876 | 3 | .312 |
|  | BPA+BSHX | .359 | 3 | . | .810 | 3 | .139 |
| a. Lilliefors Significance Correction | | | | | | | |

3.1 Control VS BPA

| **Group Statistics** | | | | | |
| --- | --- | --- | --- | --- | --- |
|  | Group | N | Mean | Std. Deviation | Std. Error Mean |
| *Ifb-2* | Control | 3 | 100.3100 | 9.53014 | 5.50223 |
|  | BPA | 3 | 81.8567 | 12.73823 | 7.35442 |

| **Independent Samples Test** | | | | | | | | | | |
| --- | --- | --- | --- | --- | --- | --- | --- | --- | --- | --- |
|  | | Levene's Test for Equality of Variances | | t-test for Equality of Means | | | | | | |
|  |  | F | Sig. | t | df | Sig. (2-tailed) | Mean Difference | Std. Error Difference | 95% Confidence Interval of the Difference | |
|  |  |  |  |  |  |  |  |  | Lower | Upper |
| *Ifb-2* | Equal variances assumed | .541 | .503 | 2.009 | 4 | .115 | 18.45333 | 9.18488 | -7.04799 | 43.95465 |
|  | Equal variances not assumed |  |  | 2.009 | 3.705 | .121 | 18.45333 | 9.18488 | -7.86965 | 44.77632 |

3.2 BPA VS BPA+BSHX

| **Group Statistics** | | | | | |
| --- | --- | --- | --- | --- | --- |
|  | Group | N | Mean | Std. Deviation | Std. Error Mean |
| *Ifb-2* | BPA | 3 | 81.8567 | 12.73823 | 7.35442 |
|  | BPA+BSHX | 3 | 140.8867 | 6.96098 | 4.01892 |

| **Independent Samples Test** | | | | | | | | | | |
| --- | --- | --- | --- | --- | --- | --- | --- | --- | --- | --- |
|  | | Levene's Test for Equality of Variances | | t-test for Equality of Means | | | | | | |
|  |  | F | Sig. | t | df | Sig. (2-tailed) | Mean Difference | Std. Error Difference | 95% Confidence Interval of the Difference | |
|  |  |  |  |  |  |  |  |  | Lower | Upper |
| *Ifb-2* | Equal variances assumed | 2.050 | .225 | -7.043 | 4 | .002 | -59.03000 | 8.38089 | -82.29907 | -35.76093 |
|  | Equal variances not assumed |  |  | -7.043 | 3.097 | .005 | -59.03000 | 8.38089 | -85.23672 | -32.82328 |

4.*Dlg-1*

| **Tests of Normality** | | | | | | | |
| --- | --- | --- | --- | --- | --- | --- | --- |
|  | CASE_LBL | Kolmogorov-Smirnov^a^ | | | Shapiro-Wilk | | |
|  |  | Statistic | df | Sig. | Statistic | df | Sig. |
| *Dlg-1* | Control | .290 | 3 | . | .926 | 3 | .474 |
|  | BPA | .256 | 3 | . | .961 | 3 | .622 |
|  | BPA+BSHX | .261 | 3 | . | .958 | 3 | .604 |
| a. Lilliefors Significance Correction | | | | | | | |

4.1 Control VS BPA

| **Group Statistics** | | | | | |
| --- | --- | --- | --- | --- | --- |
|  | Group | N | Mean | Std. Deviation | Std. Error Mean |
| *Dlg-1* | Control | 3 | 100.0300 | 2.86794 | 1.65581 |
|  | BPA | 3 | 99.8033 | 3.18717 | 1.84011 |

| **Independent Samples Test** | | | | | | | | | | |
| --- | --- | --- | --- | --- | --- | --- | --- | --- | --- | --- |
|  | | Levene's Test for Equality of Variances | | t-test for Equality of Means | | | | | | |
|  |  | F | Sig. | t | df | Sig. (2-tailed) | Mean Difference | Std. Error Difference | 95% Confidence Interval of the Difference | |
|  |  |  |  |  |  |  |  |  | Lower | Upper |
| *Dlg-1* | Equal variances assumed | .030 | .871 | .092 | 4 | .931 | .22667 | 2.47542 | -6.64620 | 7.09954 |
|  | Equal variances not assumed |  |  | .092 | 3.956 | .931 | .22667 | 2.47542 | -6.67628 | 7.12961 |

4.2 BPA VS BPA+BSHX

| **Group Statistics** | | | | | |
| --- | --- | --- | --- | --- | --- |
|  | CASE_LBL | N | Mean | Std. Deviation | Std. Error Mean |
| *Dlg-1* | BPA | 3 | 99.8033 | 3.18717 | 1.84011 |
|  | BPA+BSHX | 3 | 137.3167 | 14.81817 | 8.55527 |

| **Independent Samples Test** | | | | | | | | | | |
| --- | --- | --- | --- | --- | --- | --- | --- | --- | --- | --- |
|  | | Levene's Test for Equality of Variances | | t-test for Equality of Means | | | | | | |
|  |  | F | Sig. | t | df | Sig. (2-tailed) | Mean Difference | Std. Error Difference | 95% Confidence Interval of the Difference | |
|  |  |  |  |  |  |  |  |  | Lower | Upper |
| dlg_1 | Equal variances assumed | 4.805 | .093 | -4.287 | 4 | .013 | -37.51333 | 8.75093 | -61.80980 | -13.21687 |
|  | Equal variances not assumed |  |  | -4.287 | 2.185 | .043 | -37.51333 | 8.75093 | -72.27438 | -2.75229 |

1. *Act-5*

| **Tests of Normality** | | | | | | | |
| --- | --- | --- | --- | --- | --- | --- | --- |
|  | Group | Kolmogorov-Smirnov^a^ | | | Shapiro-Wilk | | |
|  |  | Statistic | df | Sig. | Statistic | df | Sig. |
| *Act-5* | Control | .304 | 3 | . | .907 | 3 | .409 |
|  | BPA | .223 | 3 | . | .985 | 3 | .764 |
|  | BPA+BSHX | .319 | 3 | . | .886 | 3 | .341 |
| a. Lilliefors Significance Correction | | | | | | | |

- 1. Control VS BPA

| **Group Statistics** | | | | | |
| --- | --- | --- | --- | --- | --- |
|  | Group | N | Mean | Std. Deviation | Std. Error Mean |
| *Act-5* | Control | 3 | 100.0767 | 4.77131 | 2.75472 |
|  | BPA | 3 | 78.8533 | 2.76109 | 1.59412 |

| **Independent Samples Test** | | | | | | | | | | |
| --- | --- | --- | --- | --- | --- | --- | --- | --- | --- | --- |
|  | | Levene's Test for Equality of Variances | | t-test for Equality of Means | | | | | | |
|  |  | F | Sig. | t | df | Sig. (2-tailed) | Mean Difference | Std. Error Difference | 95% Confidence Interval of the Difference | |
|  |  |  |  |  |  |  |  |  | Lower | Upper |
| *Act-5* | Equal variances assumed | 1.500 | .288 | 6.668 | 4 | .003 | 21.22333 | 3.18272 | 12.38669 | 30.05997 |
|  | Equal variances not assumed |  |  | 6.668 | 3.204 | .006 | 21.22333 | 3.18272 | 11.45045 | 30.99622 |

5.2 BPA VS BPA+BSHX

| **Group Statistics** | | | | | |
| --- | --- | --- | --- | --- | --- |
|  | Group | N | Mean | Std. Deviation | Std. Error Mean |
| *Act-5* | BPA | 3 | 78.8533 | 2.76109 | 1.59412 |
|  | BPA+BSHX | 3 | 158.6600 | 11.83672 | 6.83393 |

| **Independent Samples Test** | | | | | | | | | | |
| --- | --- | --- | --- | --- | --- | --- | --- | --- | --- | --- |
|  | | Levene's Test for Equality of Variances | | t-test for Equality of Means | | | | | | |
|  |  | F | Sig. | t | df | Sig. (2-tailed) | Mean Difference | Std. Error Difference | 95% Confidence Interval of the Difference | |
|  |  |  |  |  |  |  |  |  | Lower | Upper |
| *Act-5* | Equal variances assumed | 6.893 | .058 | -11.373 | 4 | .000 | -79.80667 | 7.01740 | -99.29008 | -60.32325 |
|  | Equal variances not assumed |  |  | -11.373 | 2.217 | .005 | -79.80667 | 7.01740 | -107.33748 | -52.27585 |

1. *Gtl-1*

| **Tests of Normality** | | | | | | | |
| --- | --- | --- | --- | --- | --- | --- | --- |
|  | Group | Kolmogorov-Smirnov^a^ | | | Shapiro-Wilk | | |
|  |  | Statistic | df | Sig. | Statistic | df | Sig. |
| *Gtl-1* | Control | .184 | 3 | . | .999 | 3 | .928 |
|  | BPA | .339 | 3 | . | .851 | 3 | .242 |
|  | BPA+BSHX | .176 | 3 | . | 1.000 | 3 | .976 |
| a. Lilliefors Significance Correction | | | | | | | |

- 1. Control VS BPA

| **Group Statistics** | | | | | |
| --- | --- | --- | --- | --- | --- |
|  | Group | N | Mean | Std. Deviation | Std. Error Mean |
| *Gtl-1* | Control | 3 | 100.2933 | 9.34664 | 5.39628 |
|  | BPA | 3 | 100.5567 | 5.34575 | 3.08637 |

| **Independent Samples Test** | | | | | | | | | | |
| --- | --- | --- | --- | --- | --- | --- | --- | --- | --- | --- |
|  | | Levene's Test for Equality of Variances | | t-test for Equality of Means | | | | | | |
|  |  | F | Sig. | t | df | Sig. (2-tailed) | Mean Difference | Std. Error Difference | 95% Confidence Interval of the Difference | |
|  |  |  |  |  |  |  |  |  | Lower | Upper |
| *Gtl-1* | Equal variances assumed | .516 | .512 | -.042 | 4 | .968 | -.26333 | 6.21655 | -17.52326 | 16.99659 |
|  | Equal variances not assumed |  |  | -.042 | 3.182 | .969 | -.26333 | 6.21655 | -19.42220 | 18.89553 |

6.2 BPA VS BPA+BSHX

| **Group Statistics** | | | | | |
| --- | --- | --- | --- | --- | --- |
|  | Group | N | Mean | Std. Deviation | Std. Error Mean |
| *Gtl-1* | BPA | 3 | 100.5567 | 5.34575 | 3.08637 |
|  | BPA+BSHX | 3 | 95.0967 | 3.95530 | 2.28360 |

| **Independent Samples Test** | | | | | | | | | | |
| --- | --- | --- | --- | --- | --- | --- | --- | --- | --- | --- |
|  | | Levene's Test for Equality of Variances | | t-test for Equality of Means | | | | | | |
|  |  | F | Sig. | t | df | Sig. (2-tailed) | Mean Difference | Std. Error Difference | 95% Confidence Interval of the Difference | |
|  |  |  |  |  |  |  |  |  | Lower | Upper |
| *Gtl-1* | Equal variances assumed | .706 | .448 | 1.422 | 4 | .228 | 5.46000 | 3.83933 | -5.19970 | 16.11970 |
|  | Equal variances not assumed |  |  | 1.422 | 3.685 | .234 | 5.46000 | 3.83933 | -5.56907 | 16.48907 |

1. *Nfm-1*

| **Tests of Normality** | | | | | | | |
| --- | --- | --- | --- | --- | --- | --- | --- |
|  | Group | Kolmogorov-Smirnov^a^ | | | Shapiro-Wilk | | |
|  |  | Statistic | df | Sig. | Statistic | df | Sig. |
| *Nfm-1* | Control | .192 | 3 | . | .997 | 3 | .894 |
|  | BPA | .183 | 3 | . | .999 | 3 | .931 |
|  | BPA+BSHX | .217 | 3 | . | .988 | 3 | .790 |
| a. Lilliefors Significance Correction | | | | | | | |

- 1. Control VS BPA

| **Group Statistics** | | | | | |
| --- | --- | --- | --- | --- | --- |
|  | Group | N | Mean | Std. Deviation | Std. Error Mean |
| *Nfm-1* | Control | 3 | 100.1033 | 5.54361 | 3.20061 |
|  | BPA | 3 | 165.1933 | 20.57328 | 11.87799 |

| **Independent Samples Test** | | | | | | | | | | |
| --- | --- | --- | --- | --- | --- | --- | --- | --- | --- | --- |
|  | | Levene's Test for Equality of Variances | | t-test for Equality of Means | | | | | | |
|  |  | F | Sig. | t | df | Sig. (2-tailed) | Mean Difference | Std. Error Difference | 95% Confidence Interval of the Difference | |
|  |  |  |  |  |  |  |  |  | Lower | Upper |
| *Nfm-1* | Equal variances assumed | 2.244 | .208 | -5.291 | 4 | .006 | -65.09000 | 12.30164 | -99.24484 | -30.93516 |
|  | Equal variances not assumed |  |  | -5.291 | 2.289 | .025 | -65.09000 | 12.30164 | -112.10593 | -18.07407 |

7.2 BPA VS BPA+BSHX

| **Group Statistics** | | | | | |
| --- | --- | --- | --- | --- | --- |
|  | Group | N | Mean | Std. Deviation | Std. Error Mean |
| *Nfm-1* | BPA | 3 | 165.1933 | 20.57328 | 11.87799 |
|  | BPA+BSHX | 3 | 160.2567 | 31.57993 | 18.23268 |

| **Independent Samples Test** | | | | | | | | | | |
| --- | --- | --- | --- | --- | --- | --- | --- | --- | --- | --- |
|  | | Levene's Test for Equality of Variances | | t-test for Equality of Means | | | | | | |
|  |  | F | Sig. | t | df | Sig. (2-tailed) | Mean Difference | Std. Error Difference | 95% Confidence Interval of the Difference | |
|  |  |  |  |  |  |  |  |  | Lower | Upper |
| *Nfm-1* | Equal variances assumed | .534 | .505 | .227 | 4 | .832 | 4.93667 | 21.76045 | -55.48003 | 65.35337 |
|  | Equal variances not assumed |  |  | .227 | 3.439 | .833 | 4.93667 | 21.76045 | -59.57692 | 69.45025 |

1. *Let-413*

| **Tests of Normality** | | | | | | | |
| --- | --- | --- | --- | --- | --- | --- | --- |
|  | Group | Kolmogorov-Smirnov^a^ | | | Shapiro-Wilk | | |
|  |  | Statistic | df | Sig. | Statistic | df | Sig. |
| *Let-413* | Control | .359 | 3 | . | .810 | 3 | .139 |
|  | BPA | .226 | 3 | . | .983 | 3 | .751 |
|  | BPA+BSHX | .290 | 3 | . | .926 | 3 | .475 |
| a. Lilliefors Significance Correction | | | | | | | |

- 1. Control VS BPA

| **Group Statistics** | | | | | |
| --- | --- | --- | --- | --- | --- |
|  | Group | N | Mean | Std. Deviation | Std. Error Mean |
| let_413 | Control | 3 | 100.3567 | 10.13897 | 5.85373 |
|  | BPA | 3 | 77.3100 | 32.32518 | 18.66295 |

| **Independent Samples Test** | | | | | | | | | | |
| --- | --- | --- | --- | --- | --- | --- | --- | --- | --- | --- |
|  | | Levene's Test for Equality of Variances | | t-test for Equality of Means | | | | | | |
|  |  | F | Sig. | t | df | Sig. (2-tailed) | Mean Difference | Std. Error Difference | 95% Confidence Interval of the Difference | |
|  |  |  |  |  |  |  |  |  | Lower | Upper |
| *Let-413* | Equal variances assumed | 2.624 | .181 | 1.178 | 4 | .304 | 23.04667 | 19.55945 | -31.25906 | 77.35240 |
|  | Equal variances not assumed |  |  | 1.178 | 2.390 | .343 | 23.04667 | 19.55945 | -49.23440 | 95.32774 |

- 1. BPA VS BPA+BSHX

| **Group Statistics** | | | | | |
| --- | --- | --- | --- | --- | --- |
|  | Group | N | Mean | Std. Deviation | Std. Error Mean |
| *Let-413* | BPA | 3 | 77.3100 | 32.32518 | 18.66295 |
|  | BPA+BSHX | 3 | 72.7700 | 3.95845 | 2.28541 |

| **Independent Samples Test** | | | | | | | | | | |
| --- | --- | --- | --- | --- | --- | --- | --- | --- | --- | --- |
|  | | Levene's Test for Equality of Variances | | t-test for Equality of Means | | | | | | |
|  |  | F | Sig. | t | df | Sig. (2-tailed) | Mean Difference | Std. Error Difference | 95% Confidence Interval of the Difference | |
|  |  |  |  |  |  |  |  |  | Lower | Upper |
| *Let-413* | Equal variances assumed | 4.723 | .095 | .241 | 4 | .821 | 4.54000 | 18.80236 | -47.66373 | 56.74373 |
|  | Equal variances not assumed |  |  | .241 | 2.060 | .831 | 4.54000 | 18.80236 | -74.14451 | 83.22451 |

1. *Par-6*

| **Tests of Normality** | | | | | | | |
| --- | --- | --- | --- | --- | --- | --- | --- |
|  | Group | Kolmogorov-Smirnov^a^ | | | Shapiro-Wilk | | |
|  |  | Statistic | df | Sig. | Statistic | df | Sig. |
| *Par-6* | Control | .374 | 3 | . | .777 | 3 | .061 |
|  | BPA | .260 | 3 | . | .958 | 3 | .605 |
|  | BPA+BSHX | .312 | 3 | . | .896 | 3 | .374 |
| a. Lilliefors Significance Correction | | | | | | | |

- 1. Control VS BPA

| **Group Statistics** | | | | | |
| --- | --- | --- | --- | --- | --- |
|  | Group | N | Mean | Std. Deviation | Std. Error Mean |
| *Par-6* | Control | 3 | 102.2867 | 25.05550 | 14.46580 |
|  | BPA | 3 | 86.7800 | 28.40617 | 16.40031 |

| **Independent Samples Test** | | | | | | | | | | |
| --- | --- | --- | --- | --- | --- | --- | --- | --- | --- | --- |
|  | | Levene's Test for Equality of Variances | | t-test for Equality of Means | | | | | | |
|  |  | F | Sig. | t | df | Sig. (2-tailed) | Mean Difference | Std. Error Difference | 95% Confidence Interval of the Difference | |
|  |  |  |  |  |  |  |  |  | Lower | Upper |
| *Par-6* | Equal variances assumed | .029 | .872 | .709 | 4 | .517 | 15.50667 | 21.86846 | -45.20992 | 76.22325 |
|  | Equal variances not assumed |  |  | .709 | 3.939 | .518 | 15.50667 | 21.86846 | -45.58504 | 76.59838 |

- 1. BPA VS BPA+BSHX

| **Group Statistics** | | | | | |
| --- | --- | --- | --- | --- | --- |
|  | Group | N | Mean | Std. Deviation | Std. Error Mean |
| *Par-6* | BPA | 3 | 86.7800 | 28.40617 | 16.40031 |
|  | BPA+BSHX | 3 | 109.0133 | 3.56993 | 2.06110 |

| **Independent Samples Test** | | | | | | | | | | |
| --- | --- | --- | --- | --- | --- | --- | --- | --- | --- | --- |
|  | | Levene's Test for Equality of Variances | | t-test for Equality of Means | | | | | | |
|  |  | F | Sig. | t | df | Sig. (2-tailed) | Mean Difference | Std. Error Difference | 95% Confidence Interval of the Difference | |
|  |  |  |  |  |  |  |  |  | Lower | Upper |
| *Par-6* | Equal variances assumed | 6.092 | .069 | -1.345 | 4 | .250 | -22.23333 | 16.52932 | -68.12608 | 23.65941 |
|  | Equal variances not assumed |  |  | -1.345 | 2.063 | .307 | -22.23333 | 16.52932 | -91.30704 | 46.84037 |

1. *Abts-4*

| **Tests of Normality** | | | | | | | |
| --- | --- | --- | --- | --- | --- | --- | --- |
|  | Group | Kolmogorov-Smirnov^a^ | | | Shapiro-Wilk | | |
|  |  | Statistic | df | Sig. | Statistic | df | Sig. |
| *Abts-4* | Control | .343 | 3 | . | .844 | 3 | .224 |
|  | BPA | .176 | 3 | . | 1.000 | 3 | .981 |
|  | BPA+BSHX | .200 | 3 | . | .995 | 3 | .863 |
| a. Lilliefors Significance Correction | | | | | | | |

- 1. Control VS BPA

| **Group Statistics** | | | | | |
| --- | --- | --- | --- | --- | --- |
|  | Group | N | Mean | Std. Deviation | Std. Error Mean |
| *Abts-4* | Control | 3 | 100.3867 | 4.27861 | 2.47026 |
|  | BPA | 3 | 92.7367 | 11.82056 | 6.82461 |

| **Independent Samples Test** | | | | | | | | | | |
| --- | --- | --- | --- | --- | --- | --- | --- | --- | --- | --- |
|  | | Levene's Test for Equality of Variances | | t-test for Equality of Means | | | | | | |
|  |  | F | Sig. | t | df | Sig. (2-tailed) | Mean Difference | Std. Error Difference | 95% Confidence Interval of the Difference | |
|  |  |  |  |  |  |  |  |  | Lower | Upper |
| *Abts-4* | Equal variances assumed | 1.359 | .308 | 1.054 | 4 | .351 | 7.65000 | 7.25792 | -12.50122 | 27.80122 |
|  | Equal variances not assumed |  |  | 1.054 | 2.515 | .382 | 7.65000 | 7.25792 | -18.18254 | 33.48254 |

- 1. BPA VS BPA+BSHX

| **Group Statistics** | | | | | |
| --- | --- | --- | --- | --- | --- |
|  | Group | N | Mean | Std. Deviation | Std. Error Mean |
| *Abts-4* | BPA | 3 | 92.7367 | 11.82056 | 6.82461 |
|  | BPA+BSHX | 3 | 184.4633 | 15.39953 | 8.89092 |

| **Independent Samples Test** | | | | | | | | | | |
| --- | --- | --- | --- | --- | --- | --- | --- | --- | --- | --- |
|  | | Levene's Test for Equality of Variances | | t-test for Equality of Means | | | | | | |
|  |  | F | Sig. | t | df | Sig. (2-tailed) | Mean Difference | Std. Error Difference | 95% Confidence Interval of the Difference | |
|  |  |  |  |  |  |  |  |  | Lower | Upper |
| *Abts-4* | Equal variances assumed | .201 | .677 | -8.184 | 4 | .001 | -91.72667 | 11.20820 | -122.84562 | -60.60771 |
|  | Equal variances not assumed |  |  | -8.184 | 3.749 | .002 | -91.72667 | 11.20820 | -123.68310 | -59.77024 |

1. *Pkc-3*

| **Tests of Normality** | | | | | | | |
| --- | --- | --- | --- | --- | --- | --- | --- |
|  | Group | Kolmogorov-Smirnov^a^ | | | Shapiro-Wilk | | |
|  |  | Statistic | df | Sig. | Statistic | df | Sig. |
| Pkc-3 | Control | .230 | 3 | . | .981 | 3 | .736 |
|  | BPA | .200 | 3 | . | .995 | 3 | .860 |
|  | BPA+BSHX | .262 | 3 | . | .956 | 3 | .598 |
| a. Lilliefors Significance Correction | | | | | | | |

- 1. Control VS BPA

| **Group Statistics** | | | | | |
| --- | --- | --- | --- | --- | --- |
|  | Group | N | Mean | Std. Deviation | Std. Error Mean |
| Pkc-3 | Control | 3 | 100.3233 | 9.72271 | 5.61341 |
|  | BPA | 3 | 189.7833 | 42.55956 | 24.57177 |

| **Independent Samples Test** | | | | | | | | | | |
| --- | --- | --- | --- | --- | --- | --- | --- | --- | --- | --- |
|  | | Levene's Test for Equality of Variances | | t-test for Equality of Means | | | | | | |
|  |  | F | Sig. | t | df | Sig. (2-tailed) | Mean Difference | Std. Error Difference | 95% Confidence Interval of the Difference | |
|  |  |  |  |  |  |  |  |  | Lower | Upper |
| Pkc-3 | Equal variances assumed | 2.890 | .164 | -3.549 | 4 | .024 | -89.46000 | 25.20481 | -159.43977 | -19.48023 |
|  | Equal variances not assumed |  |  | -3.549 | 2.208 | .061 | -89.46000 | 25.20481 | -188.67536 | 9.75536 |

- 1. BPA VS BPA+BSHX

| **Group Statistics** | | | | | |
| --- | --- | --- | --- | --- | --- |
|  | Group | N | Mean | Std. Deviation | Std. Error Mean |
| Pkc-3 | BPA | 3 | 189.7833 | 42.55956 | 24.57177 |
|  | BPA+BSHX | 3 | 169.7600 | 14.55594 | 8.40387 |

| **Independent Samples Test** | | | | | | | | | | |
| --- | --- | --- | --- | --- | --- | --- | --- | --- | --- | --- |
|  | | Levene's Test for Equality of Variances | | t-test for Equality of Means | | | | | | |
|  |  | F | Sig. | t | df | Sig. (2-tailed) | Mean Difference | Std. Error Difference | 95% Confidence Interval of the Difference | |
|  |  |  |  |  |  |  |  |  | Lower | Upper |
| Pkc-3 | Equal variances assumed | 1.944 | .236 | .771 | 4 | .484 | 20.02333 | 25.96916 | -52.07861 | 92.12527 |
|  | Equal variances not assumed |  |  | .771 | 2.462 | .508 | 20.02333 | 25.96916 | -73.85241 | 113.89907 |

1. *Mtm-6*

| **Tests of Normality** | | | | | | | |
| --- | --- | --- | --- | --- | --- | --- | --- |
|  | Group | Kolmogorov-Smirnov^a^ | | | Shapiro-Wilk | | |
|  |  | Statistic | df | Sig. | Statistic | df | Sig. |
| Mtm-6 | Control | .310 | 3 | . | .898 | 3 | .380 |
|  | BPA | .303 | 3 | . | .909 | 3 | .414 |
|  | BPA+BSHX | .210 | 3 | . | .991 | 3 | .819 |
| a. Lilliefors Significance Correction | | | | | | | |

- 1. Control VS BPA

| **Group Statistics** | | | | | |
| --- | --- | --- | --- | --- | --- |
|  | Group | N | Mean | Std. Deviation | Std. Error Mean |
| *Mtm-6* | Control | 3 | 100.1000 | 5.43346 | 3.13701 |
|  | BPA | 3 | 134.9800 | 21.30016 | 12.29765 |

| **Independent Samples Test** | | | | | | | | | | |
| --- | --- | --- | --- | --- | --- | --- | --- | --- | --- | --- |
|  | | Levene's Test for Equality of Variances | | t-test for Equality of Means | | | | | | |
|  |  | F | Sig. | t | df | Sig. (2-tailed) | Mean Difference | Std. Error Difference | 95% Confidence Interval of the Difference | |
|  |  |  |  |  |  |  |  |  | Lower | Upper |
| *Mtm-6* | Equal variances assumed | 5.804 | .074 | -2.748 | 4 | .051 | -34.88000 | 12.69146 | -70.11713 | .35713 |
|  | Equal variances not assumed |  |  | -2.748 | 2.259 | .097 | -34.88000 | 12.69146 | -83.90168 | 14.14168 |

- 1. BPA VS BPA+BSHX

| **Group Statistics** | | | | | |
| --- | --- | --- | --- | --- | --- |
|  | Group | N | Mean | Std. Deviation | Std. Error Mean |
| *Mtm-6* | BPA | 3 | 134.9800 | 21.30016 | 12.29765 |
|  | BPA+BSHX | 3 | 215.1233 | 47.99141 | 27.70785 |

| **Independent Samples Test** | | | | | | | | | | |
| --- | --- | --- | --- | --- | --- | --- | --- | --- | --- | --- |
|  | | Levene's Test for Equality of Variances | | t-test for Equality of Means | | | | | | |
|  |  | F | Sig. | t | df | Sig. (2-tailed) | Mean Difference | Std. Error Difference | 95% Confidence Interval of the Difference | |
|  |  |  |  |  |  |  |  |  | Lower | Upper |
| *Mtm-6* | Equal variances assumed | 1.368 | .307 | -2.644 | 4 | .057 | -80.14333 | 30.31431 | -164.30934 | 4.02268 |
|  | Equal variances not assumed |  |  | -2.644 | 2.759 | .085 | -80.14333 | 30.31431 | -181.57526 | 21.28859 |

1. *Par-3*

| **Tests of Normality** | | | | | | | |
| --- | --- | --- | --- | --- | --- | --- | --- |
|  | Group | Kolmogorov-Smirnov^a^ | | | Shapiro-Wilk | | |
|  |  | Statistic | df | Sig. | Statistic | df | Sig. |
| *Par-3* | Control | .326 | 3 | . | .874 | 3 | .308 |
|  | BPA | .233 | 3 | . | .979 | 3 | .724 |
|  | BPA+BSHX | .177 | 3 | . | 1.000 | 3 | .962 |
| a. Lilliefors Significance Correction | | | | | | | |

- 1. Control VS BPA

| **Group Statistics** | | | | | |
| --- | --- | --- | --- | --- | --- |
|  | Group | N | Mean | Std. Deviation | Std. Error Mean |
| *Par-3* | Control | 3 | 101.6167 | 22.92936 | 13.23827 |
|  | BPA | 3 | 116.8267 | 5.33554 | 3.08048 |

| **Independent Samples Test** | | | | | | | | | | |
| --- | --- | --- | --- | --- | --- | --- | --- | --- | --- | --- |
|  | | Levene's Test for Equality of Variances | | t-test for Equality of Means | | | | | | |
|  |  | F | Sig. | t | df | Sig. (2-tailed) | Mean Difference | Std. Error Difference | 95% Confidence Interval of the Difference | |
|  |  |  |  |  |  |  |  |  | Lower | Upper |
| *Par-3* | Equal variances assumed | 7.205 | .055 | -1.119 | 4 | .326 | -15.21000 | 13.59196 | -52.94732 | 22.52732 |
|  | Equal variances not assumed |  |  | -1.119 | 2.216 | .370 | -15.21000 | 13.59196 | -68.55550 | 38.13550 |

- 1. BPA VS BPA+BSHX

| **Group Statistics** | | | | | |
| --- | --- | --- | --- | --- | --- |
|  | Group | N | Mean | Std. Deviation | Std. Error Mean |
| *Par-3* | BPA | 3 | 116.8267 | 5.33554 | 3.08048 |
|  | BPA+BSHX | 3 | 141.3833 | 16.08314 | 9.28560 |

| **Independent Samples Test** | | | | | | | | | | |
| --- | --- | --- | --- | --- | --- | --- | --- | --- | --- | --- |
|  | | Levene's Test for Equality of Variances | | t-test for Equality of Means | | | | | | |
|  |  | F | Sig. | t | df | Sig. (2-tailed) | Mean Difference | Std. Error Difference | 95% Confidence Interval of the Difference | |
|  |  |  |  |  |  |  |  |  | Lower | Upper |
| *Par-3* | Equal variances assumed | 1.665 | .266 | -2.510 | 4 | .066 | -24.55667 | 9.78324 | -51.71929 | 2.60596 |
|  | Equal variances not assumed |  |  | -2.510 | 2.435 | .106 | -24.55667 | 9.78324 | -60.20586 | 11.09253 |

1. *Erm-1*

| **Tests of Normality** | | | | | | | |
| --- | --- | --- | --- | --- | --- | --- | --- |
|  | Group | Kolmogorov-Smirnov^a^ | | | Shapiro-Wilk | | |
|  |  | Statistic | df | Sig. | Statistic | df | Sig. |
| *Erm-1* | Control | .299 | 3 | . | .914 | 3 | .432 |
|  | BPA | .298 | 3 | . | .915 | 3 | .436 |
|  | BPA+BSHX | .337 | 3 | . | .853 | 3 | .250 |
| a. Lilliefors Significance Correction | | | | | | | |

- 1. Control VS BPA

| **Group Statistics** | | | | | |
| --- | --- | --- | --- | --- | --- |
|  | Group | N | Mean | Std. Deviation | Std. Error Mean |
| *Erm-1* | Control | 3 | 100.7733 | 15.63704 | 9.02805 |
|  | BPA | 3 | 172.2333 | 28.83716 | 16.64914 |

| **Independent Samples Test** | | | | | | | | | | |
| --- | --- | --- | --- | --- | --- | --- | --- | --- | --- | --- |
|  | | Levene's Test for Equality of Variances | | t-test for Equality of Means | | | | | | |
|  |  | F | Sig. | t | df | Sig. (2-tailed) | Mean Difference | Std. Error Difference | 95% Confidence Interval of the Difference | |
|  |  |  |  |  |  |  |  |  | Lower | Upper |
| *Erm-1* | Equal variances assumed | 1.743 | .257 | -3.773 | 4 | .020 | -71.46000 | 18.93937 | -124.04411 | -18.87589 |
|  | Equal variances not assumed |  |  | -3.773 | 3.083 | .031 | -71.46000 | 18.93937 | -130.83051 | -12.08949 |

- 1. BPA VS BPA+BSHX

| **Group Statistics** | | | | | |
| --- | --- | --- | --- | --- | --- |
|  | Group | N | Mean | Std. Deviation | Std. Error Mean |
| *Erm-1* | BPA | 3 | 172.2333 | 28.83716 | 16.64914 |
|  | BPA+BSHX | 3 | 174.0800 | 33.02620 | 19.06769 |

| **Independent Samples Test** | | | | | | | | | | |
| --- | --- | --- | --- | --- | --- | --- | --- | --- | --- | --- |
|  | | Levene's Test for Equality of Variances | | t-test for Equality of Means | | | | | | |
|  |  | F | Sig. | t | df | Sig. (2-tailed) | Mean Difference | Std. Error Difference | 95% Confidence Interval of the Difference | |
|  |  |  |  |  |  |  |  |  | Lower | Upper |
| *Erm-1* | Equal variances assumed | .144 | .724 | -.073 | 4 | .945 | -1.84667 | 25.31345 | -72.12806 | 68.43473 |
|  | Equal variances not assumed |  |  | -.073 | 3.929 | .945 | -1.84667 | 25.31345 | -72.63468 | 68.94135 |

**Fig. 5 B**

1. *Clc-2*

| **Tests of Normality** | | | | | | | |
| --- | --- | --- | --- | --- | --- | --- | --- |
|  | Group | Kolmogorov-Smirnov^a^ | | | Shapiro-Wilk | | |
|  |  | Statistic | df | Sig. | Statistic | df | Sig. |
| *Clc-2* | BPA+BSHX | .201 | 3 | . | .994 | 3 | .856 |
|  | BPA+BSHX+*hsp-16.2* RNAi | .255 | 3 | . | .963 | 3 | .630 |
| a. Lilliefors Significance Correction | | | | | | | |

| **Group Statistics** | | | | | |
| --- | --- | --- | --- | --- | --- |
|  | Group | N | Mean | Std. Deviation | Std. Error Mean |
| *Clc-2* | BPA+BSHX | 3 | 102.5944 | 2.49293 | 1.43929 |
|  | BPA+BSHX+*hsp-16.2* RNAi | 3 | 82.4451 | 6.74411 | 3.89372 |

| **Independent Samples Test** | | | | | | | | | | |
| --- | --- | --- | --- | --- | --- | --- | --- | --- | --- | --- |
|  | | Levene's Test for Equality of Variances | | t-test for Equality of Means | | | | | | |
|  |  | F | Sig. | t | df | Sig. (2-tailed) | Mean Difference | Std. Error Difference | 95% Confidence Interval of the Difference | |
|  |  |  |  |  |  |  |  |  | Lower | Upper |
| *Clc-2* | Equal variances assumed | 2.758 | .172 | 4.854 | 4 | .008 | 20.14923 | 4.15122 | 8.62361 | 31.67485 |
|  | Equal variances not assumed |  |  | 4.854 | 2.537 | .024 | 20.14923 | 4.15122 | 5.46187 | 34.83659 |

1. *Ifb-2*

| **Tests of Normality** | | | | | | | |
| --- | --- | --- | --- | --- | --- | --- | --- |
|  | Group | Kolmogorov-Smirnov^a^ | | | Shapiro-Wilk | | |
|  |  | Statistic | df | Sig. | Statistic | df | Sig. |
| *Ifb-2* | BPA+BSHX | .205 | 3 | . | .993 | 3 | .839 |
|  | BPA+BSHX+*hsp-16.2* RNAi | .365 | 3 | . | .796 | 3 | .106 |
| a. Lilliefors Significance Correction | | | | | | | |

| **Group Statistics** | | | | | |
| --- | --- | --- | --- | --- | --- |
|  | Group | N | Mean | Std. Deviation | Std. Error Mean |
| *Ifb-2* | BPA+BSHX | 3 | 104.9114 | 4.36732 | 2.52147 |
|  | BPA+BSHX+*hsp-16.2* RNAi | 3 | 164.9553 | 10.69404 | 6.17421 |

| **Independent Samples Test** | | | | | | | | | | |
| --- | --- | --- | --- | --- | --- | --- | --- | --- | --- | --- |
|  | | Levene's Test for Equality of Variances | | t-test for Equality of Means | | | | | | |
|  |  | F | Sig. | t | df | Sig. (2-tailed) | Mean Difference | Std. Error Difference | 95% Confidence Interval of the Difference | |
|  |  |  |  |  |  |  |  |  | Lower | Upper |
| *Ifb-2* | Equal variances assumed | 4.416 | .103 | -9.003 | 4 | .001 | -60.04398 | 6.66923 | -78.56073 | -41.52723 |
|  | Equal variances not assumed |  |  | -9.003 | 2.649 | .005 | -60.04398 | 6.66923 | -82.95123 | -37.13673 |

1. *Act-5*

| **Tests of Normality** | | | | | | | |
| --- | --- | --- | --- | --- | --- | --- | --- |
|  | Group | Kolmogorov-Smirnov^a^ | | | Shapiro-Wilk | | |
|  |  | Statistic | df | Sig. | Statistic | df | Sig. |
| *Act-5* | BPA+BSHX | .250 | 3 | . | .967 | 3 | .652 |
|  | BPA+BSHX+*hsp-16.2* RNAi | .251 | 3 | . | .966 | 3 | .647 |
| a. Lilliefors Significance Correction | | | | | | | |

| **Group Statistics** | | | | | |
| --- | --- | --- | --- | --- | --- |
|  | Group | N | Mean | Std. Deviation | Std. Error Mean |
| *Act-5* | BPA+BSHX | 3 | 98.9988 | 3.12860 | 1.80630 |
|  | BPA+BSHX+*hsp-16.2* RNAi | 3 | 128.5161 | 17.36472 | 10.02553 |

| **Independent Samples Test** | | | | | | | | | | |
| --- | --- | --- | --- | --- | --- | --- | --- | --- | --- | --- |
|  | | Levene's Test for Equality of Variances | | t-test for Equality of Means | | | | | | |
|  |  | F | Sig. | t | df | Sig. (2-tailed) | Mean Difference | Std. Error Difference | 95% Confidence Interval of the Difference | |
|  |  |  |  |  |  |  |  |  | Lower | Upper |
| *Act-5* | Equal variances assumed | 4.918 | .091 | -2.898 | 4 | .044 | -29.51728 | 10.18695 | -57.80078 | -1.23378 |
|  | Equal variances not assumed |  |  | -2.898 | 2.130 | .094 | -29.51728 | 10.18695 | -70.88684 | 11.85228 |

1. *Dlg-1*

| **Tests of Normality** | | | | | | | |
| --- | --- | --- | --- | --- | --- | --- | --- |
|  | Group | Kolmogorov-Smirnov^a^ | | | Shapiro-Wilk | | |
|  |  | Statistic | df | Sig. | Statistic | df | Sig. |
| *Dlg-1* | BPA+BSHX | .308 | 3 | . | .902 | 3 | .393 |
|  | BPA+BSHX+*hsp-16.2* RNAi | .213 | 3 | . | .990 | 3 | .808 |
| a. Lilliefors Significance Correction | | | | | | | |

| **Group Statistics** | | | | | |
| --- | --- | --- | --- | --- | --- |
|  | Group | N | Mean | Std. Deviation | Std. Error Mean |
| *Dlg-1* | BPA+BSHX | 3 | 104.3193 | 10.04064 | 5.79697 |
|  | BPA+BSHX+*hsp-16.2* RNAi | 3 | 101.8406 | 5.30039 | 3.06018 |

| **Independent Samples Test** | | | | | | | | | | |
| --- | --- | --- | --- | --- | --- | --- | --- | --- | --- | --- |
|  | | Levene's Test for Equality of Variances | | t-test for Equality of Means | | | | | | |
|  |  | F | Sig. | t | df | Sig. (2-tailed) | Mean Difference | Std. Error Difference | 95% Confidence Interval of the Difference | |
|  |  |  |  |  |  |  |  |  | Lower | Upper |
| *Dlg-1* | Equal variances assumed | 1.992 | .231 | .378 | 4 | .725 | 2.47878 | 6.55512 | -15.72114 | 20.67870 |
|  | Equal variances not assumed |  |  | .378 | 3.034 | .730 | 2.47878 | 6.55512 | -18.24952 | 23.20708 |

1. *Abts-4*

| **Tests of Normality** | | | | | | | |
| --- | --- | --- | --- | --- | --- | --- | --- |
|  | Group | Kolmogorov-Smirnov^a^ | | | Shapiro-Wilk | | |
|  |  | Statistic | df | Sig. | Statistic | df | Sig. |
| *Abts-4* | BPA+BSHX | .303 | 3 | . | .908 | 3 | .412 |
|  | BPA+BSHX+*hsp-16.2* RNAi | .337 | 3 | . | .854 | 3 | .250 |
| a. Lilliefors Significance Correction | | | | | | | |

| **Group Statistics** | | | | | |
| --- | --- | --- | --- | --- | --- |
|  | Group | N | Mean | Std. Deviation | Std. Error Mean |
| *Abts-4* | BPA+BSHX | 3 | 99.6086 | 6.21788 | 3.58989 |
|  | BPA+BSHX+*hsp-16.2* RNAi | 3 | 101.1413 | 3.82387 | 2.20771 |

| **Independent Samples Test** | | | | | | | | | | |
| --- | --- | --- | --- | --- | --- | --- | --- | --- | --- | --- |
|  | | Levene's Test for Equality of Variances | | t-test for Equality of Means | | | | | | |
|  |  | F | Sig. | t | df | Sig. (2-tailed) | Mean Difference | Std. Error Difference | 95% Confidence Interval of the Difference | |
|  |  |  |  |  |  |  |  |  | Lower | Upper |
| *Abts-4* | Equal variances assumed | 1.200 | .335 | -.364 | 4 | .734 | -1.53273 | 4.21442 | -13.23383 | 10.16838 |
|  | Equal variances not assumed |  |  | -.364 | 3.323 | .738 | -1.53273 | 4.21442 | -14.23576 | 11.17031 |

**Fig. 5 C**

| **Tests of Normality** | | | | | | | | | | |
| --- | --- | --- | --- | --- | --- | --- | --- | --- | --- | --- |
|  | Group | Kolmogorov-Smirnov^a^ | | | | Shapiro-Wilk | | | | |
|  |  | Statistic | df | | Sig. | Statistic | | df | | Sig. |
| Relative fluoresence intensity | Control | .245 | 10 | | .092 | .908 | | 10 | | .270 |
|  | BPA | .174 | 8 | | .200^*^ | .929 | | 8 | | .509 |
|  | BPA+BSHX | .273 | 9 | | .052 | .802 | | 9 | | .021 |
|  | BPA+BSHX+*hsp-16.2* RNAi | .111 | 13 | | .200^*^ | .952 | | 13 | | .623 |
| *. This is a lower bound of the true significance. | | | | | | | | | | |
| a. Lilliefors Significance Correction | | | | | | | | | | |
| **Hypothesis Test Summary** | | | | | | | | | | |
|  | Null Hypothesis | | | Test | | | Sig.^a,b^ | | Decision | |
| 1 | The distribution of relative fluoresence intensity is the same across categories of Group. | | | Independent-Samples Kruskal-Wallis Test | | | .000 | | Reject the null hypothesis. | |
| a. The significance level is .050. | | | | | | | | | | |
| b. Asymptotic significance is displayed. | | | | | | | | | | |

| **Independent-Samples Kruskal-Wallis Test Summary** | |
| --- | --- |
| Total N | 40 |
| Test Statistic | 26.674^a^ |
| Degree Of Freedom | 3 |
| Asymptotic Sig.(2-sided test) | .000 |
| a. The test statistic is adjusted for ties. | |

| **Pairwise Comparisons of Group** | | | | | |
| --- | --- | --- | --- | --- | --- |
| Sample 1-Sample 2 | Test Statistic | Std. Error | Std. Test Statistic | Sig. | Adj. Sig.^a^ |
| BPA+BSHX—Control | 1.000 | 5.371 | .186 | .852 | 1.000 |
| BPA+BSHX—BPA | 17.750 | 5.681 | 3.125 | .002 | .011 |
| BPA+BSHX—BPA+BSHX+*hsp-16.2* RNAi | -20.615 | 5.069 | -4.067 | .000 | .000 |
| Control—BPA | -16.750 | 5.545 | -3.021 | .003 | .015 |
| Control—BPA+BSHX+*hsp-16.2* RNAi | -19.615 | 4.917 | -3.989 | .000 | .000 |
| 2.00-BPA+BSHX+*hsp-16.2* RNAi | -2.865 | 5.253 | -.545 | .585 | 1.000 |
| Each row tests the null hypothesis that the Sample 1 and Sample 2 distributions are the same.  Asymptotic significances (2-sided tests) are displayed. The significance level is .050. | | | | | |
| a. Significance values have been adjusted by the Bonferroni correction for multiple tests. | | | | | |
